# Structure, Stability, and Spin Resonance in Dicopper(II) Complexes

**DOI:** 10.1021/acs.inorgchem.6c00933

**Published:** 2026-07-06

**Authors:** Ökten Üngör, Nicholas Yiching Chiang, Alexander Yu Sokolov, Joseph M. Zadrozny

**Affiliations:** Department of Chemistry and Biochemistry, 2647 The Ohio State University, Columbus, Ohio 43210, United States

## Abstract

Designing molecular systems that access entangled spin states through spin-forbidden transitions is central to advancing molecular platforms for quantum sensing and information science, yet remains a fundamental challenge. Herein, we examine a family of dinuclear Cu­(II) complexes [L_2_Cu_2_(μ-CA)]­X_2_, where systematic variation of cyclic (Me_3_tacn and tmchd) and polypyridyl ligands (4′-Br/Cl-terpy and tpa), along with different counteranions (X = ClO_4_^–^, CF_3_SO_3_^–^, and Cl^–^), tunes local geometry, exchange coupling, and EPR spectra. Structural analysis reveals distorted octahedral to trigonal-bipyramidal Cu­(II) environments, and magnetic susceptibility measurements establish moderate antiferromagnetic coupling (with singlet-to-triplet energy gaps spanning 2*J* = −1.26 to −30.68(3) cm^–1^). Low-temperature EPR spectra display well-resolved half-field (Δ*M*_s_ = ±2) transitions within the *S* = 1 manifold, with frozen-solution EPR confirming persistence of the [L_2_Cu_2_(μ-CA)]^2+^ dinuclear core. Comparison to a mononuclear analogue isolates bridge-mediated magnetic effects, and multireference calculations capture trends in the *g* anisotropy. Overall, we reveal a counterintuitively robust magnetic core for the complexes to changes in counterions and ligand shells. These chemical changes have subtle influences on the intensities of the forbidden Δ*M*_S_ = ±2 transition in the excited triplet state. Importantly, our extensive investigation also reveals a remarkable absence of the singlet/triplet transition, which provides hints at future design strategies.

## Graphical abstract

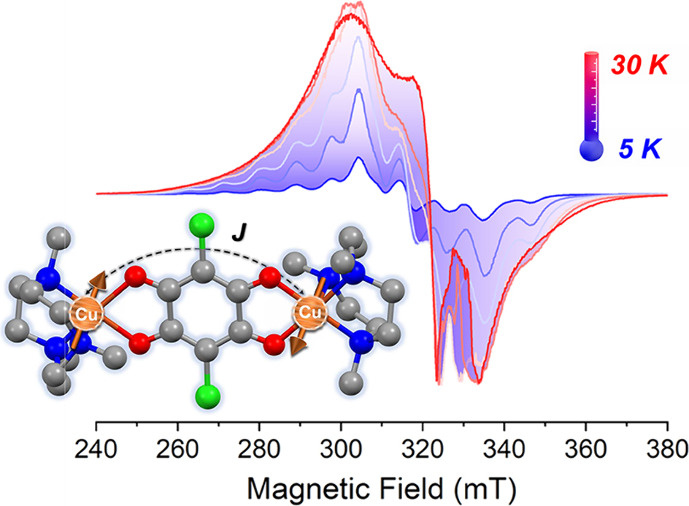

## Introduction

Two-spin molecular systems, particularly those composed of exchange-coupled *S* = 1/2 centers, are a key interest in quantum sensing and information science.^1–5^ Their spin manifolds comprise singlet (*S* = 0) and a triplet (*S* = 1) states, with relative energies defined by the strength and sign of exchange coupling, *J*. The interest in these systems for QIS is in part because of their ability to support spin entanglement.^6,7^ Entangled states may exhibit novel sensitivities to local environments or enable new mechanisms of spectral control/readout, which makes them attractive for two-qubit logic operations^8–11^ or tunable magnetic resonance sensing^12,13^ through electron paramagnetic resonance (EPR) spectroscopy. Realizing these applications requires a molecular understanding of how to control these spin manifolds and the many different types of magnetic resonance transitions that are possible within them.

A challenge in using two-spin systems for any of the above applications is that not every EPR transition can be readily addressed because of selection rules. For example, the singlet-to-triplet (Δ*S* = ±1) transition is forbidden by the Δ*M*_S_ = ±1 selection rule and is hence spectroscopically inaccessible under standard conditions.^14–16^ Separately the Δ*M*_S_ = ±2 (“half-field transition”) is typically extremely weak. In a simplified picture of the *S* = 1 manifold arising from two coupled *S* = 1/2 centers, only two of the three possible transitions correspond to Δ*M*_S_ = ±1 processes (Scheme 1). Hence, only a subset of nominally accessible transitions is observable in practice, although such “forbidden” transitions may gain intensity through spin-state mixing depending on the spin Hamiltonian.^5^ Thus spectroscopic control of the intrinsic spin entanglement in these species remains a lofty goal.

**Scheme 1:**
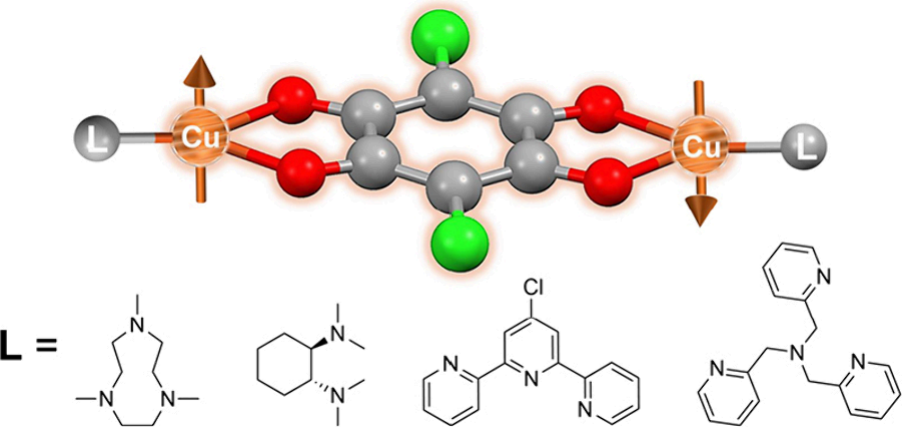
Schematic Representation of Ligand-Tuned Magnetic Exchange in Dinuclear Cu­(II) Complexes Bridged by Chloroanilate Dianion^a^

a The nitrogen donor ligands range from aliphatic to aromatic systems, enabling systematic tuning of the coordination environment and super-exchange pathways. The counterions used in this study are perchlorate (ClO_4_^–^), triflate (CF_3_SO_3_^–^), and chloride (Cl^–^).

Overcoming this limitation requires a molecule-level understanding of how to tune the EPR selection rules for forbidden transitions.^14,17–24^ In practice, this need necessitates an intuition for the spin interactions that govern transition intensity, e.g., exchange coupling (*J*),^25–27^ zero-field splitting (*D*)^28–30^ and hyperfine interactions (*A*).^31,32^ A separate critical parameter is the relative orientation of the *g-*tensor axes on each spin center, which is posited to enable forbidden transition intensity.^33^ This four-dimensional problem is incredibly complicated from a molecular design perspective. On top of this complexity, computations used to interrogate and understand thee interactions become increasingly expensive because of the generally large molecular size.

Equally important for advancing two-spin systems is chemical stability across diverse environments. Most multispin systems have been examined in single crystals, where long-range structural order enables powerful clarification of structure–function relationships.^6,34–36^ While insightful, this approach avoids the challenge of assessing solution stability, which would be necessary in any solution-based QIS application. Indeed, many metal complexes can distort, dissociate, coordinate solvents, or otherwise structurally change outside the crystalline state, and this has been explicitly observed, for example, in dinuclear V­(IV) complexes.^37^

Herein we report a synthetic, spectroscopic, magnetic, and computational analysis of dicopper­(II) complexes featuring chloranilate (CA^2–^) as a bridging ligand (Figure 1): [L_2_Cu_2_(μ-CA)]­X_2_, where L = Me_3_tacn (**1–3**), tmchd (**4–6**) and polypyridyl ligands 4^′^-Br/Cl-terpy and tpa (**7–9**), along with different counteranions (X = ClO_4_^–^, CF_3_SO_3_^–^, and Cl^–^). Here, we originally sought to investigate how ligand identity and counterion influence forbidden transitions, inspired by decades-agoreports of singlet–triplet EPR transitions in the dicopper complexes [Cu_2_(tpy)_2_(μ-CA)]­(PF_6_)_2_^4^ and [Cu_2_(tren)_2_(SCN)_2_][Bibr ref31] which were reported to have small |*J*| ≈ 0.01–0.06 cm^–1^ (or 2*J* ≈ 0.02–0.12 cm^–1^) determined by magnetometry. In parallel, we provide the first tests of the capability of multireference computational analyses to reproduce our experimental observations and resolve *g*-tensor orientations.

**Figure 1:**
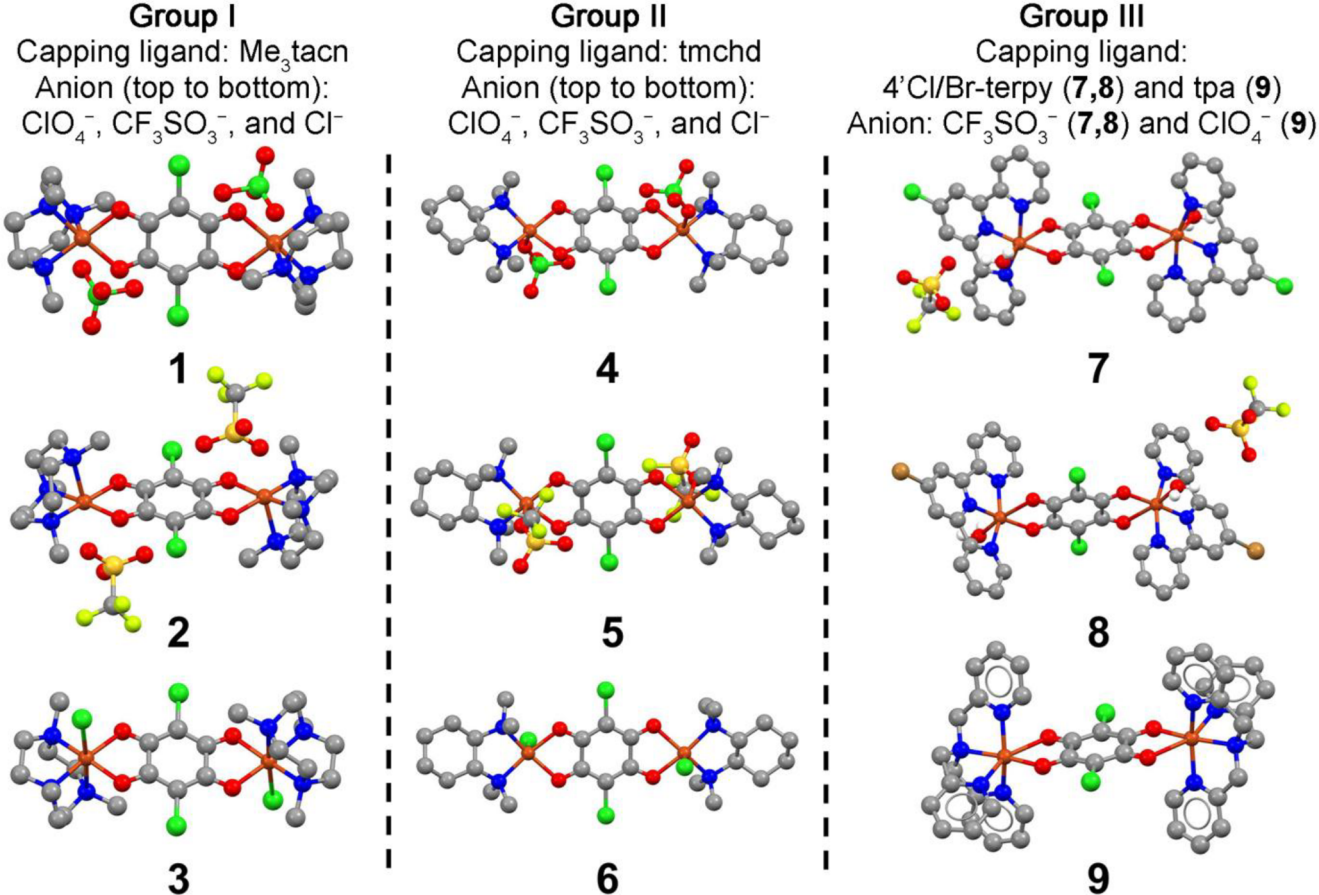
Crystal structures of the dinuclear copper­(II) complexes discussed in this manuscript, featuring various ancillary ligands and counterions. The crystal structure of **9** was taken from a previous report.^37^ Most hydrogens are omitted for clarity. Color scheme: Cu (orange), O (red), Cl (green), Br (brown), S (yellow), F (light green), N (blue), and C (gray).

Complexes **1**-**6** exhibit antiferromagnetic coupling (2*J* = 1.26 to −30.68 cm^–1^), yielding singlet (*S* = 0) ground states confirmed by variable-temperature susceptibility fits (Table 2). Despite these seemingly well-isolated ground states, the X-band EPR spectra display features of the excited *S* = 1 triplet state, including well-resolved Δ*M*_s_ = ±2 half-field transitions. Spectral simulations reveal modest anisotropies (|*D*| ≈ 147–210 MHz) and hyperfine couplings (*A*_iso_ ≈ 70–250 MHz) for these triplet states. Subtle trends emerge that collectively illustrate how small geometric and electronic perturbations from ligand choice propagate into the spin Hamiltonians of these systems. Computational analyses of **1** and Cu­(Me_3_tacn)­(CA) **1a**, a mononuclear fragment of **1**, resolve *g*-axis orientations to better understand the EPR spectral properties that we observe. Importantly, while we were able to identify the forbidden half-field transitions in all complexes, we were unable to reproduce the initial observations^4,31^ of the single-triplet transition in any of the studied complexes, including **7** and **9** which are chemically very similar to the original system. We discuss the possible origins of this lack of reproducibility at length toward the end of the manuscript.

## Materials and Methods

### General Considerations

Complex **1** was synthesized following the procedure outlined in the previous report by Chaudhuri.^25^ Complexes **1a** and **2–8** were synthesized with minor modifications to this procedure, while complex **9** was synthesized according to the original method.^38^ Copper­(II) starting materials were purchased from the following vendors and used as received: [Cu­(H_2_O)_6_]­(ClO_4_)_2_ and CuCl_2_ from Thermo Fischer Scientific, Cu­(CF_3_SO_3_)_2_ from TCI Chemicals, and Cu­(NO_3_)_2_·3H_2_O from Acros Organics. Ligands were sourced as follows: 1,4,7-trimethyl-1,4,7-triazacyclononane (Me_3_tacn) and (1*R*,2*R*)-*N*1,*N*1,*N*2,*N*2-tetramethylcyclohexane-1,2-diamine (tmchd) from Ambeed, chloroanilic acid (CA) from Alfa Aesar, 4′-bromo-2,2′:6′,2″-terpyridine (Br-terpy) and 4′-chloro-2,2′:6′,2′′-terpyridine (Cl-terpy) from Arron Chemicals, and tris­(2-pyridylmethyl)­amine (tpa) from Sigma Aldrich. The purity of the ligands was confirmed via ^1^H NMR prior to use. For spectroscopic measurements, freshly prepared solutions of the complexes were made in acetonitrile (MeCN), ethanol (EtOH), *n*-butyronitrile (BuCN), dimethyl sulfoxide (DMSO) and dichloromethane (DCM) as specified in the respective experimental sections for UV–vis and EPR. Solvents were HPLC grade and used as received. All reactions were carried out under ambient conditions.

### Safety Considerations

Complexes incorporating perchlorate anions are potentially explosive; therefore, all perchlorate-containing compounds were synthesized in small quantities (typically <200 mg per synthesis) and handled with appropriate precautions.^39^ Other than the presence of ClO_4_^–^, no unusual hazards were noted.

### [Cu_2_(Me_3_tacn)_2_(μ-CA)]­(ClO_4_)_2_ (1)

The synthesis of complex **1** was carried out following the previously reported method.^25^ A clear blue solution of [Cu­(H_2_O)_6_]­(ClO_4_)_2_ (185 mg, 0.5 mmol) in 15 mL of methanol (MeOH) was prepared, to which a solution of Me_3_tacn (86 mg, 0.5 mmol) in 15 mL of MeOH was added, resulting in a teal-blue solution. The mixture was stirred at room temperature for 15 min. Next, chloroanilic acid (CA) (52 mg, 0.25 mmol) was added as a solid in one portion, immediately turning the solution dark brown. After stirring for an additional 15 min, solid sodium perchlorate (61 mg, 0.5 mmol) was added, and the mixture was stirred for five more minutes. The reaction flask was then left undisturbed at 7 °C overnight. Very dark brown/black crystals formed the next day and were collected by vacuum filtration, washed with 10 mL of cold MeOH, and dried under vacuum. It is worth noting that the addition of sodium perchlorate is optional; while included in the original procedure, complex **1** can also be synthesized without it. (160 mg, 72.3% yield) anal. calcd. (found) for C_24_H_42_Cu_2_N_6_Cl_4_O_12_: 32.92 (32.65) %C, 9.60 (9.55) %N and 4.84 (4.85) % H. UV–vis (BuCN, 1 mM, Figures S8 and S9) λ_max_ (nm) (ε_M_ (M^–1^ cm^–1^)): 292(22), 252(30), and 530(55).

### Cu­(Me_3_tacn)­(μ-CA) (1a)

To a solution of Cu­(NO_3_)_2_·3H_2_O (24 mg, 0.1 mmol) in 1 mL deionized water (DI H_2_O), a purple suspension of chloroanilic acid (21 mg, 0.1 mmol) in 3 mL DI H_2_O was added while stirring vigorously at 1400 rpm, resulting in a green suspension. To this suspension, a solution of Me_3_tacn (17 mg, 0.1 mmol) in 6 mL of MeCN was added, yielding a clear, violet-colored solution. The mixture was stirred at room temperature for 30 min, then filtered and left undisturbed at 7 °C. Violet, rod-like crystals formed after one week and were collected by filtration (19.5 mg, 44.2% yield) anal. calcd. (found) for C_15_H_21_CuN_3_O_4_Cl_2_ 40.78 (40.41) %C, 9.51 (9.33) %N and 4.79 (4.63) % H. UV–Vis (BuCN, 1 mM, Figure S10) λ_max_ (nm) (ε_M_ (M^–1^ cm^–^^1^)): 327­(43) and 521(62).

### [Cu_2_(Me_3_tacn)_2_(μ-CA)]­(CF_3_SO_3_)_2_ (2)

To a blue solution of Cu­(CF_3_SO_3_)_2_ (362 mg, 1.0 mmol) in 20 mL of acetonitrile (MeCN), solution of Me_3_tacn (171 mg, 1.0 mmol) in MeCN was added, resulting in a dark blue solution. The mixture was stirred at room temperature for 10 min. Chloroanilic acid (CA) (105 mg, 0.5 mmol) was then added as a solid, which immediately turned the solution dark brown. The mixture was stirred for an additional 30 min before being layered with diethyl ether (Et_2_O) and left undisturbed at room temperature. Dark green crystals formed after two days and were collected by vacuum filtration, washed with 10 mL of Et_2_O, and dried under vacuum. (211 mg, 43.3% yield) Anal. Calcd. (Found) for C_26_H_42_Cu_2_N_6_S_2_Cl_2_O_10_F_6_: 32.04 (31.68) %C, 8.62 (8.40) %N and 4.34 (4.26) % H. UV–vis (BuCN, 1 mM, Figure S11) λ_max_ (nm) (ε_M_ (M^–1^ cm^–^^1^)): 268(74), 354(29), and 631(78).

### [Cu_2_(Me_3_tacn)_2_(μ-CA)­Cl_2_]·H_2_O (3)

CuCl_2_ (135 mg, 1.0 mmol) and Me_3_tacn (171 mg, 1.0 mmol) were combined in a flask and dissolved in 30 mL of methanol (MeOH). Chloroanilic acid (CA) (105 mg, 0.5 mmol) was then added to the solution, which was stirred at room temperature for 30 min. The resulting solution was carefully layered with Et_2_O and left undisturbed at room temperature. The layered solution mixture yielded three distinct types of crystals: yellow prisms, identified as {[Cu_2_(Me_3_tacn)_2_(Cl)_2_(CA)]­(Cl)_*n*_; greenish-yellow, plate-like crystals, confirmed to be Cu­(Me_3_tacn)­Cl_2_; and the green, plate-like target crystals of **3**. Anal. Calcd. (Found) for C_24_H_46_Cu_2_N_6_Cl_4_O_5_·1MeOH: 37.56 (37.68) %C, 10.95 (11.78) %N and 6.04 (6.96) % H. Although a few single crystals of compound **3** were isolated, the yield was too low (<5 mg) to enable bulk characterization. However, a UV–vis spectrum of the crude reaction mixture containing **3** was recorded (Figure S12) and enabled qualitative comparison to the other Me_3_tacn analogues.

### [Cu_2_(tmchd)_2_(μ-CA)]­(ClO_4_)_2_ (4)

[Cu­(H_2_O)_6_]­(ClO_4_)_2_ (185.3 mg, 0.5 mmol) and tmchd (85 mg, 0.5 mmol) were combined in a flask and dissolved in 20 mL of MeOH. Chloroanilic acid (CA) (105 mg, 0.5 mmol) was then added to the solution, which was stirred at room temperature for 30 min. Upon the addition of CA, the initially blue/violet clear solution immediately transformed into a dark green suspension. The mixture was filtered, and the filtrate was left to evaporate slowly at room temperature. Green block crystals formed the next day. These crystals were collected by vacuum filtration, washed with 10 mL of Et_2_O, and dried under vacuum. (131 mg, 29.8% yield) anal. calcd. (found) for C_26_H_44_Cu_2_N_4_Cl_4_O_12_: 35.75 (35.61) %C, 6.41 (6.34) %N and 5.08 (5.01) %H. UV–Vis (BuCN, 1 mM, Figure S13) λ_max_ (nm) (ε_M_ (M^–1^ cm^–1^)): 286(32), 355(22), and 610(44).

### [Cu_2_(tmchd)_2_(μ-CA)]­(CF_3_SO_3_)_2_ (5)

Cu­(CF_3_SO_3_)_2_ (180.8 mg, 0.5 mmol) and tmchd (85 mg, 0.5 mmol) were combined in a flask and dissolved in 20 mL of MeCN, resulting in a dark blue clear solution. CA (105 mg, 0.5 mmol) was then added, and the mixture was stirred at room temperature for 30 min. Immediately after the addition, the solution’s color changed to grayish blue suspension. The mixture was filtered, and the filtrate was left to evaporate slowly. (145 mg, 29.2% yield) anal. calcd. (found) for C_28_H_44_Cu_2_N_4_Cl_2_O_10_F_6_S_2_: 34.57 (34.09) %C, 5.76 (5.45) %-N and 4.56 (4.21) % H. UV–Vis (BuCN, 1 mM, Figure S14) λ_max_ (nm) (ε_M_ (M^–1^ cm^–1^)): 278(65), 358(23), and 601(45).

### [Cu_2_(tmchd)_2_(μ-CA)­Cl_2_]·H_2_O (6)

CuCl_2_ (135 mg, 1 mmol) and tmchd (180 mg, 1 mmol) are mixed together in a flask and dissolved in 30 mL of MeOH. Then to this solution added chloroanilic acid (CA) (100 mg, 1 mmol) and the final solution is stirred for 30 min at room temperature. The solution was layered by Et_2_O, left undisturbed at room temperature. After two days, two distinct types of crystals formed. The target compound crystallized as yellow plates, while the blue block-like crystals were identified as Cu­(tmchd)­Cl_2_ by SCXRD, consistent with the previously reported structure.^40^ Anal. calcd. (found) for C_26_H_46_Cu_2_N_4_Cl_4_O_5_: 40.90 (43.58) %C, 7.34 (9.73) %N and 6.07 (9.89) %H. The elemental analysis deviates from the calculated values, consistent with the likely presence of byproducts other than Cu­(tmchd)­Cl_2_, as evidenced in the multiphase PXRD patterns. Target crystals were hand-picked for the single crystal X-ray diffraction and EPR measurements, but larger quantities for magnetometry, PXRD, and other bulk analyses were not possible. The UV–vis spectrum of the crude reaction mixture containing **6** was recorded (Figure S15) for qualitative comparison.

### [Cu_2_(4-Cl-terpy)_2_(μ-CA)]­(CF_3_SO_3_)_2_·H_2_O (7)

4′-Cl-terpy (0.5 mmol, 133 mg) was dissolved in 10 mL of hot MeCN. To this solution, a solution of Cu­(CF_3_SO_3_)_2_ (0.5 mmol, 180 mg) in 10 mL of MeCN was added. The resulting mixture was refluxed for 1 h, yielding a clear blue solution. Next, CA (0.25 mmol, 50 mg) was added as a solid in one portion. The solution immediately turned into a dark purplish-black suspension. The mixture was refluxed for another hour and then allowed to cool to room temperature. The suspension was filtered, and the resulting solution was layered with diethyl ether. After a few days, green plate-like crystals formed, along with crystals of Cu­(4′-Cl-terpy)­(CF_3_SO_3_)_2_ and a mononuclear compound with MeCN coordination along with a polymeric structure which were distinguished by single-crystal X-ray diffraction and visual inspection of crystal habit. All attempts to isolate sufficient crystals of compound **7** for bulk characterization were unsuccessful. Anal. calcd. (found) for C_38_H_22_Cu_2_N_60_Cl_4_F_6_S_2_O_11_·5H_2_O: 35.78 (35.55) %C, 6.59 (6.19) %N and 2.53 (1.87) %H. The UV–Vis spectrum of the crude reaction mixture containing **7** was recorded (Figure S16) for qualitative comparison.

### [Cu_2_(4-Br-terpy)_2_(μ-CA)]­(CF_3_SO_3_)_2_ (8)

Compound **8** was synthesized following a procedure similar to that of compound **7**, using 4′-Br-terpy (0.5 mmol, 150 mg), Cu­(CF_3_SO_3_)_2_ (0.5 mmol, 180 mg), and CA (0.25 mmol, 50 mg). After the addition of CA, the greenish-black suspension was refluxed for 1 h and then allowed to cool to room temperature. The suspension was filtered, and the resulting solution was layered with diethyl ether. After a few days, dark blue block-like crystals formed, together with crystals of [Cu­(4′-Br-terpy)­(MeCN)]­(CF_3_SO_3_)_2_ of similar color. Anal. Calcd. (Found) for C_38_H_20_Cu_2_N_6_Cl_2_F_6_S_2_Br_2_ O_10_ *·*10H_2_O: 31.77 (31.14) %C, 5.85 (5.35) %N and 2.81 (1.62) % H. As with compound **7**, attempts to isolate pure crystals of **8** for further characterizations were unsuccessful. The UV–Vis spectrum of the crude reaction mixture containing **8** was recorded (Figure S17) for qualitative comparison.

### [Cu_2_(tpa)_2_(μ-CA)]­(ClO_4_)_2_ (9)

The complex was synthesized by following the previous literature procedure,^38^ the only change was tpa as a ligand was used instead of tpa·3HClO_4_ precursor. The reaction was kept untouched overnight, and the next day, large dark blueish-purple single crystals suitable for X-ray diffraction were filtered off. (101 mg, 65% yield). Anal. calcd. (found) for C_42_H_36_Cu_2_N_8_Cl_4_O_12_ *·*1.5H_2_O: 44.19 (44.22) %C, 9.55 (9.82) %N and 3.44 (3.33) %H. UV–vis (DMSO, 1 mM, Figure S18) λ_max_ (nm) (ε_M_ (M^–^^1^ cm^–^^1^)): 327(43) and 521(62).

### X-ray Data Collection, Structure Solution and Refinement for 1–8 and 1a

Single crystal X-ray diffraction data were collected at the X-ray Diffraction Facility of The Ohio State University using a Bruker V8 Venture Diffractometer Mo-TXS equipped with Mo Kα radiation (λ = 0.71073 Å). Samples were cooled to 100 K with an Oxford Cryostream 700 low-temperature device. Data were collected and integrated with Bruker APEX3 software, and absorption corrections were applied using SADABS.^41^ For complex **8**, TWINABS-2012/1 (Bruker,2012) was used for absorption correction. Space groups were assigned on the basis of systematic absences, E-statistics, and successive difference refinements. Structures were solved with SHELXT and refined against successive Fourier maps using SHELXL in conjunction with OLEX2 software.^42–44^ Hydrogen atoms attached to carbon atoms were placed in idealized positions and refined using a riding model. For compounds **3**, **6**, and **7**, hydrogen atoms associated with water molecules were located from difference Fourier maps and refined with constrained isotropic displacement parameters. Data collection and refinement details are provided in the Supporting Information (Tables S1 and S9). Crystallographic information files for **1–8** and **1a** have been deposited with the CSD under accession numbers 2516526–2516530 and 2516929–2516932.

### Powder X-ray Diffraction

Powder X-ray diffraction (PXRD) patterns were collected on a Bruker D8 Advance diffractometer equipped with a Cu sealed tube and a LYNXEYE XE-T detector. The samples were finely ground, mounted on a zero-background sample holder, and measured at room temperature. Comparisons of PXRD patterns for complexes **1–7**, and **9** are provided in the Supporting Information (Figures S1–S7).

### Magnetic Measurements

Solid-state magnetic susceptibility measurements were obtained using a Quantum Design MPMS3 SQUID magnetometer (NanoSystems Laboratory, The Ohio State University). Samples were prepared by placing finely ground microcrystalline samples into a polycarbonate capsule. Direct-current (dc) magnetic susceptibility was measured in an applied magnetic field of 10,000 Oe (1 T) in the 2–300 K temperature range, with a temperature sweep rate of 5 K/min. The data were corrected for the diamagnetic contribution from the sample holder and for the intrinsic diamagnetism by using Pascal’s constants.^45^ The magnetic data were modeled using PHI,^46^ which uses the spin Hamiltonian convention *Ĥ* = −2*JŜ*_1_·*Ŝ*_2_. For comparison, use of the curry function in EasySpin^47^ reproduced the fits obtained from PHI. For consistency, all exchange interactions are reported as 2*J* corresponding directly to the singlet–triplet energy gap and providing direct comparison across the series and with EPR results.

### EPR Measurements

Continuous-wave (CW) X-band EPR spectra were collected using a Bruker EMXPlus equipped with a ColdEdge cryogen-free helium cryostat and recirculation system and an Oxford Instruments MercuryITC temperature controller, using a Continuous-wave (CW) perpendicular mode (ER 4119HS High Sensitivity Resonator). Parallel mode experiments were conducted using an ER 4116DM Dual Mode Resonator. All presented spectra were obtained using a microwave frequency of 9.37 GHz and a modulation amplitude of 10 G. EPR spectra collected herein were simulated using Easyspin 6.0.6 with the function Pepper (frozen solution and solid powder) and esfit functions; and were refined using simulations of the experimental data. For spectral simulations, the system is treated as an effects *S* = 1 state, and the parameters reported in Table 2 correspond to this total spin description. The effective *S* = 1 Hamiltonian is given by: *Ĥ* = μ*Bg*·*S* + *S*·*D*·*S* + *S*·*A*·*I* which includes the Zeeman, zero-field splitting, and hyperfine interactions modeled. Resulting values are reported in Table 2. Single crystals of **1–6** were manually selected and subsequently used to prepare powder and frozen-solution samples for EPR measurements All samples were prepared at atmospheric conditions as 1 mM solutions in butyronitrile (BuCN) for **1**, **1a**, **2**, **4–6**, ethanol (EtOH) for **3** or dimethyl sulfoxide (DMSO) for **9**. Solvent choice was guided primarily by compound solubility.

### Other Physical Measurements

UV–vis spectra were collected by using a Shimadzu UV-2600i instrument. IR spectra were recorded on a Bruker Ingenio-R FT-IR spectrometer. Data are provided in the Supporting Information (see the text in the ESI). Elemental analyses were performed by Atlantic Microlab (Norcross, Georgia, USA).

### Computational Details

To obtain insight into the electronic structure and magnetic properties of complex **1** and its mononuclear analogue **1a**, we performed multireference calculations using complete active space self-consistent field (CASSCF)^48^ and fully internally contracted second-order *n*-electron valence perturbation theory (NEVPT2).^49^ For each complex, two molecular geometries were employed in the calculations: one obtained from X-ray data and the other from structural optimization (see Tables S12–S14), which was performed using nonrelativistic state-averaged CASSCF (SA-CASSCF) including one singlet and one triplet state in a (2e, 2o) active space with the def2-SVP basis set.^50^

The energy levels and *g*-tensors of **1a**, **1**, and **1** without the ClO_4_^–^ counterion (1*) were computed using NEVPT2 with the DKH-def2-TZVP basis set.^51^ Density fitting^52^ was employed in both the NEVPT2 and SA-CASSCF calculations. BP86 ^53,54^ density functional theory (DFT) calculations with the DKH-def2-TZVP basis set provided initial orbitals for CASSCF orbital optimization. All states in the state-averaged calculations were assigned equal weights (see Tables S10 and S11).

The NEVPT2 calculations for the **1a** X-ray structure were performed using the (9e, 5o) active space with five reference doublet states. For **1** and 1*, calculations were carried out with three different active spaces. For the X-ray structure, the (10e, 9o) and (18e, 10o) active spaces were employed. For the optimized structures of **1** and 1*, the (10e, 10o) active space was used due to the convergence issues with the (10e, 9o) active space. In all NEVPT2 calculations, 24 triplet and 24 singlet states were incorporated in the SA-CASSCF reference (seeFigures S30–S39).

To account for relativistic effects, the Douglas-Kroll-Hess (DKH)^55^ Hamiltonian was employed to account for scalar relativistic effects, and the Breit-Pauli (BP) Hamiltonian^56^ was used to describe spin–orbit coupling effects. All NEVPT2 calculations were performed using the ORCA 6.0.1 software package.^57^ The *g*-tensors were computed using the SINGLE_ANISO module^58^ in ORCA.

## Results

### Syntheses

The syntheses of complexes **1–6** followed similar strategies to the reported procedure of **1**.^25^ Single crystals of the dinuclear species were obtained either by slow evaporation from MeOH (for **1**) or MeCN (for **4**), or by vapor diffusion of Et_2_O at room temperature (for **2, 3, 6–8**). Complex **9** was prepared according to the published method and crystallized analogously.^38^ Attempts to produce analogous dicopper­(II) chloranilate systems with Cl^–^ anions consistently resulted in complex product mixtures. For instance, efforts to prepare the Cl^–^ analogue **3** yielded three distinct crystalline species that could be identified in the final product: {[Cu_2_(Me_3_tacn)_2_(Cl)_2_(CA)]­(Cl)_*n*_}, Cu­(Me_3_tacn)­Cl_2_, and a small amount of the target complex **3**, illustrating the extreme sensitivity of this chemistry to subtle changes in crystallization conditions. Likewise, the 4′-Cl- and 4′-Br-terpyridine derivatives (**7** and **8**), analogous to the previously reported species with the observed singlet/triplet gap,^4^ produced inseparable mixtures of the desired dinuclear complex, a mononuclear Cu­(L)­(MeCN)_2_ species, and free-ligand byproducts. These examples highlight the inherent synthetic instability of earlier or highly substituted Cu­(II)–CA systems and underscore the value of the present Me_3_tacn and tmchd ligands, which reproducibly yield well-defined dinuclear products across multiple preparations. All complexes except **7** and **8** were isolated in moderate yields (>30%) and the crystals of the dinuclear species were stable in air for extended periods.

It is important to note that, in addition to the structures discussed here and shown in Figure 1, syntheses consistently produced a number of crystalline byproducts. Several of these byproducts correspond to mononuclear Cu­(II) complexes featuring different counterions and/or coordinated solvent molecules. This behavior is particularly pronounced for complexes **7** and **8**, where byproduct crystals are indistinguishable in both color and morphology from the targeted dinuclear species, significantly complicating their isolation, even by hand. In other cases, mainly for complexes **2–6**, byproducts include μ-hydroxo-bridged or polymeric Cu­(II) structures formed under similar reaction conditions. In all cases, PXRD was essential for evaluating the level of crystalline (im)­purity (see Figures S1–S8).

The experimental PXRD patterns broadly tracked with the simulated patterns calculated from the crystal structures, although discrepancies are observed across the dinuclear complexes (see Figures S1–S8). These discrepancies can arise from solvent loss (especially for **3**, **6**, and **7**), mixtures in the bulk powders, and minor contributions from mononuclear species, which were evident in several experimental patterns. Importantly, for complexes **1–5**, single crystals were hand-selected and afterward used to prepare the powder and frozen-solution samples for EPR measurements, thereby ensuring phase purity and preservation of the targeted dinuclear species. Taken together, the PXRD data highlight that bulk-phase purity, rather than single-crystal growth, is the greater of the two challenges in these systems.

### Crystal Structures

In this study, we cover nine different dinuclear complexes (Figure 2), seven of which are newly reported, and we classify them according to capping ligand: (i) Me_3_tacn, (ii) tmchd, and (iii) terpy/tpa. Table 1 highlights structural parameters relevant to magnetic exchange and spin Hamiltonian behavior, including Cu···Cu distances, axial ligand identity, Cu-ligand bond lengths, τ_5_ values, and metrics of the CA^2^-bridge. These factors ultimately shape the strength and sign of *J* and zero-field splitting in the triplet state. Additional crystallographic data and structural figures are provided in the ESI.

**Table 1:** Selected Structural and Magnetic Parameters for Complexes **1–** **9** ^a^

	*d*(Cu···Cu), Å	*d*(Cu–O_CA_), Å	Cu–O–C_CA_,°	*d*(Cu···X)^b^	τ_5_ ^c^	2*J*, cm^–1^ ^d^	*C* ^e^
**1**	7.689(3)	1.989(1)	113.94(4)	2.598(2)	0.027	–21.14(5)	0.38
**2**	7.681(4)	1.985(5)	113.88(1)	2.471(1)	0.025	–23.80(2)	0.48
**3**	7.777(2)	2.027(5)	113.18(5)	2.650(3)	n/a	–18.60(8)	0.32
**4**	7.660(2)	1.974(5)	113.81(2)	2.309(7)	0.082	–14.00(4)	0.08
**5**	7.691(2)	1.982(5)	114.04(7)	2.207(2)	0.094	–30.68(3)	0.68
**6**	7.712(3)	2.014(2)	112.66(3)	2.415(4)	0.114	–27.94(3)	0.60
**7**	8.071(3)	2.158(2)	115.30(3)	2.271(1)	n/a	n/a	n/a
**8**	8.072(2)	2.164(2)	115.32(3)	2.292(9)	n/a	n/a	n/a
**9**	8.285(2)	2.252(3)	117.03(5)	n/a	n/a	–1.26^f^	∼0

a All structures collected at 100 K. Subscript “CA” indicates atom that is part of chloranilate ligand.

b Distance from Cu to bound counterion donor atom, or to the O in H_2_O for **7** and **8.**

c Addison parameter describing five-coordinate geometry.

d Determined by best fit to the Bleaney–Bowers equation and a 2*J* Hamiltonian, where *J* is the strength of the magnetic exchange, and 2*J* corresponds to the singlet–triplet gap.

e Concurrence (*C*) values calculated at 8 K using the *J* constants of this table. See eq. 1.

f Taken from ref 38.

**Figure 2:**
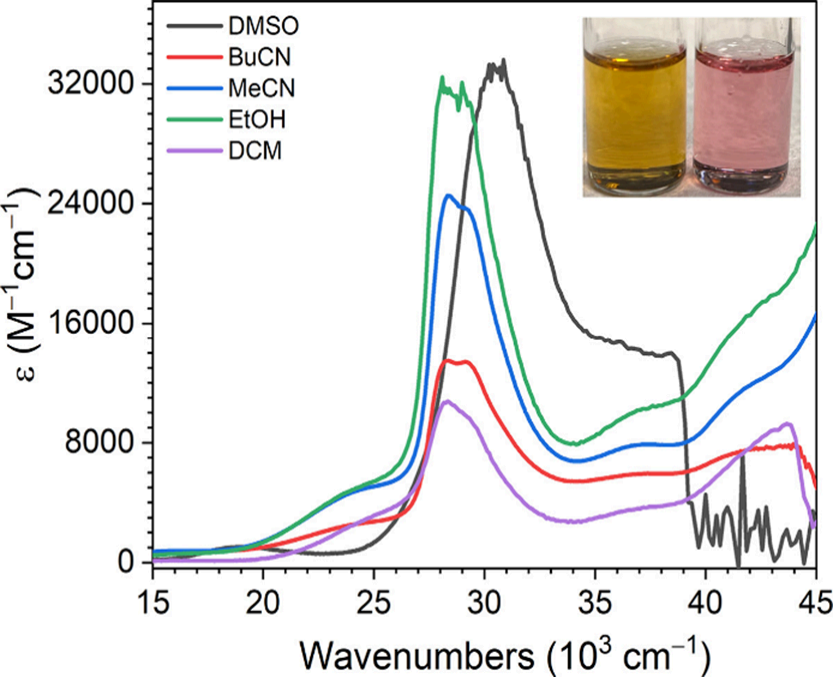
UV–Vis spectra of **1** recorded in various solvents. Inset: Photographs showing the color of **1** in different solvents, pink in DMSO and yellow in all others.

Complexes **1–3** feature square pyramidal-directing Me_3_tacn capping ligands. These complexes all share a planar chloroanilate (CA^2^^–^) bridge and three Me_3_tacn N-donors per Cu­(II) center. Cu–N bond lengths in the basal plane average ∼2.02 Å, while apical Cu–N or Cu–X distances range from 2.25 to 2.90 Å depending on bonding to the anion. The Cu···Cu distances are consistently short (∼7.68 Å), indicating minimal structural change with differing counterions. Complexes **1** and **2** exhibit weak or partial axial coordination by perchlorate (2.598(2) Å) or triflate (2.471(1) Å), respectively. In **3**, a bound axial Cl^–^ (2.650(3) Å) induces a more distorted octahedral geometry, where the Cu–Cl bond defines the Jahn–Teller elongation axis, increasing the Cu···Cu distance to 7.777(2) Å. The Addison parameters, τ_5_,^59^ range from 0.025 to 0.027 for **1** and **2**, indicating geometries closer to square pyramidal arrangements than trigonal bipyramidal ones. The mononuclear analogue Cu­(Me_3_tacn)­(CA) (**1a**) shows comparable bond metrics to these species.

The second series of complexes (**4–** **6**) feature the tmchd ligand as a square-planar-directing capping ligand for the Cu­(II) centers. As in **1–3**, chloroanilate again bridges the Cu­(II) ions by 7.66–7.71 Å. Complexes **4–6** also adopt distorted square-pyramidal geometries owing to weak interactions with the counterions, which exhibit somewhat longer Cu–X distances. For example, in **4**, axial coordination by perchlorate (*d*(Cu–O)_avg_ ≈ 2.30–2.33 Å) is notable, contrasting with the weaker Cu···ClO_4_^–^ interactions seen in **1–3**. In **6**, Cl^–^ occupies the apical site (Cu–Cl = 2.415(4) Å), with consistent Cu–N distances (∼2.01 Å) across both ions.

The final series of complexes (**7**-**9**) exhibit octahedral coordination environments imposed by multidentate N-donor ligands. In **7** and **8**, 4′-chloro- and 4′-bromo-terpyridine ligands coordinate each Cu­(II) center through three nitrogen atoms, while the chloroanilate bridge provides two equatorial O-donors per Cu. An additional axial ligand, H_2_O, is weakly bound in **7** and **8**. In **9** the Cu­(II) is coordinatively saturated by the tpa and CA ligands.

Jahn–Teller elongation is evident from the longer axial Cu–O distances (∼2.36–2.37 Å) compared to the equatorial Cu–O bonds (∼2.15 Å). The Cu···Cu separations (∼8.07 Å) are greater than those in square-pyramidal analogues, reflecting both axial elongation and the more extended ligand framework. These complexes are close structural analogues to the complex [Cu_2_(terpy)_2_(CA)]­(PF_6_)_2_ which was reported to exhibit observable singlet/triplet transitions.^4^ Complex **9**, supported by a tpa ligand, adopts a centrosymmetric geometry with Jahn–Teller elongation along the bridging axis, resulting in the longest Cu···Cu distance in the series (8.285(2) Å).

The role of counterions on the structures in the three sets of complexes appears to vary. For example, CF_3_SO_3_^–^ remains noncoordinating in **7** and **8**, as judged by long Cu–O distances (∼4.12 Å), and the same is true for ClO_4_^–^ in **9** with Cu–O distances of 6.17(3) Å. By contrast, CF_3_SO_3_^–^ coordinates more tightly in **2** and **5** with shorter distances: Cu–O = 2.471(1) and 2.207(2) Å, respectively. Chloride binds whenever it is present (**3** and **6**) but does so weakly (Cu–Cl = 2.650(3) and 2.415(4) Å).

Structural parameters of the chloroanilate (CA^2–^) bridging ligand are consistent with a quinoid resonance form, as observed in related systems.^4,25,60–66^ The central C=C bond measures 1.385(5) Å, significantly longer than in free chloroanilic acid (1.317 Å) indicating partial π-delocalization. The adjacent C–C bond (1.530(5) Å) closely matches that of the uncoordinated acid (1.512 Å), while C–O distances of 1.268(5) and 1.252(5) Å fall between typical single (1.320 Å) and double (1.225 Å) bonds, further supporting delocalization across the aromatic ring. The C–Cl bond length (1.722(4) Å) is consistent with reported values in ammonium chloroanilate^67^ and chloroanilic acid dihydrate.^68^ These metrics confirm that the CA^2–^ ligand retains a planar, conjugated structure across all complexes, reinforcing its role as a delocalized bridge that supports electronic and magnetic communication between the two Cu­(II) centers. The oxidation state and delocalized nature of the CA bridge are further supported by infrared spectroscopic measurements (see the FTIR discussion in the ESI).

### Electronic Spectroscopy

UV–Vis absorption spectra of complexes **1–6**, **9** and mononuclear analogue **1a** (Figure 3) were collected to investigate the solution-phase electronic structures. Measurements were performed in various solvents, including *n*-butyronitrile (BuCN), acetonitrile (MeCN), ethanol (EtOH), dichloromethane (DCM), and dimethyl sulfoxide (DMSO) (see Figures 2 and S9–S19). Because the solution-phase absorption spectra of compounds **7** and **8** largely mirror the mononuclear analogue **1a** (Figures S17 and S18), they will not be discussed in detail here. All complexes exhibit strong absorption bands above 30,000 cm^–^^1^ (<330 nm), assigned to ligand-centered π–π* or n−π* transitions,^69,70^ with modest solvent-dependent shifts in energy and intensity.^71–74^

**Figure 3:**
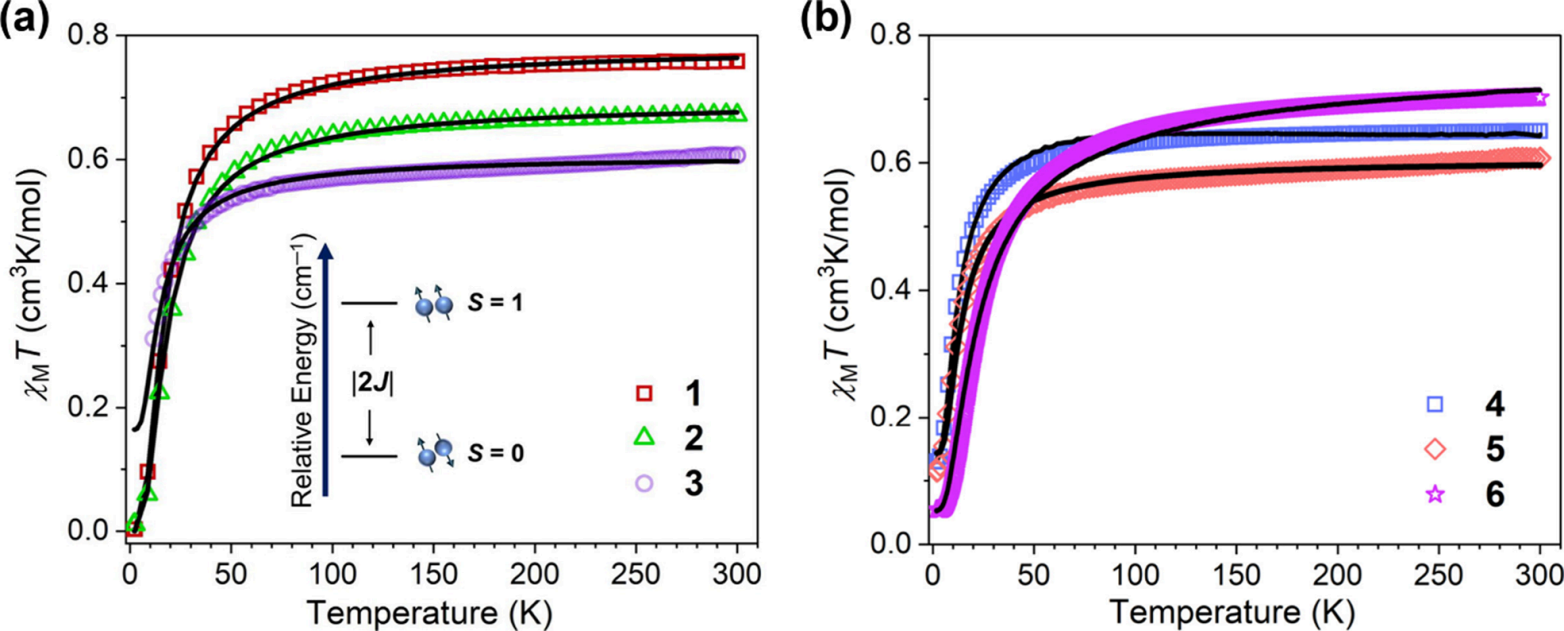
Temperature-dependent *χ*_M_ *T* values measured on microcrystalline (a) **1–3** and (b) **4–6** at an external field of 1 T; the solid lines are the best fits obtained with the parameters discussed in the main text.

In BuCN, MeCN, EtOH, and DCM, the dinuclear complexes form yellow to brownish solutions, and their spectra display two characteristic absorption regions: (i) intense ligand-centered bands at 28,800–30,000 cm^–^^1^ (333–347 nm) and (ii) broad, weak features at 16,300–16,900 cm^–1^ (592–614 nm), assigned to d–d transitions of square-pyramidal Cu­(II) centers with ε values < 300 M^–1^ cm^–1^, depending on solvent. Such low intensities are typical of Laporte-forbidden d–d transitions in Cu­(II) centers and are far below the ε values associated with charge-transfer bands (≥10^3^–10^4^ M^–1^ cm^–1^).^65^ By contrast, the higher-energy features above 22,000 cm^–1^ exhibit much larger intensities (ε ≈ 2–3 × 10^4^ M^–1^ cm^–1^) and pronounced solvent dependence, consistent with ligand-centered or ligand-to-metal charge-transfer (LMCT) transitions involving the chloroanilate bridge.^66^

These profiles all exhibit modest solvent-dependent shifts. For example, for **1**, the UV band moves from 29,400 cm^–^^1^ in DCM (340 nm) to 29,900 cm^–1^ in EtOH (334 nm), a difference of ≈500 cm^–1^, while the d–d feature varies by ≈300 cm^–^^1^ (16,500–16,800 cm^–1^). Compound **2** shows somewhat larger solvent effects, with the UV absorption spanning 28,800 cm^–^^1^ (DCM, 347 nm) to 30,000 cm^–1^ (DMSO, 333 nm), a range of ≈1200 cm^–1^, and the d–d transition shifting by ≈400 cm^–^^1^. By contrast, **5** exhibits comparatively minor variations, with the UV maximum moving by ≈500 cm^–^^1^ (29,300–29,800 cm^–1^) and the d–d band by ≈300 cm^–^^1^ (16,600–16,900 cm^–1^). Collectively, spectra recorded in weakly coordinating solvents (BuCN, MeCN, EtOH, DCM) remain nearly superimposable. Although **2** shows slightly larger solvent shifts than **1**, both complexes display similar solvent-dependence trends, confirming that counterion effects (ClO_4_^–^ vs CF_3_SO_3_^–^) are negligible compared to solvent polarity. The majority of the observed solvatochromism likely arises from weak solvent coordination or hydrogen-bonding interactions at the open axial site of the Cu­(II) center, which perturb both the LMCT and d–d transition energies. Similar solvent dependencies of CT and d–d bands have been widely documented for five-coordinate and pseudo-octahedral Cu­(II) complexes with accessible axial sites.^75–82^

In contrast to the dinuclear species, the mononuclear analogue **1a** forms pink solutions in BuCN. Its UV–vis spectrum features intense ligand-centered π–π* transitions at 29,500–31,000 cm^–1^ (∼250–340 nm) and a broad d-d absorption centered near 16,800 cm^–1^ (∼595 nm), consistent with a square-pyramidal Cu­(II) center with weak solvent interaction at the sixth coordination site.^83–86^ The well-defined d–d profile and lack of low-energy features confirm a single-site Cu­(II) center without magnetic coupling compared to complex **1**. These spectra establish **1a** as a quantitative reference for evaluating ligand-field strength and electronic delocalization in the dinuclear Cu_2_(μ-CA) complexes. Critically, dissolution of any of the dinuclear species in DMSO yields not a yellow-brown solution, but instead a pink one. In concert, the UV–Vis spectrum changes dramatically and closely resembles that of the mononuclear analogue **1a**.

### DC Magnetic Properties

Variable-temperature direct current (dc) magnetic susceptibility (*χ*_M_) measurements were collected on microcrystalline samples of **1–6** in the 2–300 K range under a 1 T applied (Figure 3). All complexes display behavior characteristic of antiferromagnetically coupled Cu­(II) ions. At 300 K, *χ*_M_ *T* values fall between 0.6 and 0.8 cm^3^K/mol, close to the spin-only limit for two uncoupled *S* = 1/2 centers (0.75 cm^3^K/mol). Upon cooling, *χ*_M_ *T* decreases in all cases, consistent with antiferromagnetic coupling leading to singlet ground states. Analyses of **7** and **8** were precluded by our ability to obtain phase-pure (or even mostly phase-pure) samples, as determined from PXRD and visual inspection of the samples.

The susceptibility data were modeled with the Bleaney–Bowers model to examine the strength of the magnetic coupling, *J*, using the spin Hamiltonian *Ĥ* = −2*JŜ*_1_·*Ŝ*_2_ for **1–6**. For all complexes, small values of paramagnetic impurities (≤5%) were necessary to successfully simulate the data. For all complexes, we report 2*J* values corresponding directly to the singlet–triplet energy gap, providing consistent comparison across the series and with EPR and computational results. Values of 2*J*, i.e., the singlet–triplet energy gaps are =–21.14(5), −23.80(2), and −18.60(8) cm^–1^ for 1–3, respectively, were extracted with *g* values that span from 1.99 to 2.08. For **4–6**, in contrast, fits with the Bleaney–Bowers model yielded 2*J* = −14.00(4) (**4**), −30.68(3) (**5**), and −27.94(3) cm^–1^ (**6**) with *g* ≈ 1.99–2.06. These values agree with similar chloranilate studies.^4,31,64,65,87^

### Concurrence and Spin Entanglement

Complexes **1–6** and **9** feature two *S* = 1/2 centers coupled through an antiferromagnetic exchange interaction (2*J* from −1.26 to −30.68 cm^–1^), yielding an entangled singlet (*S* = 0) ground state and a triplet (*S* = 1) manifold separated by approximately 2|*J*|. The higher energy triplet state contains three sublevels (*M*_S_ = 0, ±1) of which the *M*_S_ = 0 level is also entangled. The two *M*_S_ = +1 levels, in contrast, are not entangled.^88^

We sought to quantify how the spin entanglement varies in **1–6** and **9** with counterion and capping ligand. We did so by calculating the concurrence (*C*) of these complexes, which reflects the relative Boltzmann populations of the *S* = 0 and *M*_S_ = 0 entangled levels for a system. Here we qualitatively describe what this means. When interpreting the low-temperature *χ*_M_ *T* of an antiferromagnetic system, the low magnetic moment at low temperature signifies complete population of the *S* = 0 state.^89,90^ At higher temperature, a higher magnetic moment reflects population of the other *M*_S_ levels of the higher energy triplet. In terms of entanglement, the low-temperature *χ*_M_ *T* here reflects a high degree of entanglement with *C* = 1. In contrast, the higher-temperature *χ*_M_ *T* corresponds to populations of the nonentangled *M*_S_ = ±1 levels and signals a less-entangled, mixed-state through *C* < 1. Thus, *C* recontextualizes the outcome of the singlet–triplet energy gap in terms of an “entanglement strength” analogous to magnetic exchange interactions. For example, as a singlet/triplet gap increases and the singlet ground state becomes increasingly favored, the concurrence would argue that the entanglement in the system is higher, just as one would say the larger singlet/triplet gap reflects a stronger exchange coupling.

We applied a derived expression for *C* using the *χ*_M_ *T* data^90^ for **1–6** and **9** (Table 1):

$$C(T)=\max\left(0,\frac{\exp\left(\frac{3J}{4k_{B}T}\right)-3\exp\left(\frac{-J}{4k_{B}T}\right)}{\exp\left(\frac{3J}{4k_{B}T}\right)+\exp\left(\frac{-J}{4k_{B}T}\right)}\right)$$ 1

Here, *J* is still the exchange coupling (which corresponds to half of the experimentally reported 2*J* values in the DC magnetic properties section above), *k*_B_ is Boltzmann’s constant and *T* is the temperature for which *C* is calculated. Across the series, complexes with larger |*J*| (e.g., **5** and **6**) retain high concurrence at lower temperature (*C* ≈ 0.9 at 5 K), whereas systems with weaker *J* (e.g., **4**) exhibit lower concurrence (*C* ≈ 0.4) and a more rapid decay in *C* with increasing temperature.^91–93^ In aggregate, chemical modifications that influence *J*, such as changes in counterion or capping ligand, should provide a molecular handle for tuning spin entanglement in dicopper complexes. However, in **1–6** and **9** specifically, such changes produce only minor changes in *C*, indicating that the extent of spin entanglement is relatively robust to these chemical changes.

### Continuous-Wave (CW) EPR

X-band CW EPR spectra were collected on frozen solutions and microcrystalline powders of the complexes to identify how the counterion/capping ligand/environment influence the spin-Hamiltonian parameters of the studied compounds. Spectra were collected in a variety of common solvents, EtOH, DMSO, BuCN, and MeCN, with slight variations depending on complex solubility (Figures 4–6 and S20–S29). The spectra for **1–6** and **9** are dominated by an intense feature near 320 mT and additionally display a weak half-field transition near ca. 160 mT. For **1–3** frozen-solution spectra collected in different solvents display identical overall lineshapes, characterized by well-defined hyperfine structure. Complexes **4–6** present broader, less well-defined signals overall with reduced fine-structure features. The mononuclear analogue 1a exhibits a typical axial Cu­(II) line shape in all solvents, which is also consistent with the spectra observed for **7** and **8**. Notably, the dinuclear complexes generally display spectra in DMSO that all resemble the simpler axial pattern of **1a**. Complex **9** is distinct, however, and retains a half-field transition in DMSO. Finally, the spectra for **1** and **2** also reveal a variable-temperature intensity that increases with increasing temperature (Figure 5).

**Figure 4:**
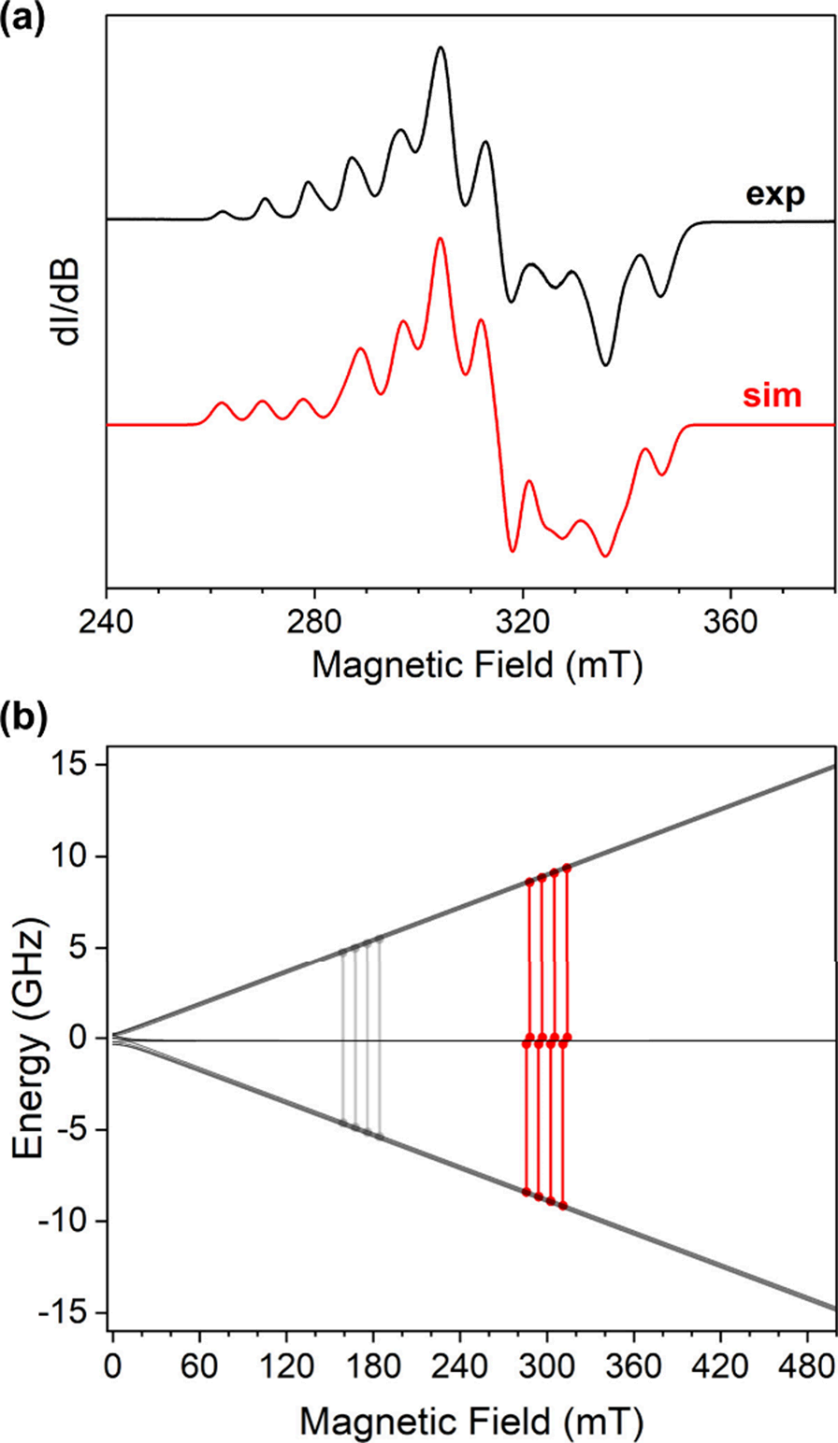
(a) X-band CW-EPR spectrum (black) of complex **1** recorded at 9.4 GHz and 8 K in a frozen BuCN solution (1 mM), overlaid with spectral simulation (red). (b) Zeeman energy level diagram as a function of magnetic field, highlighting allowed (red) and forbidden (gray) transitions. The observed resonance features are attributed to small zero-field splitting within the *S* = 1 spin manifold.

**Figure 5:**
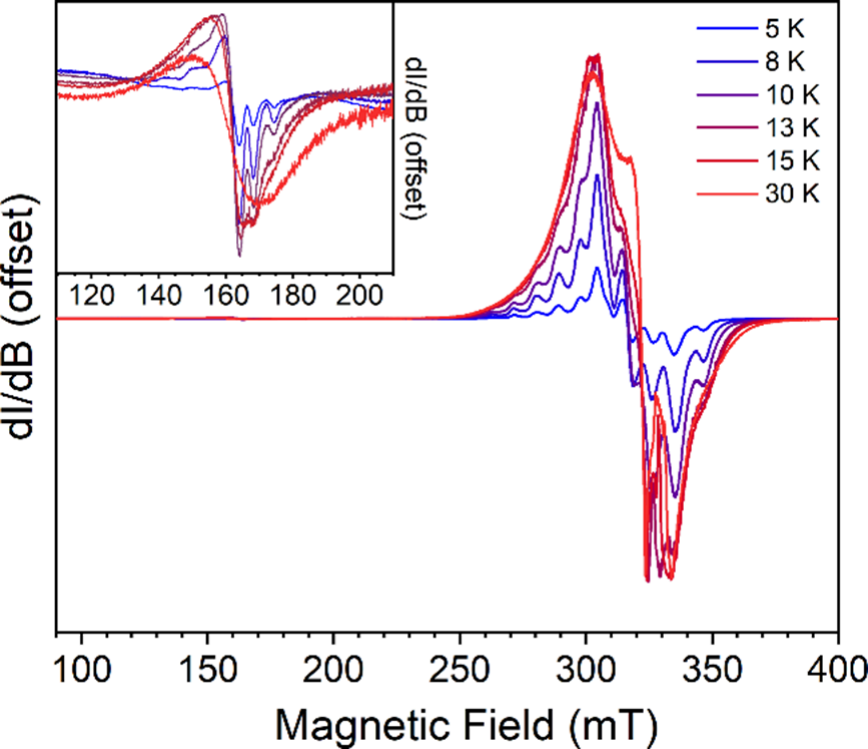
Variable-temperature X-band EPR spectra of the **1** in the solid state, recorded at temperatures ranging from 5 to 30 K. Full-field spectra showing the allowed signal at approximately 320 mT, with temperature-dependent variations in signal intensity and line shape. Inset: expanded low-field region highlighting the highly resolved features of the forbidden transition. The spectra exhibit broadening and shifting trends consistent with temperature-dependent spin interactions.

CW EPR spectra collected on powders generally reflect the solution-phase observations above. Across the series of complexes (**1–** **6**, **1a**, and **9**), the spectra consistently show a strong resonance near 320 mT and, in many cases, a weaker transition at ∼160 mT. Complexes **1** and **2** stand out for their exceptionally well-resolved powder spectra, which overlay closely with frozen-solution data in BuCN. In contrast, most other complexes (**3–** **6** and **9**) display broad, featureless powder spectra. This variability is not unusual for powder samples of dinuclear Cu­(II) complexes because of two factors. One, because these are powder rather than single-crystal spectra, anisotropy is inherently averaged, and when local disorder or distributions in exchange coupling dominate, the signals appear as broad unresolved peaks.^94,95^ Second, with increasing temperature (≥15 K), accelerated spin–lattice relaxation from the spin-concentrated powder further erases fine structure, leaving only broad peaks. Thus, while **1** (and partially **2**) provide unusually well-resolved powder spectra, the remainder behave as expected for exchange-coupled Cu­(II) systems.

We simulated many of these EPR spectra with Easyspin^47^ to extract the spin-Hamiltonian parameters (see Experimental) and investigate how they change with ligand/counterion selection. Parameters are summarized in Table 2. Note that susceptibility data suggest relatively well-isolated ground-state singlets for all dinuclear complexes at low temperature. Hence, an initial assumption could be that all dinuclear species should be EPR silent. However, any thermal population of the *S* = 1 state could yield an EPR signal, which we speculated was the origin of the increasing signal intensity with increasing temperature (Figure 5). Hence, all spectra were modeled assuming a triplet system. Best-fit simulations for **1–6** and **9** yielded *g*_∥_ = 2.20–2.23, *g*_⊥_ = 2.09–2.15, consistent with predominantly axial anisotropy, with variations in individual *g* values falling within experimental uncertainty. The fitted zero-field splitting parameters are small (|*D*| = 147–210 and *E* = 12–26 MHz) and comparable to prior reports for dinuclear copper­(II) complexes.^4,60,65,87^ An estimate of the dipolar contribution based on the crystallographic Cu···Cu distances^28^ gave values on the order of ∼137–171 MHz. These values are comparable to the experimentally determined *D* parameters, indicating that dipolar coupling accounts for a substantial fraction of the axial zero-field splitting.

**Table 2:** Summary of Spin-Hamiltonian Parameters for Dinuclear Complexes **1–6**, and **1a**

complex	*g*_avg_	*g*_*z*_ ^b^	*g*_*x*_ ^b^	*g*_*y*_ ^b^	|*D*|^b^ ^c^	*E^b^ ^c^*	*A* *z* ^b^ ^c^	*A* *x* ^b^ ^c^	*A*_*y*_ ^b^ ^c^	*p* ^a^	lwwp
**1**	2.06(1)	2.23(1)	2.09(2)	2.12(4)	210(5)	18(8)	120(4)	54(5)	99(8)	0.008(4)	1.7023
**1a**		2.19(2)	2.09(4)				252(5)	15(2)			2.10
**2**	1.99(3)	2.21(5)	2.12(3)	2.11(3)	147(9)	12(2)	253(9)	89(6)	63(7)	0.31(5)	1.8501
**3**	2.08(5)	2.25(4)	2.13(3)	2.11(2)	168(2)	13(3)	209(7)	88(6)	78(6)	0.35(4)	2.0683
**4**	2.06(2)	2.23(4)^8^	2.11(3)^7^	2.13(4)^8^	159(7)^1^	21(3)	160(8)	38(4)	100(6)	0.34(6)	2.5319
**5**	1.99(1)	2.21(5)	2.07(2)	2.11(5)	194(6)	18(2)	143(5)	31(4)	49(2)	0.22(2)	2.7820
**6**	2.00(2)	2.22(6)	2.11(9)^7^	2.12(2)^1^	150(4)	20(3)	163(5)	50(6)	80(8)	0.28(5)	2.6760
**9**	2.19	2.20(4)	2.15(3)	2.11(2)	154(1)	26(5)	174(4)	39(3)	40(2)		3.0451

a Obtained from fits of magnetic susceptibility data.

b Obtained from simulations of the 8 K frozen-solution EPR spectra. *p* is fractional contribution of impurity and lwwp is the peak-to-peak line width used in the simulations.

c In units of MHz.

Similar to the *g* values, *D* and *E* values show minimal variation across the complexes and no overt dependence on capping ligand or counterion was observed. Instances where this simulation strategy proved ineffective occurred when the spectra were far simpler and closer to expectations for mononuclear species. We note that the zero-field splitting parameter *D* is reported here as its magnitude, |*D*|, since the sign cannot be reliably determined from conventional X-band EPR measurements, where resonance positions are insensitive to the sign. Determination of the sign requires high-field EPR experiments and analysis of transition intensities, typically on oriented single crystals, particularly for antiferromagnetically coupled systems.^96^ Consistent with this, inversion of the sign of *D* does not alter the quality of the spectral simulations, and therefore only |*D*| is discussed. Early studies reporting singlet–triplet (Δ*M*_S_ = ±2) transitions in dinuclear systems did not explicitly determine or report either the magnitude or sign of *D* under comparable conditions.^4,31,97–100^ In the present work, qualitative comparisons are instead made with more recent dinuclear copper­(II) systems exhibiting similar exchange coupling (*J*) values.

The effective simulations allow for a comprehensive interpretation of the continuous wave spectra. For one, across the series of complexes (**1–6** and **9**), the spectra consistently show a strong Δ*M*_s_ = ±1 resonance near 320 mT which can be assigned to the triplet manifold that has appreciable thermal population. This interpretation explains the increasing signal intensity with increasing temperature, as more of the sample is populating the EPR-active triplet at higher temperature. It also explains some of the trends in the observed broadening: at low temperature, the majority of the dinuclear species occupy the *S* = 0 state. Hence, the environment is effectively diamagnetic and we see the result: sharpening of the spectrum in the solid akin to the spectra of the dilute frozen solutions. Second, the model also explains the resonance at half the magnetic field of the main resonance as a forbidden Δ*M*_S_ = ±2 transition. We tested this hypothesis by applying parallel-mode measurements to **1** as a test case. If the assignment of the transition were correct, then the parallel-mode spectrum should amplify and sharpen the transition. Indeed, parallel-mode spectra of **1** collected at 8 K (on powder and 1 mM BuCN solution, Figure 6) reveal a signal that is sharper than observed in the perpendicular mode spectra. Hence, we conclude that this transition is indeed the forbidden Δ*M*_S_ = ±2 one within the triplet manifold.

**Figure 6:**
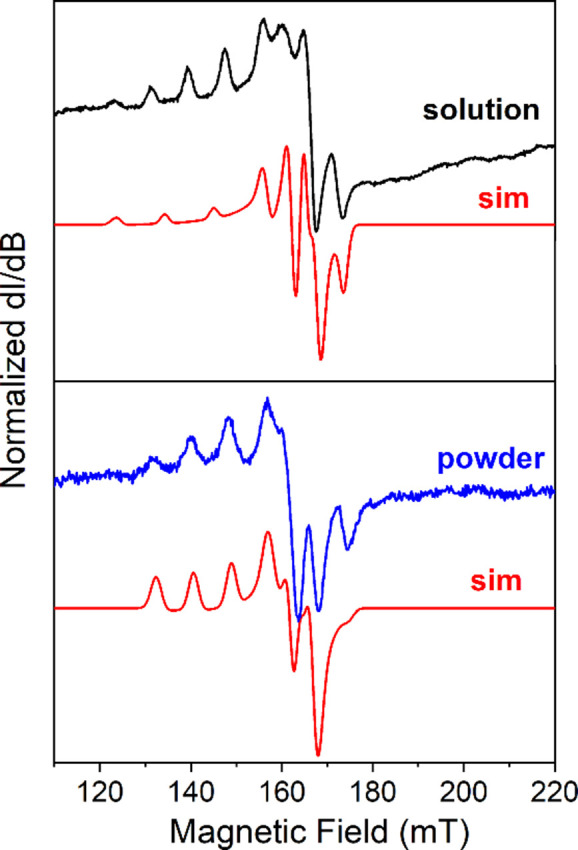
X-band parallel-mode EPR spectra recorded at 8 K for 1 under different sample preparation conditions: as a powder (blue), in frozen glass BuCN at 1 mM (black).

Best-fit simulations of the parallel-mode spectra generally follow the observed trends for perpendicular mode measurement. The best simulation for **1** (Figure 6) yielded axial *g*-tensors: *g*_∥_ = 2.37 and 2.48 and *g*_⊥_ = 2.18 and 2.17 for the powder and solution samples, respectively, and the corresponding zero-field splitting parameters and |*D*| = 84 and 96 MHz for powder and solution, respectively. The hyperfine coupling constants span 36–179 MHz (powder) and 19–240 MHz (frozen solution). Simulations produced *A*_∥_ = 179 (powder) and 240 MHz (frozen solution) with *A*_⊥_ = 36 and 118 MHz (powder) and 19 and 112 MHz (frozen solution). The large difference between *A*_∥_ and *A*_⊥_ indicates stronger anisotropy than accounted for in perpendicular-mode simulations. The close agreement between powder and solution parameters supports retention of the dinuclear structure in solution. However, there are slight differences in the parameters extracted from the two simulations, which allows for a natural estimate of the quantitative reliability of the two data parameter sets.

Finally, we note that the thermal dependence of the EPR intensity should reflect the Boltzmann populations of the triplet, just like *χ*_M_ *T*. The temperature-dependent EPR intensity for compound 1 (Figure S30) was fitted with a Boltzmann expression based on the Bleaney–Bowers formalism (*Ĥ* *=* −2*J* **S**_1_ *·* **S**_2_) which yielded *J* ≈ −6 cm^–1^ (Δ*E* ≈ 12 cm^–1^). The smaller magnitude relative to magnetometry is likely from line-width effects that come with using peak-to-peak intensities. A similar analysis can be applied to the Δ*M*_S_ = ±2 transition, but the significantly lower intensity complicates a direct quantitative treatment. Nevertheless, we observe reasonable agreement, and the same arguments about concurrence that were applied to *χ*_M_ *T* can extend over to the discussion of the EPR intensity as well.

### Computational Modeling of the Magnetic Interactions

A deeper understanding of spin exchange and spectroscopic selection rules in dinuclear copper­(II) complexes requires insights from a theoretical framework that directly connects the magnetic parameters of the triplet state (namely, the exchange coupling *J*, the zero-field splitting |*D*|, and the *g-*tensor) to their underlying electronic structure. Developing such a picture is particularly challenging for large, chemically complex species,^101–104^ such as complexes **1–** **9** with many atoms and intricate frontier-orbital manifolds.

To obtain a comprehensive description of the molecular magnetism, we employed multireference N-electron valence second-order perturbation theory (NEVPT2) with accurate treatment of electron correlation and spin–orbit coupling effects, using complex **1** as a prototype. A central goal was to assess whether this methodology can reproduce (1) the experimentally observed singlet–triplet gap, (2) the zero-field splitting in the triplet state, and (3) the anisotropic *g*-tensor, while providing insight into the orientation of the *g*-tensor principal axes essential to interpreting spectra.

Consistent with experiment, the NEVPT2 calculations predict that complex **1** has a singlet (*S* = 0) ground state with two antiferromagnetically coupled Cu­(II) atoms, each in a d^9^ configuration. At the X-ray crystallographic geometry, this ground-state assignment is unchanged upon removal of the ClO_4_^–^ counterion: for a fixed molecular structure, the presence or absence of the counterion has a negligible effect on the singlet–triplet energy gap and on the qualitative electronic character of the low-lying states (Table 3). Geometry optimization of **1** without ClO_4_^–^ (1*) introduces only modest changes to the Cu–N bond distances and coordination angles, and the singlet remains the ground state in all cases. We therefore focus the following analysis on 1*.

**Table 3:** Calculated Energies (*E*, cm^–1^) and Principal *g* Values of 1* ^a^

	(18e,10o)		(10e,9o)
active space	optimized structure	X-ray structure	X-ray structure
*E*(*S* = 0)	0.00	0.00	0.00
*E*(*S* = 1)	2.05	2.12	9.96
	2.09	2.13	9.96
	2.10	2.18	9.96
*g*_*z*_	2.50	2.40	2.03
*g*_*x*_	2.10	2.07	2.00
*g*_*y*_	2.11	2.09	2.00

a All energies are relative to the *S* = 0 level. Energies were calculated using NEVPT2 with 24 singlet 24 triplet states and the DKH-def2-TZVP basis set. The complete data, including higher excited state energies, are shown in ESI.

The NEVPT2 calculations predict that the first excited state of 1* is a triplet (*S* = 1) that lies just a few wavenumbers above the singlet ground state. The singlet–triplet energy gap, expressed through the exchange coupling constant *J*, shows some sensitivity to the molecular geometry (X-ray versus optimized) and to the technical details of the NEVPT2 calculations, namely the active-space size and character, the number of electronic states, and the basis set. Across all geometries and NEVPT2 parameters explored, the |2*J*| coupling (|2*J*| *= E*(*S* = 1) – *E*(*S* = 0)) computed using the DKH-def2-TZVP basis set^51^ falls in the 2–10 cm^–1^ range, systematically underestimating the experimental |2*J*| = 21.1 cm^–1^ but correctly capturing a small, antiferromagnetic exchange. In contrast, the zero-field splitting of the triplet is predicted to be extremely weak, with *D* values between ∼0.002 to 0.05 cm^–1^, in a good agreement with the experimental *D* = 0.006 cm^–1^ (Table 2).

The origin of the small *D* parameters can be traced to the frontier electronic structure (Figures S31–S39). Figure 7 displays the frontier natural orbitals of 1* for the *S* = 0 and *S* = 1 states computed using complete active space self-consistent field (CASSCF) with 10 electrons in 9 active orbitals ((10e, 9o)), which include the d_*x* ^2^–*y* ^2^_ and d_*z* ^2^_ atomic orbitals of Cu and the π-orbitals of CA ligand (Figure S40). In both spin states, the two singly occupied natural orbitals are well described as in-phase and out-of-phase linear combinations of the Cu d_*x* ^2^–*y* ^2^_ atomic orbitals. Such electronic structure gives rise to a triplet state with very little unquenched orbital angular momentum, explaining very small zero-field splitting observed experimentally and reproduced in the calculations. Indeed, this result corroborates the dipolar analysis we applied earlier in the manuscript.

**Figure 7:**
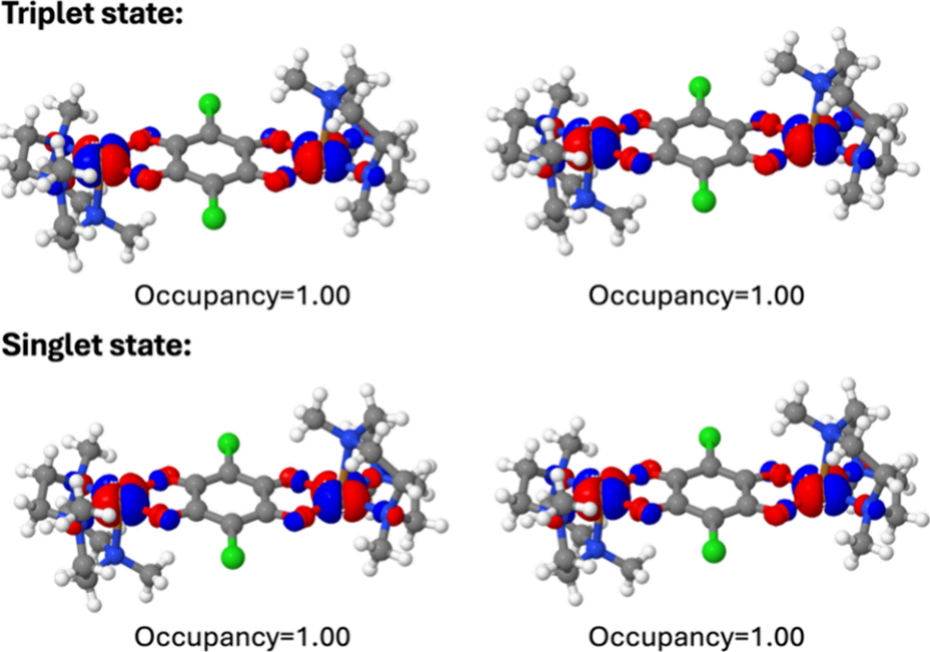
Natural frontier orbitals of complex 1* obtained from (10e,9o) CASSCF calculations for the X-ray crystal structure.

Next, we analyzed the *g-*tensor of triplet 1* to probe magnetic anisotropy in more detail. The NEVPT2 calculations incorporating spin–orbit coupling effects qualitatively reproduce the experimental anisotropy pattern, with *g*_*z*_ > *g*_*x*_ ≈ *g*_*y*_ (Table 3), but the computed *g*-values are strongly dependent on the active space and on the number of electronic states. At the X-ray geometry, the *g* values computed using the (10e, 9o) active space with 24 singlet and triplet states are significantly underestimated (*g*_*x*_ = 2.00, *g*_*y*_ = 2.00, *g*_*z*_ = 2.03) relative to the results of EPR measurements (*g*_*x*_ = 2.10, *g*_*y*_ = 2.10, *g*_*z*_ = 2.23, Table 2). Using the (18e, 10o) active space that includes all Cu d orbitals, markedly improves the agreement with experiment (*g*_*x*_ = 2.07, *g*_*y*_ = 2.09, *g*_*z*_ = 2.40). Optimizing the molecular geometry improves the agreement for *g*_*x*_ and *g*_*y*_ but yields a more overestimated *g*_*z*_ (*g*_*x*_ = 2.10, *g*_*y*_ = 2.11, *g*_*z*_ = 2.50).

These results can be rationalized using ligand field theory.^105^ The shift in *g*_α_ (Δ*g*_α_, α = *x, y, z*) relative to that of a free electron (*g*_e_ ≈ 2.0023) is proportional to the spin–orbit-mediated coupling between d orbitals via the matrix elements of the angular-momentum operator along the α axis (*L*_α_). In a square-pyramidal environment of each Cu­(II) center in 1*, the Δ*g*_*x*_/Δ*g*_*y*_ shifts arise from the *L*_*x*_/*L*_*y*_-coupling between the d_*x* ^2^–*y* ^2^_ and d_*yz*_/d_*xz*_ orbitals, whereas Δ*g*_*z*_ is controlled by the *L*_*z*_-coupling between d_*x* ^2^–*y* ^2^_ and d_*xy*_.^106^ The corresponding matrix elements, ⟨d_*xy*_|*L̂*_*z*_|d_*x* ^2^–*y* ^2^_⟩ = 2*i* and ⟨d_*yz*_|*L̂*_*x*_|d_*x* ^2^–*y* ^2^_⟩ *=* ⟨d_*xz*_|*L̂*_*y*_|d_*x* ^2^–*y* ^2^_⟩ = −*i*, predict a larger Δ*g*_*z*_ than Δ*g*_*x*_ and Δ*g*_*y*_, consistent with the experimentally observed anisotropy (Δ*g*_*z*_ ≈ 2Δ*g*_*x*_, 2Δ*g*_*y*_). Further insight into the magnitude of these shifts follows from the sum-overstates expression for the molecular *g*-tensor,^107,108^ which shows that nonzero Δ*g*_α_ contributions arise from electronic configurations involving excitations out of d_*xz*_, d_*yz*_, and d_*xy*_ orbitals into higher-lying molecular orbitals. Accurate prediction of the *g-*tensor therefore requires a correlated treatment that includes all Cu d orbitals together with a sufficiently large manifold of excited states, explaining the pronounced active-space dependence of the NEVPT2 results.

Figure 8 depicts the principal *g*-tensor axes of 1* computed using the (18e, 10o) active space at both the X-ray crystal and optimized geometries, alongside the *g*-axis orientation of the mononuclear complex **1a** computed with a (9e, 5o) active space. In all three structures, the principal *g*_*z*_ axis is approximately perpendicular to the CA ligand plane and is only weakly affected by dimerization or geometry relaxation, indicating that the dominant anisotropy is set by the local square-pyramidal ligand field at each Cu center. The *g*_*x*_ and *g*_*y*_ axes of 1* lie largely within the CA plane and closely follow those of **1a**, with only minor tilts upon going from the X-ray to the optimized geometry. These observations show that the weak exchange coupling in 1* has a small effect on the magnitude of the *g* anisotropy relative to **1a** and does not significantly affect the magnetic axes, implying that the EPR response is governed mainly by local ligand-field tuning rather than by large-scale structural distortions. This robustness of the *g*-frame also suggests that modifications to the equatorial ligand environment can be used to systematically adjust the magnitude of the anisotropy while maintaining a well-defined orientation of the magnetic axes relative to the molecular scaffold and applied field.

**Figure 8:**
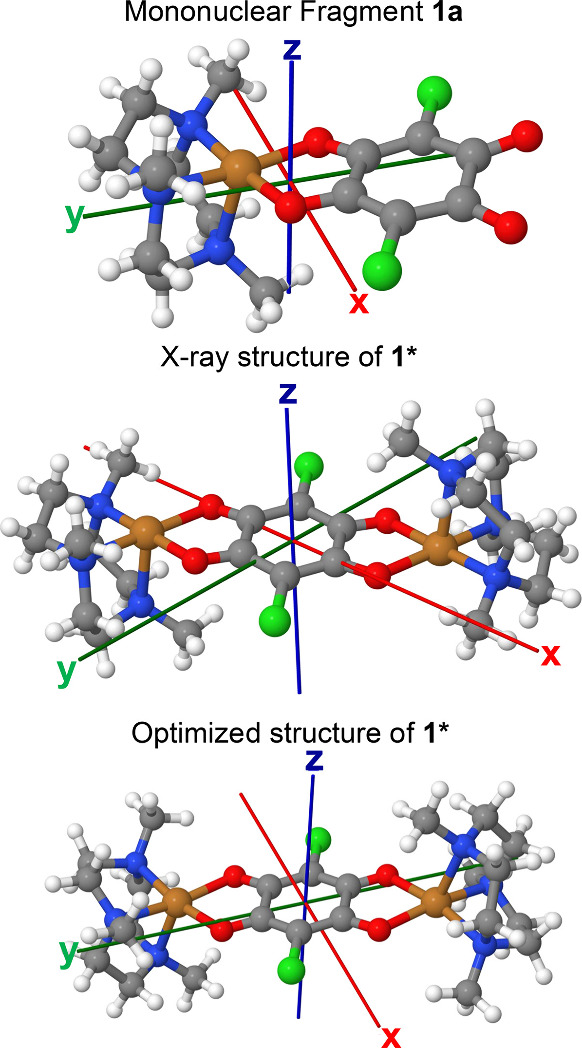
The *g*-tensor orientations of **1a** and 1* (from experimental XRD data and following optimization) computed with the (9e, 5o) and (18e,10o) active spaces, respectively. The origin of all axes is the center of charge for the ions/molecules. No significant change in parameters was observed when shifting the origin to the Cu atom for **1a**.

In summary, the NEVPT2 analysis of **1** provides detailed insights into how its magnetic properties emerge from the underlying Cu­(II) electronic structure. It shows that the weak antiferromagnetic exchange and nearly vanishing zero-field splitting originate from a pair of predominantly d_*x* ^2^–*y* ^2^_-based singly occupied orbitals with little residual orbital angular momentum, while the strongly axial *g*-tensor reflects ligand-field-controlled spin–orbit coupling between d_*x* ^2^–*y* ^2^_ and the remaining d orbitals. The sensitivity of the calculated *g*-values to the active space demonstrates that a realistic description of the anisotropy requires explicit inclusion of all Cu d orbitals and a sufficiently large manifold of excited states. Finally, the nearly unchanged *g*-axis orientations across **1**, 1*, and **1a** indicate that the local square-pyramidal ligand field fixes the magnetic frame, implying that equatorial ligand modifications can be used to tune the magnitudes of *J*, *D*, and *g*-anisotropy while preserving a well-defined orientation of the magnetic axes.

## Discussion

The foregoing spectroscopic, magnetometric, and computational results have significance in interpreting the stabilities, magneto-structural correlations, and finally, understanding the variation in the EPR spectroscopic properties of the compounds, which are discussed in that order.

### Solution-Phase Stability of the Cu_2_ Complexes

The structural integrity of the [Cu_2_(L)_2_(μ-CA)]^2+^ cores are effectively assessed by the aggregate of our UV–vis and low-temperature cw EPR spectral data. As a starting point for reaching this conclusion, we hypothesized that complex integrity in solution should reveal consistent magnetic parameters and electronic structures between solid and solution measurements. Depending on structural integrity, we might even expect stark similarities between spectra collected in different solvents and anion choices.

The UV–vis spectra of **1–** **6** and **9** display generally high consistency in weakly coordinating solvents but differ sharply in DMSO. Furthermore, in DMSO, the UV–vis spectra for **1–** **6** look notably similar to **1a**. These observations suggest to us that the dinuclear Cu_2_(μ-CA) core is stable to relatively weakly coordinating solvents, but not to DMSO; instead, dissociating into mononuclear Cu­(II) species in which DMSO and/or displaced ligand fragments coordinate to copper. We propose that this DMSO-sensitivity may be exclusive to the ligand shells that offer open sites for DMSO coordination.

Low-temperature CW-EPR gives further information about potential stability. Here, frozen-glass spectra largely reproduce the key features of the powder spectra, including *g* values, ZFS parameters, and the Δ*M*_S_ = ±2 forbidden transitions. Fragmentation of these species in solution should remove many of these signals, instead producing EPR spectra that resemble **1a**. The forbidden transition is a key signal here, as that can only result from a dinuclear system and its thermally populated *S* = 1 state preserved under vitrification. Some spectra, e.g., MeCN, show slightly reduced resolution, consistent with less effective glass formation by the solvent, but otherwise retain these dinuclear signatures. By contrast, DMSO solutions of **1**, **1a**, and a Cu­(ClO_4_)_2_/Me_3_tacn control yield broad, weak signals and no Δ*M*_S_ = ±2 transition.

Taken together, we interpret these UV–vis and EPR results to indicate that **1–6** are electronically and magnetically robust in non and weakly coordinating solvents but undergo disruption in strongly coordinating media such as DMSO. As DMSO can sometimes be needed to increase solubility for frozen glasses,^99,109–112^ especially for charged metal complexes, these studies suggest that future explorations of spin entanglement should find other options for frozen glass preparation. Indeed, the robustness of the molecules in BuCN solution is promising owing to the ease of use for this glassing solvent. An exception is **9**. This complex still shows the Δ*M*_S_ = ±2 transition even in frozen DMSO (Figure S29). We propose that this system may have a higher solution stability from the tpa ligand, which we propose is blocking DMSO coordination and preventing cluster fragmentation.

### Magneto-Structural Analyses

Across complexes **1–6**, there are two sets of magnetic parameters of explicit interest to us. First, the magnitude of the exchange interactions, *J*, (or 2*J* values, which correspond to the singlet to triplet energy gap) as this interaction defines many important aspects of the systems, including the concurrence, *C* at a given temperature/field. The *J* also determines the potential field/frequency conditions under which certain forbidden transitions may be observed (Figure 9). Second, the explicit *A*, *g*, *D*, and *E* parameters of the compounds are important, as these have measurable practical outcomes on what signals can be observed, when they are broad/sharp, etc. Our successful modeling of the susceptibility and EPR data with antiferromagnetically coupled dicopper system permits us to make some broader conclusions about the relationships between the molecular structures and the above magnetic parameters.

**Figure 9:**
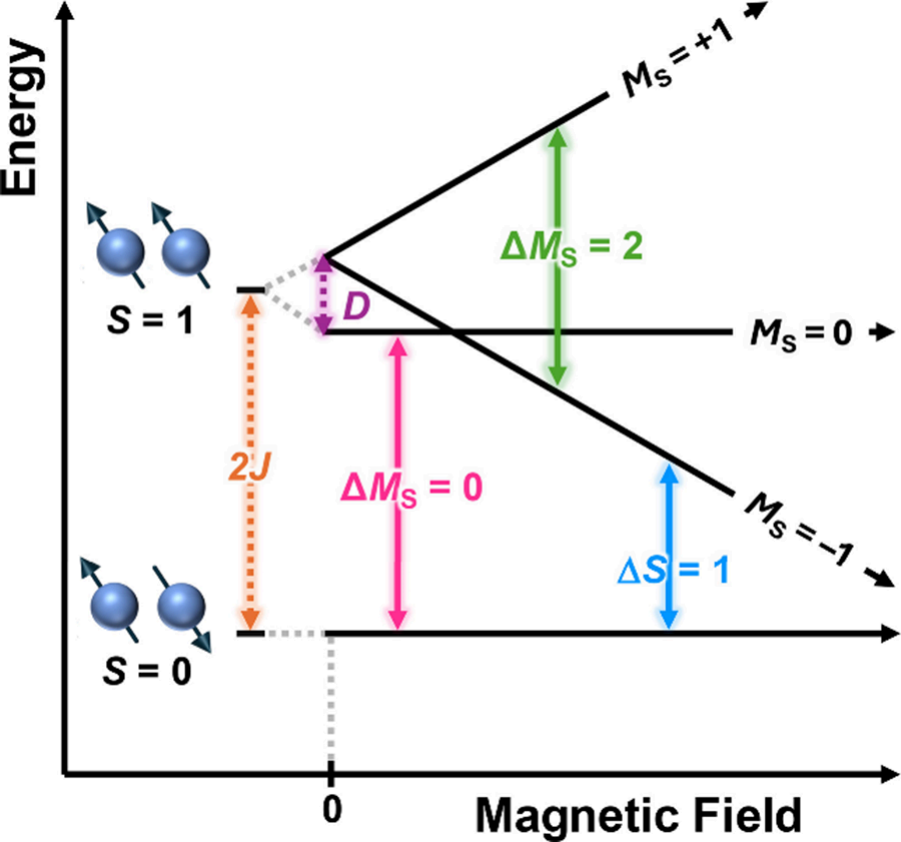
**Energy**-level diagram for an exchange-coupled dinuclear Cu­(II) complex with emphasis on showing the forbidden transitions. The diagram displays the singlet–triplet splitting gap (2*J*, orange) and zero-field splitting (*D*, purple) of the triplet manifold. The three types of forbidden resonances are the zero-quantum transition between the *S* = 0 and *M*_S_ = 0 level of the triplet (magenta), the “half-field” transition of Δ*M*_S_ = ±2 for the triplet (green), and the single-triplet Δ*S* = 1 transition (blue).

First, we observe a somewhat suppressed sensitivity of the exchange coupling to changes in the capping ligands. We initially anticipated that the magnetic exchange parameter would vary strongly with the selected capping ligand. The magnitude of *J* depends on both the Cu···Cu separation and, more importantly, the orbital overlap between the Cu­(d_*x* ^2^–*y* ^2^_) magnetic orbitals and the π system of the bridging chloranilate ligand, which in principle is strongly dictated by the local coordination environment. Here it seems like the capping ligand, in a way, governs how strongly the counterion has an influence on *J*. For example, the Me_3_tacn series (**1–3**) exhibits a modest variation in *J*, with ClO_4_^–^ and CF_3_SO_3_^–^ counterions giving nearly identical exchange constants (−12 and −11 cm^–1^) indicative of limited counterion changes to the Cu­(II). The tmchd series (**4–6**), in contrast, shows a broader spread of *J* values. Complexes 4–6 have consistently shorter Cu···X distances than **1–3**. In this case, the bidentate nature of tmchd appears to be important, as Me_3_tacn only leaves an open site that coincides with the elongated Jahn–Teller axis. Hence, counterion changes have a larger effect on Cu­(II)-ligand orbital overlap, and hence *J*. Finally, **9** represents the limiting case, exhibiting very weak antiferromagnetic coupling (*J* ≈ −0.6 cm^–1^), where Jahn–Teller elongation reduces effective overlap with the bridge and effectively suppresses superexchange. Overall, however, the counterion and ligand substitutions produce remarkably minor changes in *J*, unless axial coordination directly perturbs the superexchange pathway. These same observations extend to descriptions of entanglement defined by *C.*

Second, the simulations of the EPR data allow us to discuss the possibility of finding magnetostructural correlations in the context of forbidden transitions. We first discuss the spin Hamiltonian parameters revealed by EPR, then the allowed v. forbidden transitions within the triplet manifold, and finally the potential for transitions between the singlet/triplet. Figure 9 provides a graphical overview of all possible transitions within the spin manifold of the species studied here.

Across complexes **1–6** and **9**, the spin Hamiltonian parameters (aside from *J*) are remarkably conserved despite changes in counterion and supporting ligand environment. All compounds exhibit predominantly axial *g* tensors (*g*_∥_ ≈ 2.20–2.25; *g*_⊥_ ≈ 2.07–2.15), consistent with Cu­(II) d_*x* ^2^–*y* ^2^_ ground states.^4,113^ The zero-field splitting parameters span a relatively narrow range (|*D*| ≈ 147–210 MHz; *E* ≈ 13–26 MHz) with no systematic dependence on counterion or ligand identity. Hyperfine couplings likewise show only modest variation (*A*_*z*_ ≈ 80–210 MHz with smaller *A*_*x*_/*A*_*y*_ components), and among all complexes, *D*, *E*, and *A* display no clear correlations with counterion substitution. We propose that all of these data evidence a primary importance of the binding ligand on the overall magnetic properties of the material. Indeed, across all complexes, the Cu···Cu distances and relative orientations of the Jahn–Teller axes are generally consistent, which further agrees with an origin in *D* that depends primarily on dipolar interactions.

Within the triplet manifold, the interplay of the axial (*D*) and transverse (*E*) anisotropies appear to influence the relative intensities of the forbidden Δ*M*_S_ = ±2 transition. For example, **1**, which retains the highest axial symmetry, exhibits the largest zero-field splitting (*D*), the smallest rhombicity (*E*/*D*), and the narrowest linewidths, yielding the most intense half-field signal. In contrast, **2** and **3** display similarly small *D* values but increased rhombicity, which broaden the spectra and suppress forbidden features, and indeed the Δ*M*_S_ = ±2 transition is weaker for these two. Complexes **4–6** exhibited broader, less structured spectra, with reduced half-field intensity. Extracted |*D*| values are smaller, and the *E* values are larger compared to **1–3**. The hyperfine coupling tensors vary broadly in **1–3**, while **4–6** display more uniform and generally smaller *A* components, with **9** showing the smallest overall anisotropy; however, there are no direct trends observed between any of these *A* components and the forbidden transition intensity.

Finally, our initial motivation of studying these systems for finding the triplet/singlet forbidden transition needs review (Figure 9). In [Cu_2_(tpy)_2_(μ-CA)]­(PF_6_)_2_, Folgado and co-workers reported |*J*| ≈ 0.04–0.12 cm^–1^, where even subtle distortions of the Cu­(II) geometry and lattice contraction measurably altered the singlet–triplet gap and enabled detection of these spin-forbidden transitions by EPR.^4^ Likewise, in [Cu­(tren)­(μ-SCN)]_2_, Wieghardt and co-workers observed similarly weak exchange (|*J*| ≈ 0.01–0.06 cm^–1^), with counterion coordination playing a decisive role in whether forbidden transitions were observed.^31^ However, we were unable to reproduce these observations in any of the complexes, whether in the solution or solid state. For **1–3** and **4–6**, the absence of the transition is likely straightforward: the *J* values are orders of magnitude larger than the above proofs of concept, and any forbidden singlet–triplet transitions likely occur at fields much higher than the limit of our spectrometer. Even then, the computations showed that the principal axes of the dicopper complexes are close to the same alignment as the individual ions, which would disable one of the proposed mechanisms for enabling the transition.^18–20^ For **7–9**, however, the situation is more clouded. These systems exhibit the closest chemical similarity to the tpy-capped dinuclear compound above, yet in none of the powder nor solution spectra do we see signals stemming from forbidden signals. This is true even for 9 which is reported to have a *J* < 1 cm^–1^, which means the transition should be present for our species. Yet, no clear forbidden transition is seen for **9**. As we note in our synthesis section, many different synthetic procedures were tried but phase purity was never achieved, which may be part of the challenge.

## Conclusions

Enabling addressability for all the transitions in a two-qubit system remains a stark challenge. Here, we assessed the ways in which capping ligand and counterion choices do (and do not) influence these and related properties in dicopper complexes. We also investigated the stability of the formed compounds under the conditions of our measurements.

First, our findings show a remarkably robust dicopper core that shows a modest counterion influence depending on the capping ligand. Outside of the magnetic coupling, however, most other spin-Hamiltonian parameters are relatively insensitive to the large chemical changes that are varied in **1–9**. NEVPT2 calculations also reveal that the small zero-field splitting and axial *g*-tensor in the triplets of these dicopper­(II) complexes are governed by local square-pyramidal ligand fields, which fix the magnetic axes and enable tunable anisotropy without perturbing the exchange-coupled electronic structure. Owing to the large molecular size and the need to accurately capture spin–orbit coupling and multiconfigurational effects, these calculations are computationally demanding, yet they provide critical insight into the magneto-structural origins of the observed EPR behavior.

Second, our work did not reproduce earlier studies of singlet–triplet transitions, but nevertheless showed that another forbidden transition, the Δ*M*_S_ = ±2 transition, can be highly useful in assessing the stability of the studied complexes. Indeed, solid-state and frozen-solution spectra overlay closely, which provides key evidence that the Cu_2_ core remains intact in most solutions, with DMSO proving the most prone to breaking the dinuclear species apart.

Finally, toward the realization of the elusive singlet/triplet transition, a future path for molecules with smaller *J* values is clear. Yet, complex **9**, which exhibited very weak coupling (*J* ≈ −0.63 or 2*J* ≈ −1.26 cm^–1^), yielded no singlet–triplet transition, suggesting that a small exchange magnitude alone does not guarantee forbidden transition observability. One possibility is that intensity will be possible for asymmetric complexes that misalign the *g* axes of the individual Cu­(II) ions. Future studies, guided by computational modeling of the resultant species, will test these ideas further.

## Supplementary Material



## Notes

Full experimental details, sample preparation and analysis, additional magnetic resonance characterization, and computational details (PDF)
