# Supplementary material for: Structure, Stability, and Spin Resonance in Dicopper(II) Complexes
Source: Inorg Chem. 2026 Jul 6;65(28):16092–108. doi: 10.1021/acs.inorgchem.6c00933 (PMC13390041; doi:10.1021/acs.inorgchem.6c00933)
Supplement: Supplementary file 1 [file ic6c00933_si_001.pdf]

Supporting Information for:

Structure, Stability, and Spin Resonance in Dicopper(II) Complexes

Ökten Üngör[1], Nicholas Yiching Chiang[1], Alexander Yu Sokolov[1], Joseph M. Zadrozny*[,1]

[1] Department of Chemistry and Biochemistry, The Ohio State University, Columbus, OH 43210, USA.
Email: zadrozny.13@osu.edu

Table of Contents

Table of Contents	Page
**Table S1:** Crystallographic information for structural refinement of **1**.	S4
**Table S2:** Crystallographic information for structural refinement of **1a**.	S5
**Table S3:** Crystallographic information for structural refinement of **2**.	S6
**Table S4:** Crystallographic information for structural refinement of **3**.	S7
**Table S5:** Crystallographic information for structural refinement of **4**.	S8
**Table S6:** Crystallographic information for structural refinement of **5**.	S9
**Table S7:** Crystallographic information for structural refinement of **6**.	S10
**Table S8:** Crystallographic information for structural refinement of **7**.	S11
**Table S9:** Crystallographic information for structural refinement of **8**.	S12
**Table S10:** Relative energies of states (cm^{-1}) and principal *g*-values in **1**, **1*** and **1a**.	S13
**Table S11:** Relative energies of states (cm^{-1}) and principal *g*-values in **1***.	S14
**Table S12:** Computed coordinates of optimized geometry of **1**.	S15-S17
**Table S13:** Computed coordinates of optimized geometry of **1***.	S18-S19
**Figure S1:** Experimental and calculated PXRD patterns for **1**.	S20
**Figure S2:** Experimental and calculated PXRD patterns for **2**.	S20
**Figure S3:** Experimental and calculated PXRD patterns for **3**.	S21
**Figure S4:** Experimental and calculated PXRD patterns for **4**.	S21
**Figure S5:** Experimental and calculated PXRD patterns for **5**.	S22
**Figure S6:** Experimental and calculated PXRD patterns for **6**.	S22
**Figure S7:** Experimental and calculated PXRD patterns for **7**.	S23
**Figure S8:** Experimental and calculated PXRD patterns for **9**.	S23
**Figure S9:** Solvent-dependent UV-Vis spectra for **1**.	S24
**Figure S10:** Solvent-dependent UV-Vis spectra for **1** in situ.	S24
**Figure S11:** Solvent-dependent UV-Vis spectra for **1a**.	S25
**Figure S12:** Solvent-dependent UV-Vis spectra for **2**. .	S25
**Figure S13:** Solvent-dependent UV-Vis spectra for **3**. .	S26
**Figure S14:** Solvent-dependent UV-Vis spectra for **4**.	S26
**Figure S15:** Solvent-dependent UV-Vis spectra for **5**.	S27
**Figure S16:** Solvent-dependent UV-Vis spectra for **6**.	S27
**Figure S17:** UV-Vis spectrum of **7** in MeCN.	S28
**Figure S18:** UV-Vis spectrum of **8** in DMSO.	S28
**Figure S19:** UV-Vis spectrum of **9** in DMSO.	S29
**Figure S20:** cw EPR spectra of **1** measured in various solvents at 5 K.	S29
**Figure S21:** cw EPR spectra and simulation of **1a** measured under various conditions.	S30
**Figure S22:** cw EPR spectra and simulation of **2** measured under various conditions.	S31
**Figure S23:** cw EPR spectra and simulation of **3** measured under various conditions.	S32
**Figure S24:** cw EPR spectra and simulation of **4** measured under various conditions.	S33
**Figure S25:** cw EPR spectra and simulation of **5** measured under various conditions.	S34
**Figure S26:** cw EPR spectra and simulation of **6** measured under various conditions.	S35
**Figure S27:** cw EPR spectrum of **7**.	S36
**Figure S28:** cw EPR spectrum of **8**.	S36
**Figure S29:** cw EPR spectra and simulation of **9** measured under various conditions.	S37

Figure S30. Temperature dependence of χ_M and peak-to-peak EPR signal intensity comparison for **1**. S38
Figure S31: Natural frontier orbitals of complex **1*** obtained from (18e, 10o) CASSCF. S38
Figure S32: Natural frontier orbitals of complex **1*** obtained from (10e, 10o) CASSCF S39
Figure S33: Natural frontier orbitals of complex **1*** based on the optimized structure. S39
Figure S34: (9e,5o) SA-CASSCF active orbitals of X-ray structure **1a**. S40
Figure S35: (18e,10o) SA-CASSCF active orbitals of X-ray structure **1***. S40
Figure S36: (18e,10o) SA-CASSCF active orbitals of optimized structure **1***. S40
Figure S37: (10e,10o) SA-CASSCF active orbitals of optimized structure **1**. S41
Figure S38: (10e,10o) SA-CASSCF active orbitals of optimized structure **1***. S41
Figure S39: (10e,9o) SA-CASSCF active orbitals of X-ray structure **1**. S42
Figure S40: (9o,10e) SA-CASSCF active orbitals of X-ray structure **1***. S43
FTIR Interpretation Details. S44
References S45

Table S1. Crystallographic information for the structural refinement of **1**.

Empirical formula	$C_{24}H_{42}Cl_4Cu_2N_6O_{12}$
Formula weight	875.51 g/mol
Temperature	110(10)) K
Crystal system	Monoclinic
Space group	$P2_1/n$
a	9.4430(16) Å
b	13.4660(16) Å
c	13.6766(16) Å
α	90°
β	96.246(6)°
γ	90°
Volume	1728.8(4) $Å^3$
Z	2
ρ_{calc}	1.682 g cm^{-3}
μ	1.606 mm^{-1}
Crystal color	Dark brown
Crystal size	0.244 × 0.130 × 0.107 mm^3
Radiation	MoKα (λ = 0.71073 Å)
2θ range for data collection	2.50 to 30.50°
Reflections collected	17861
Independent collections	5281 [R_{int} = 0.0369, R_{sigma} = 0.0100]
Data/restraints/parameters	5190/0/220
Goodness-of-fit on F^2	1.095
Final R indexes [I > 2σ(I)]	R_1 = 0.0190, wR_2 = 0.0510
Final R indexes [all data]	R_1 = 0.0194, wR_2 = 0.0513
Largest diff. peak/hole	0.514/–0.391 e $Å^{-3}$

Table S2. Crystallographic information for the structural refinement of **1a**.

Empirical formula	$C_{15}H_{21}Cl_2CuN_3O_4$
Formula weight	441.80 g/mol
Temperature	100(10) K
Crystal system	Triclinic
Space group	P-1
a	11.4318(7)
b	11.5597(7)
c	14.8416(10)
α	109.150(2)
β	98.411(2)
γ	90.916(2)
Volume	1828.5(2)
Z	4
ρ_{calc}	1.605
μ	1.515
Crystal color	Violet
Crystal size	0.040 × 0.104 × 0.195 mm^3
Radiation	MoKα (λ = 0.71073 Å)
2θ range for data collection	1.86 to 27.93
Reflections collected	73563
Independent collections	8642 [R_{int} = 0.0229, R_{sigma} = 0.0123]
Data/restraints/parameters	8642/2/474
Goodness-of-fit on F^2	1.170
Final R indexes [I > 2σ(I)]	R_1 = 0.0244, wR_2 = 0.0815
Final R indexes [all data]	R_1 = 0.0273, wR_2 = 0.0925
Largest diff. peak/hole	0.587/–0.482 e $Å^{-3}$

Table S3. Crystallographic information for the structural refinement of **2**.

Empirical formula	$C_{13}H_{21}ClCuF_3N_3O_5S$
Formula weight	487.38 g/mol
Temperature	100(10)) K
Crystal system	Monoclinic
Space group	C2/c
a	26.7377(14) Å
b	8.7809(4) Å
c	17.1260(9) Å
α	90°
β	99.5350(10)°
γ	90°
Volume	3965.3(3) $Å^3$
Z	8
ρ_{calc}	1.633 g cm^{-3}
μ	1.398 mm^{-1}
Crystal color	Dark brown
Crystal size	0.112 × 0.169 × 0.321 mm^3
Radiation	MoKα (λ = 0.71073 Å)
2θ range for data collection	2.44 to 27.92°
Reflections collected	37764
Independent collections	4935 [R_{int} = 0.0259, R_{sigma} = 0.0171]
Data/restraints/parameters	4935/0/248
Goodness-of-fit on F^2	1.159
Final R indexes [I > 2σ(I)]	R_1 = 0.0355, wR_2 = 0.0996
Final R indexes [all data]	R_1 = 0.0379, wR_2 = 0.1113
Largest diff. peak/hole	1.457/–0.689 e $Å^{-3}$

Table S4. Crystallographic information for the structural refinement of **3**.

Empirical formula	$C_{24}H_{46}Cl_4Cu_2N_6O_6$
Formula weight	783.55 g/mol
Temperature	110(10) K
Crystal system	Monoclinic
Space group	$P2_1/c$
a	13.7807(6) Å
b	8.5888(4) Å
c	14.6175(6) Å
α	90°
β	112.068(2)°
γ	90°
Volume	1603.37(12) $Å^3$
Z	2
ρ_{calc}	1.623 g cm^{-3}
μ	1.708 mm^{-1}
Crystal color	Green
Crystal size	0.052 × 0.064 × 0.132 mm^3
Radiation	MoKα (λ = 0.71073 Å)
2θ range for data collection	2.808 to 29.529°
Reflections collected	10679
Independent collections	2942 [R_{int} = 0.0833, R_{sigma} = 0.0856]
Data/restraints/parameters	2942/0/197
Goodness-of-fit on F^2	1.030
Final R indexes [I > 2σ(I)]	R_1 = 0.0498, wR_2 = 0.1089
Final R indexes [all data]	R_1 = 0.0.0601, wR_2 = 0.1232
Largest diff. peak/hole	0.590/–0.564 e $Å^{-3}$

Table S5. Crystallographic information for the structural refinement of **4**.

Empirical formula	$C_{26}H_{44}Cl_4Cu_2N_4O_{12}$
Formula weight	873.53 g/mol
Temperature	110(10)) K
Crystal system	Monoclinic
Space group	$P2_1$
a	8.5342(3) Å
b	14.1782(6) Å
c	14.8649(7) Å
α	90°
β	96.805(1)°
γ	90°
Volume	1785.98(13) $Å^3$
Z	2
ρ_{calc}	1.624 g cm^{-3}
μ	1.553 mm^{-1}
Crystal color	blue
Crystal size	0.108 × 0.133 × 0.215 mm^3
Radiation	MoKα (λ = 0.71073 Å)
2θ range for data collection	1.992 to 30.567°
Reflections collected	65862
Independent collections	10589 [R_{int} = 0.3270, R_{sigma} = 0.1661]
Data/restraints/parameters	9595/1/442
Goodness-of-fit on F^2	1.040
Final R indexes [I > 2σ(I)]	R_1 = 0.0291, wR_2 = 0.0594
Final R indexes [all data]	R_1 = 0.0353, wR_2 = 0.0731
Largest diff. peak/hole	0.556/–0.463 e $Å^{-3}$

Table S6. Crystallographic information for the structural refinement of **5**.

Empirical formula	$C_{28}H_{44}Cu_2F_6N_4O_{10}S_2$
Formula weight	972.77 g/mol
Temperature	110(10)) K
Crystal system	Monoclinic
Space group	C2/c
a	8.3457(2) Å
b	14.5303(4) Å
c	16.0224(4) Å
α	90°
β	96.8560(10)°
γ	90°
Volume	1929.07(9) $Å^3$
Z	2
ρ_{calc}	1.675 g cm^{-3}
μ	1.435 mm^{-1}
Crystal color	Blue
Crystal size	0.462 × 0.211 × 0.105 mm^3
Radiation	MoKα (λ = 0.71073 Å)
2θ range for data collection	2.56 to 28.29°
Reflections collected	39854
Independent collections	9489 [R_{int} = 0.0483, R_{sigma} = 0.0431]
Data/restraints/parameters	8913/1/497
Goodness-of-fit on F^2	0.978
Final R indexes [I > 2σ(I)]	R_1 = 0.0255, wR_2 = 0.0667
Final R indexes [all data]	R_1 = 0.0277, wR_2 = 0.0685
Largest diff. peak/hole	0.404/–0.461 e $Å^{-3}$

Table S7. Crystallographic information for the structural refinement of **6**.

Empirical formula	$C_{26}H_{44}Cl_4Cu_2N_4O_5$
Formula weight	763.54 g/mol
Temperature	110(10) K
Crystal system	Monoclinic
Space group	C2
a	15.598(7) Å
b	7.682(4) Å
c	28.541(15) Å
α	90°
β	104.324(13)°
γ	90°
Volume	3314(3) $Å^3$
Z	4
ρ_{calc}	1.531 g cm^{-3}
μ	1.647 mm^{-1}
Crystal color	Green
Crystal size	0.213 × 0.135 × 0.083 mm^3
Radiation	MoKα (λ = 0.71073 Å)
2θ range for data collection	2.209 to 26.401°
Reflections collected	28195
Independent collections	6782 [R_{int} = 0.1292, R_{sigma} = 0.0971]
Data/restraints/parameters	6782/4/396
Goodness-of-fit on F^2	1.087
Final R indexes [I > 2σ(I)]	R_1 = 0.0435, wR_2 = 0.0885
Final R indexes [all data]	R_1 = 0.0502, wR_2 = 0.0912
Largest diff. peak/hole	0.636/–0.618 e $Å^{-3}$

Table S8. Crystallographic information for the structural refinement of **7**.

Empirical formula	$C_{38}H_{24}Cl_4Cu_2F_6N_6O_{12}S_2$
Formula weight	1203.63
Temperature	100(10) K
Crystal system	Triclinic
Space group	P-1
a	9.8093(3) Å
b	10.7767(3) Å
c	11.5953(3) Å
α	65.529(10) °
β	86.046(10) °
γ	89.492(10) °
Volume	1112.73(5) $Å^3$
Z	1
ρ_{calc}	1.796
μ	1.384
Crystal color	Green
Crystal size	0.135 × 0.093 × 0.061 mm^3
Radiation	Mo Kα (λ = 0.71073 Å)
2θ range for data collection	3.870 to 56.706°
Reflections collected	42530
Independent reflections	5508 [R_{int} = 0.0414, R_{sigma} = 0.0263]
Data/restraints/parameters	5508/0/330
Goodness-of-fit on F^2	1.185
Final R indexes [I > 2σ(I)]	R_1 = 0.0252, wR_2 = 0.0684
Final R indexes [all data]	R_1 = 0.0263, wR_2 = 0.0754
Largest diff. peak/hole	0.468/–0.431 e $Å^{-3}$

Table S9. Crystallographic information for the structural refinement of **8**.

Empirical formula	$C_{38}H_{24}Br_2Cl_2Cu_2F_6N_6O_{12}S_2$
Formula weight	1292.55
Temperature	100(10) K
Crystal system	Triclinic
Space group	P-1
a	9.894(3) Å
b	10.844(4) Å
c	11.556(5) Å
α	65.244(13) °
β	85.316(12) °
γ	89.477(7) °
Volume	1121.7(7) $Å^3$
Z	1
ρ_{calc}	1.910
μ	3.035
Crystal color	Purple
Crystal size	0.12 × 0.052 × 0.040 mm^3
Radiation	MoKα (λ = 0.71073 Å)
2θ range for data collection	4.132 to 56.710°
Reflections collected	11029
Independent collections	5569 [R_{int} = 0.0187, R_{sigma} = 0.0279]
Data/restraints/parameters	5569/0/322
Goodness-of-fit on F^2	1.043
Final R indexes [I > 2σ(I)]	R_1 = 0.0263, wR_2 = 0.0655
Final R indexes [all data]	R_1 = 0.0320, wR_2 = 0.0676
Largest diff. peak/hole	0.586/–0.388 e $Å^{-3}$

Table S10. Relative energies of states (cm^{-1}) and principal *g* values in **1**, **1***, and **1a** calculated using NEVPT2. Four singlet states and four triplet states with equal weight were included for **1** with and without ClO_4^- , while five doublet states with equal weight were computed for **1a.**

	**1**		**1***		**1a**
	Optimized structure	X-ray structure	Optimized structure	X-ray structure	X-ray structure
state	(10e,10o)	(10e,9o)	(10e,10o)	(10e,9o)	(9e,5o)
0	0.00	0.00	0.00	0.00	0.00
1	2.31	3.15	3.26	2.28	0.00
2	2.31	3.15	3.27	2.28	11602.54
3	2.31	3.15	3.27	2.28	11602.54
4	4849.90	8796.39	7946.17	11148.43	14724.09
5	4850.04	8796.40	7948.19	11149.90	14724.09
6	4850.04	8796.40	7948.99	11153.42	16125.32
7	4850.04	8798.48	7948.99	11153.42	16125.32
8	4920.80	8843.18	7949.12	11158.08	17040.67
9	4920.80	8843.18	7954.40	11158.08	17040.67
10	4920.80	8843.18	7954.53	11161.89	
11	4923.54	8843.29	7954.53	11163.07	
12	9779.49	17640.49	15819.41	14104.72	
13	9779.62	17640.49	15822.04	14104.73	
14	9779.62	17640.49	15822.05	14104.73	
15	9779.62	17640.78	15822.05	14162.02	
g_z	2.04	2.02	2.06	2.02	2.38
g_x	2.00	2.00	2.00	2.00	2.08
g_y	2.00	2.00	2.00	2.00	2.08

Table S11. Relative energies of states (cm^{-1}) and principal *g*-values in **1*** calculated using NEVPT2. The calculations incorporated 24 singlet and 24 triplet states with equal weights.

Active space	(18e, 10o)		(10e, 9o)
state	Optimized structure	X-ray structure	X-ray structure
0	0.00	0.00	0.00
1	2.05	2.12	9.96
2	2.09	2.13	9.96
3	2.10	2.18	9.96
4	7931.25	10883.51	10936.51
5	7933.19	10883.60	10936.51
6	7941.39	10883.84	10936.55
7	7941.42	10884.09	10937.71
8	7941.64	10887.20	10944.28
9	7946.26	10889.03	10944.63
10	7946.53	10889.09	10944.68
11	7946.76	10889.57	10944.68
12	11212.70	14314.35	14547.32
13	11213.39	14314.73	14547.32
14	11213.89	14315.40	14548.29
15	11213.92	14315.42	14606.27
g_z	2.50	2.40	2.03
g_x	2.10	2.07	2.00
g_y	2.11	2.09	2.00

Table S12. Computed coordinates of optimized geometry of **1** (unit: Å).

Cu	1.954698	9.811403	5.572671
Cl	2.854480	7.169303	9.754261
Cl	4.999593	11.154782	6.423687
O	4.816227	5.118135	8.725664
O	2.212825	8.669034	7.216457
O	3.570093	11.236612	6.057875
O	5.223136	12.054750	7.506278
N	-0.047571	8.743834	4.833890
O	5.276745	9.800797	6.810561
O	5.762490	11.524276	5.272672
N	1.604036	10.907983	3.739809
N	0.492111	11.174268	6.384233
C	3.047650	7.768504	7.117850
C	4.368728	5.909089	7.893015
C	-1.139035	9.517746	5.424449
H	-1.584734	10.165460	4.672844
H	-1.946989	8.858212	5.750437
C	0.123980	8.905700	3.395115
H	0.934288	8.244062	3.092361
H	-0.770382	8.595255	2.838075
C	0.992867	11.731936	7.651535
H	1.267411	10.920889	8.321065
H	1.882160	12.325159	7.467996
H	0.227312	12.351697	8.128769
C	0.260981	12.229741	5.375986
H	-0.730150	12.104963	4.946543
H	0.252449	13.209387	5.855207
C	3.479270	6.944836	8.151874
C	-0.085949	7.339621	5.227912
H	0.804340	6.826278	4.873491
H	-0.110307	7.256834	6.312163
H	-0.964643	6.827615	4.818616
C	-0.691114	10.336406	6.635156
H	-0.429693	9.657393	7.445058
H	-1.530710	10.949327	6.983254
C	2.834757	10.902500	2.932776
H	3.676630	11.227255	3.535647
H	3.038746	9.891053	2.591105
H	2.731679	11.562424	2.065749

C	0.470052	10.336175	2.988744
H	-0.398980	10.978484	3.108047
H	0.691185	10.342102	1.920118
C	1.320130	12.249333	4.270530
H	2.251158	12.638456	4.670350
H	0.990209	12.925941	3.473976
Cu	5.988306	3.646683	8.000288
Cl	5.125838	6.315592	3.830192
Cl	2.918904	2.365737	7.118975
O	3.152929	8.357272	4.855368
O	5.758997	4.808621	6.366179
O	4.344878	2.259823	7.491144
O	2.688758	1.483271	6.023414
N	8.007402	4.661930	8.760457
O	2.662191	3.728451	6.748378
O	2.144599	1.991917	8.260927
N	6.308234	2.518712	9.819675
N	7.425699	2.264164	7.175864
C	4.929538	5.714400	6.465767
C	3.607329	7.572958	5.690114
C	9.085785	3.874506	8.163798
H	9.515138	3.207941	8.908372
H	9.908162	4.522065	7.850037
C	7.827294	4.485438	10.196425
H	7.029451	5.159544	10.504785
H	8.725653	4.770456	10.760553
C	6.918950	1.733198	5.899501
H	6.664183	2.558244	5.239372
H	6.016741	1.156329	6.071843
H	7.673529	1.103731	5.417517
C	7.631134	1.191332	8.171283
H	8.622828	1.290237	8.606167
H	7.621681	0.217944	7.679509
C	4.502526	6.541342	5.432961
C	8.073388	6.069786	8.383872
H	7.190922	6.594452	8.741224
H	8.103570	6.165148	7.300790
H	8.959771	6.560396	8.802735
C	8.626526	3.081158	6.940537
H	8.382015	3.775817	6.138673
H	9.455047	2.456091	6.587537

C	5.074471	2.538686	10.621809
H	4.228926	2.238095	10.011601
H	4.889135	3.549718	10.975240
H	5.160683	1.866210	11.480984
C	7.450448	3.057219	10.582830
H	8.306672	2.398872	10.458480
H	7.224665	3.041809	11.650379
C	6.567213	1.178949	9.272330
H	5.630206	0.813862	8.863916
H	6.880015	0.485365	10.061167

Table S13. Computed coordinates of optimized geometry of **1*** (unit: Å).

Cu	1.615047	9.535859	5.465467
Cl	2.662853	6.995316	9.695163
O	4.896317	5.182219	8.734891
O	2.072501	8.536161	7.155094
N	-0.338040	8.790851	4.681364
N	1.563418	10.805866	3.764410
N	0.424126	11.030321	6.381663
C	2.936390	7.660012	7.082259
C	4.391023	5.932043	7.896918
C	-1.369769	9.636137	5.311640
H	-1.732797	10.366602	4.593767
H	-2.241315	9.037984	5.580165
C	-0.149064	9.035545	3.245371
H	0.564267	8.295304	2.883301
H	-1.077285	8.883573	2.684924
C	0.976521	11.457566	7.680909
H	1.138380	10.592691	8.317163
H	1.932143	11.957183	7.528517
H	0.299514	12.152748	8.181448
C	0.328627	12.172824	5.442238
H	-0.649946	12.174999	4.972401
H	0.394933	13.115198	5.985316
C	3.345866	6.824779	8.118332
C	-0.550002	7.368384	4.962049
H	0.292099	6.787325	4.589754
H	-0.626962	7.210532	6.036407
H	-1.462779	6.995181	4.489779
C	-0.864085	10.330455	6.576826
H	-0.705774	9.588982	7.359203
H	-1.628071	11.023421	6.940439
C	2.802192	10.720757	2.969758
H	3.666497	10.902413	3.604888
H	2.902416	9.724942	2.548108
H	2.797515	11.456704	2.163197
C	0.381057	10.436391	2.943911
H	-0.400717	11.175506	3.091071
H	0.635338	10.487151	1.885509
C	1.429112	12.137501	4.385072
H	2.387227	12.378590	4.847479
H	1.240259	12.908174	3.632694

Cu	6.319009	3.903185	8.101302
Cl	5.271305	6.444022	3.872226
O	3.036825	8.255918	4.831944
O	5.862385	4.903864	6.412512
N	8.267807	4.649229	8.895345
N	6.365744	2.628621	9.799020
N	7.516366	2.412996	7.186071
C	4.997869	5.779438	6.485154
C	3.542629	7.506742	5.670282
C	9.303495	3.807218	8.267243
H	9.664621	3.075410	8.984706
H	10.175247	4.407411	8.003975
C	8.073089	4.400425	10.329868
H	7.356974	5.138513	10.690806
H	8.998662	4.552439	10.894674
C	6.970143	1.988279	5.883381
H	6.809316	2.854553	5.248778
H	6.014781	1.486586	6.030450
H	7.650474	1.295644	5.383819
C	7.609837	1.268141	8.122874
H	8.586444	1.266303	8.596794
H	7.547368	0.327100	7.577035
C	4.588172	6.614407	5.449012
C	8.478699	6.072762	8.619356
H	7.634127	6.651488	8.989652
H	8.559974	6.233610	7.545747
H	9.388912	6.446133	9.096423
C	8.804246	3.115427	6.998143
H	8.648113	3.858598	6.216935
H	9.570815	2.424543	6.635999
C	5.123508	2.709628	10.588668
H	4.262195	2.528053	9.949439
H	5.019813	3.704204	11.012394
H	5.126013	1.971639	11.393372
C	7.544028	2.997924	10.625440
H	8.327619	2.260453	10.479697
H	7.285373	2.944034	11.682627
C	6.504836	1.298859	9.175480
H	5.549086	1.057471	8.708358
H	6.691707	0.526527	9.926653

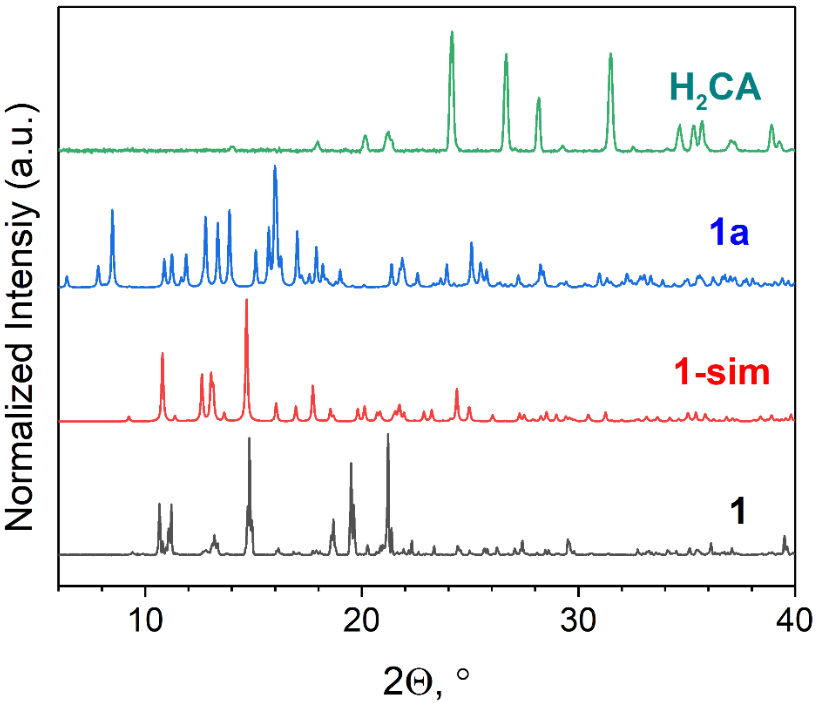

Figure S1. Experimental and calculated PXRD patterns for **1**.

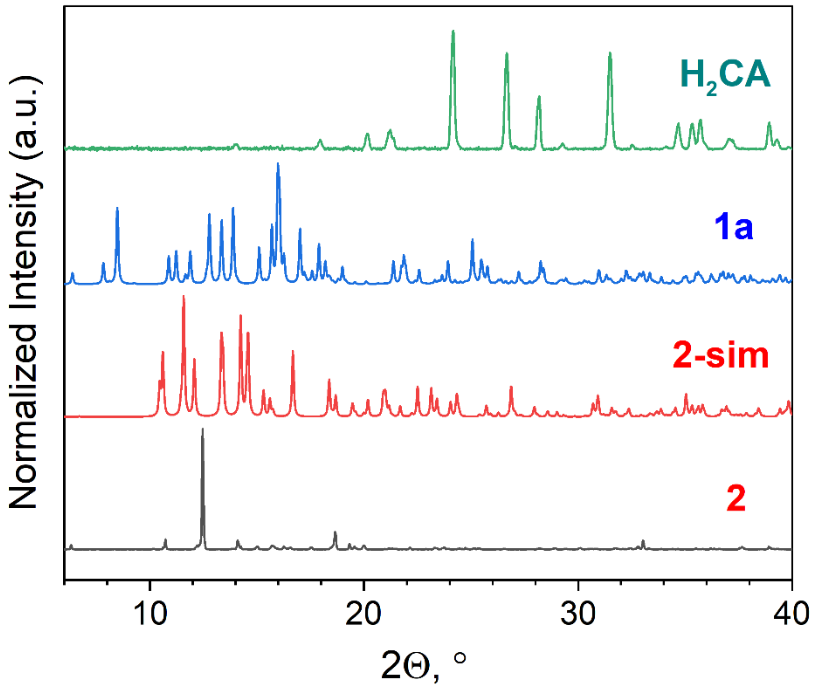

Figure S2. Experimental and calculated PXRD patterns for **2**.

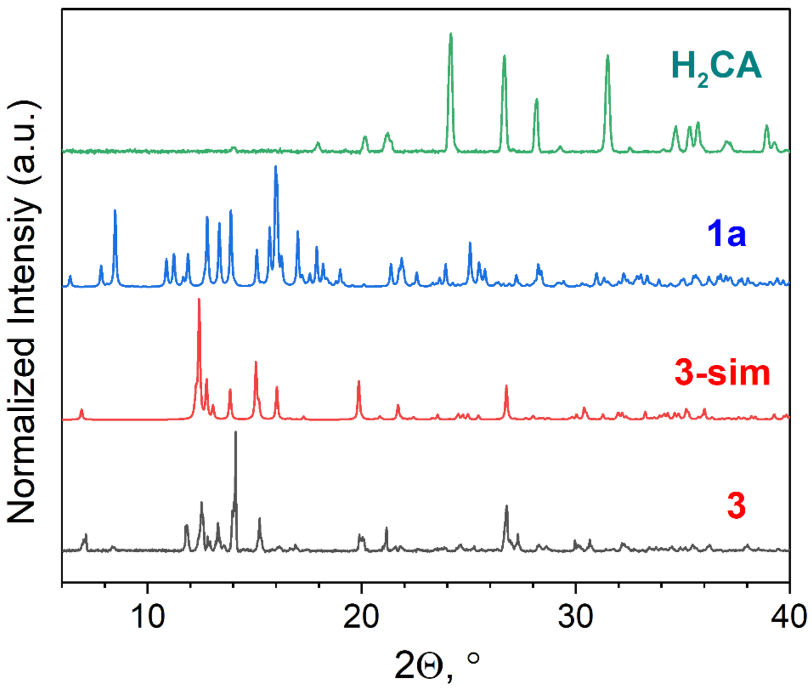

Figure S3. Experimental and calculated PXRD patterns for **3**.

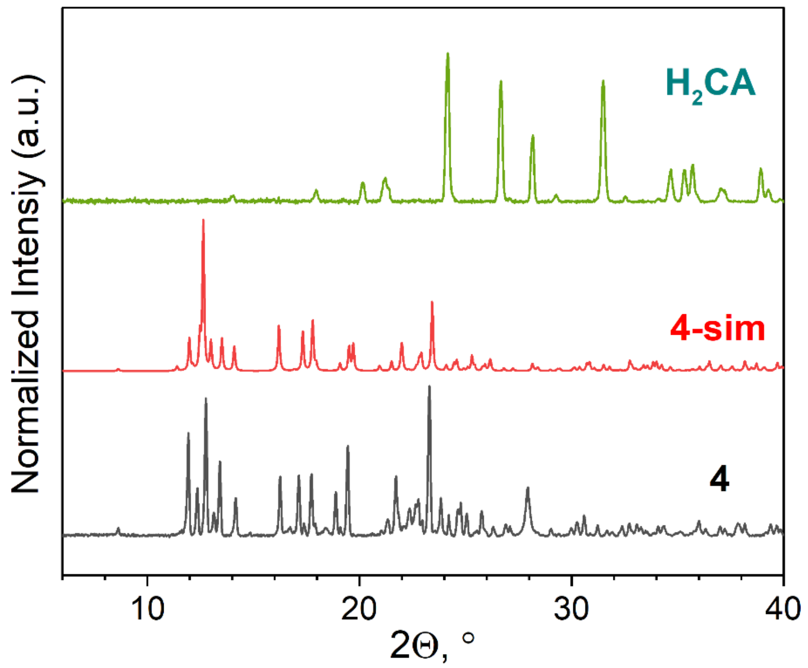

Figure S4. Experimental and calculated PXRD patterns for **4**.

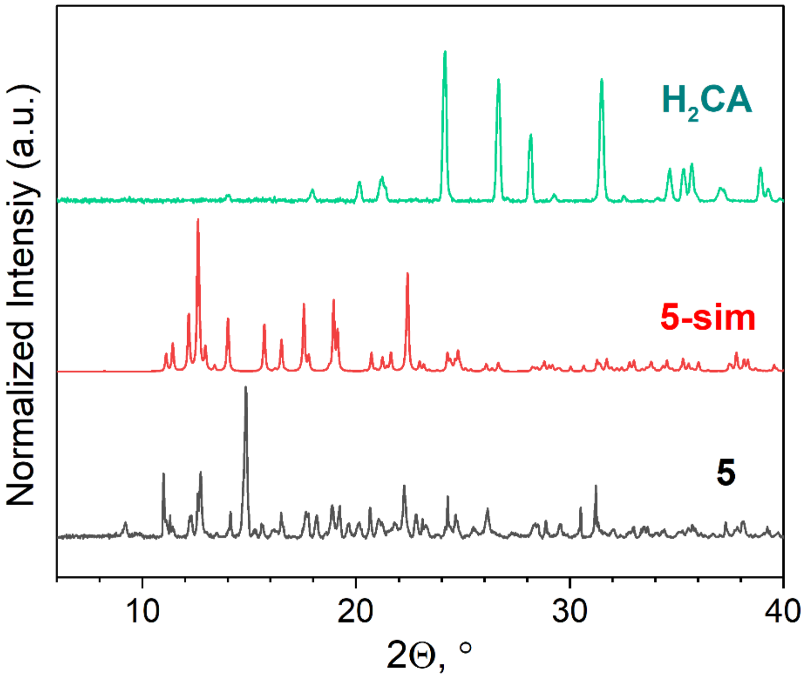

Figure S5. Experimental and calculated PXRD patterns for **5**.

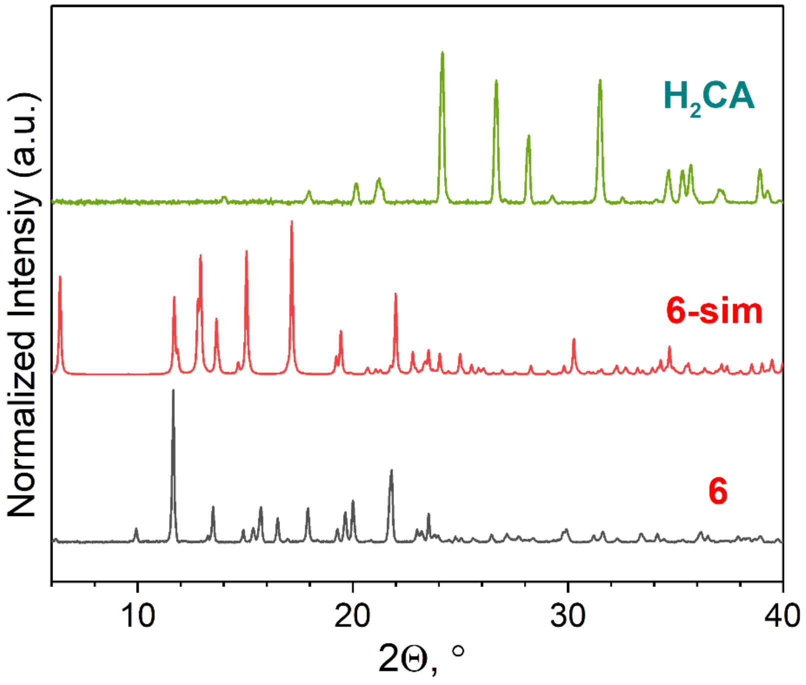

Figure S6. Experimental and calculated PXRD patterns for **6**.

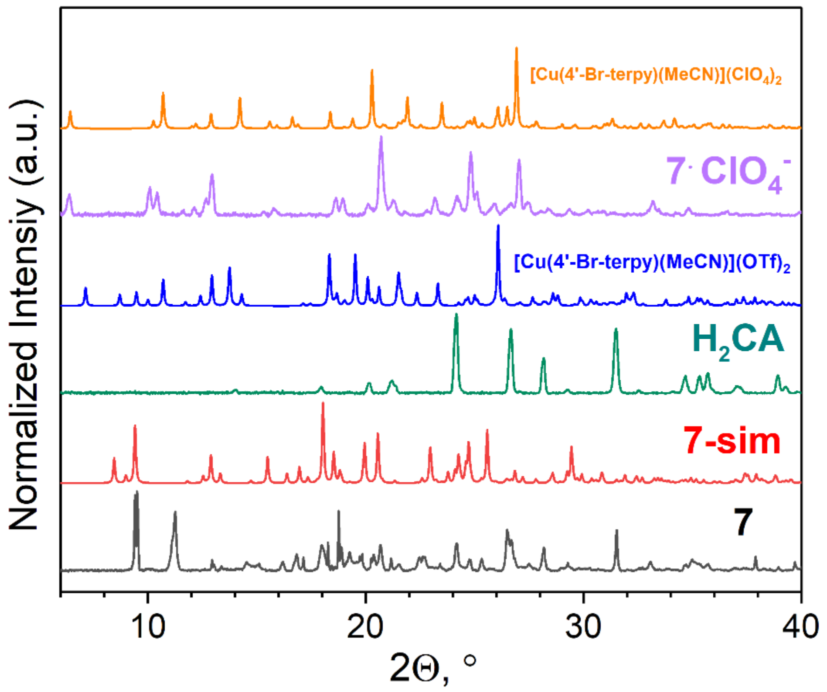

Figure S7. Experimental and calculated PXRD patterns for **7**.

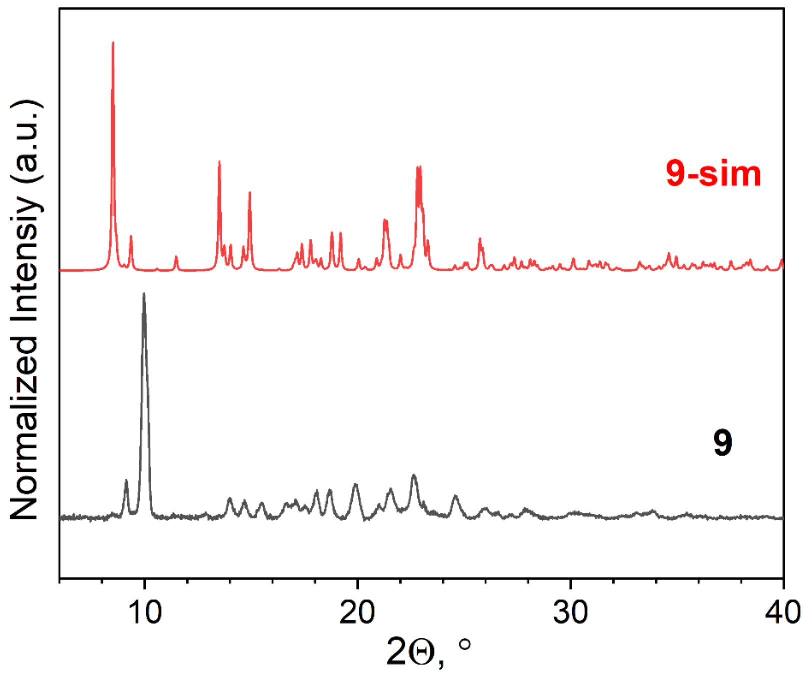

Figure S8. Experimental and calculated PXRD patterns for **9.**

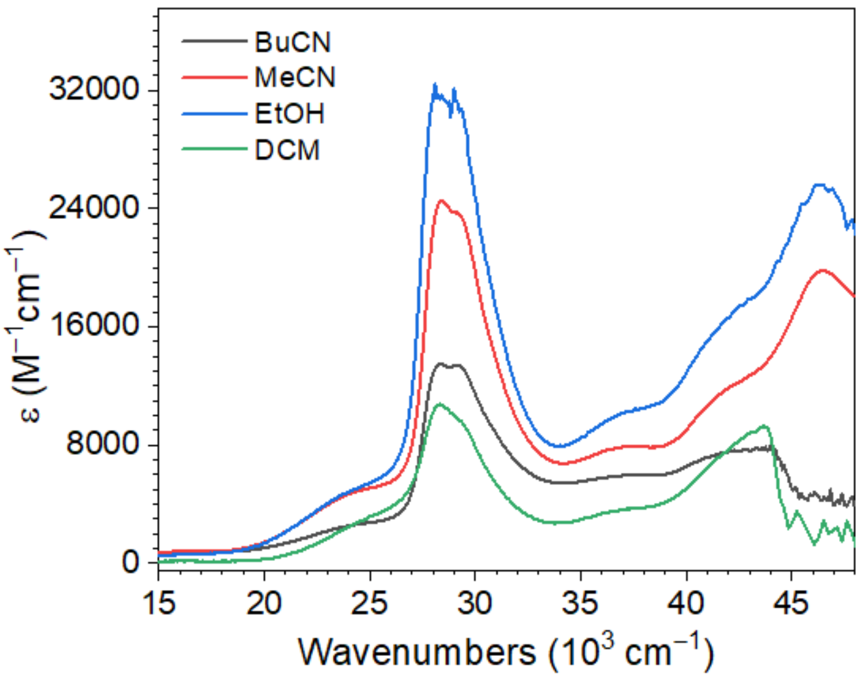

Figure S9. Solvent-dependent UV-Vis spectra for **1**.

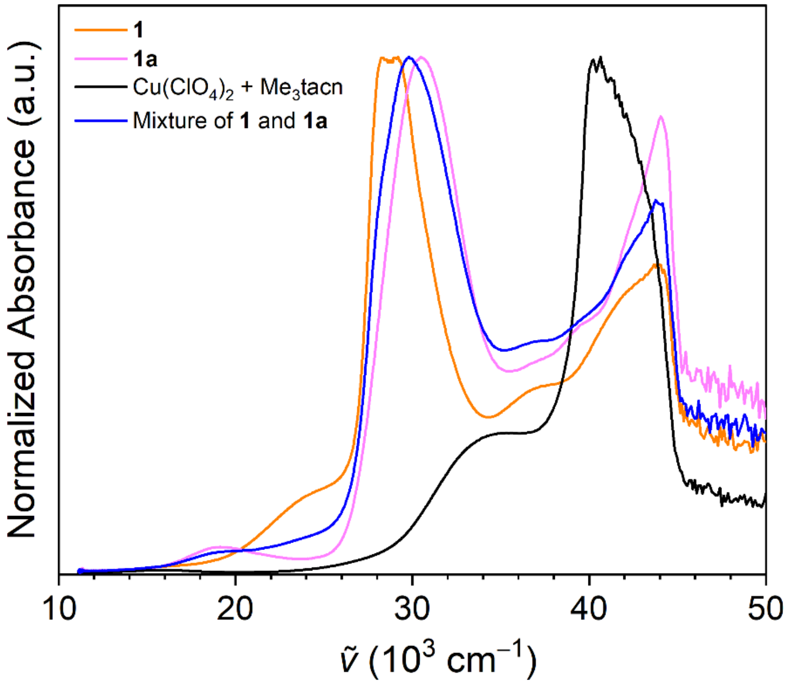

Figure S10. Solvent-dependent UV-Vis spectra for **1** in situ.

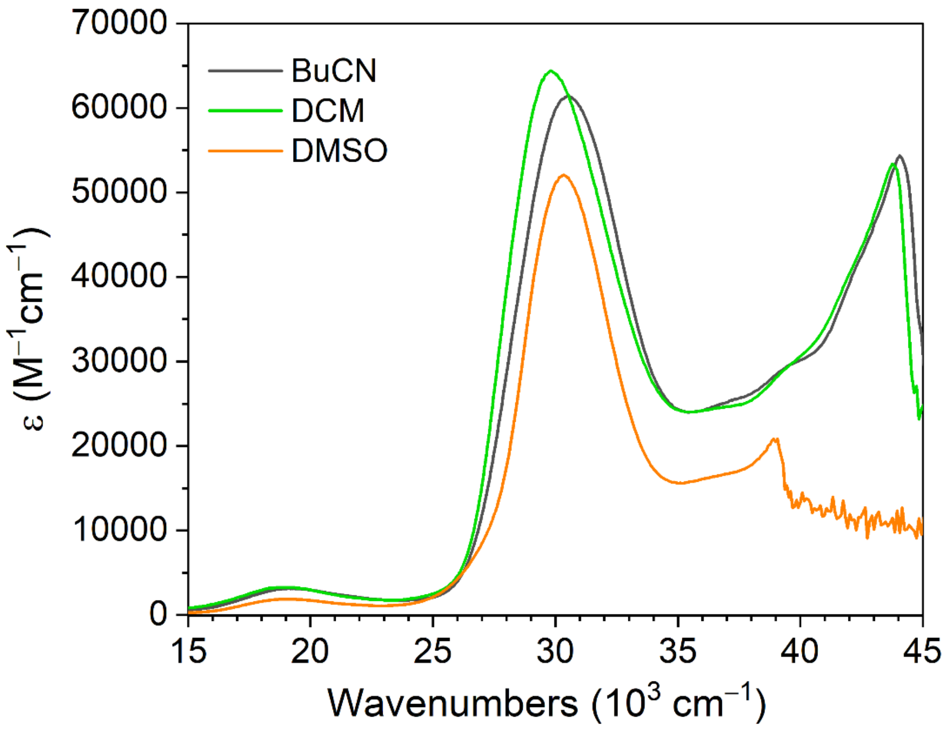

Figure S11. Solvent-dependent UV-Vis spectra for **1a**.

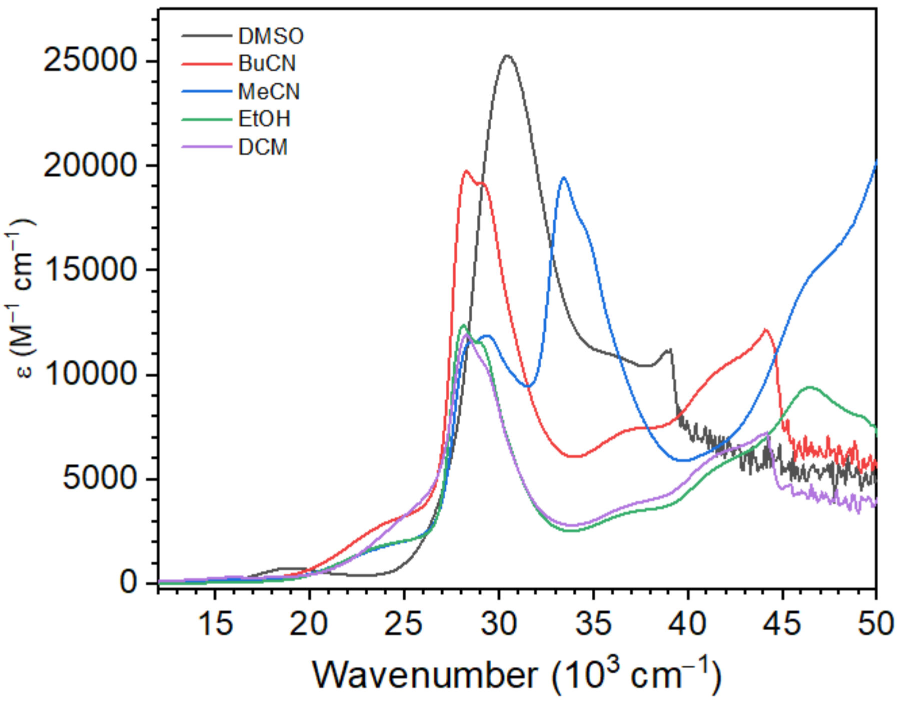

Figure S12. Solvent-dependent UV-Vis spectra for **2**.

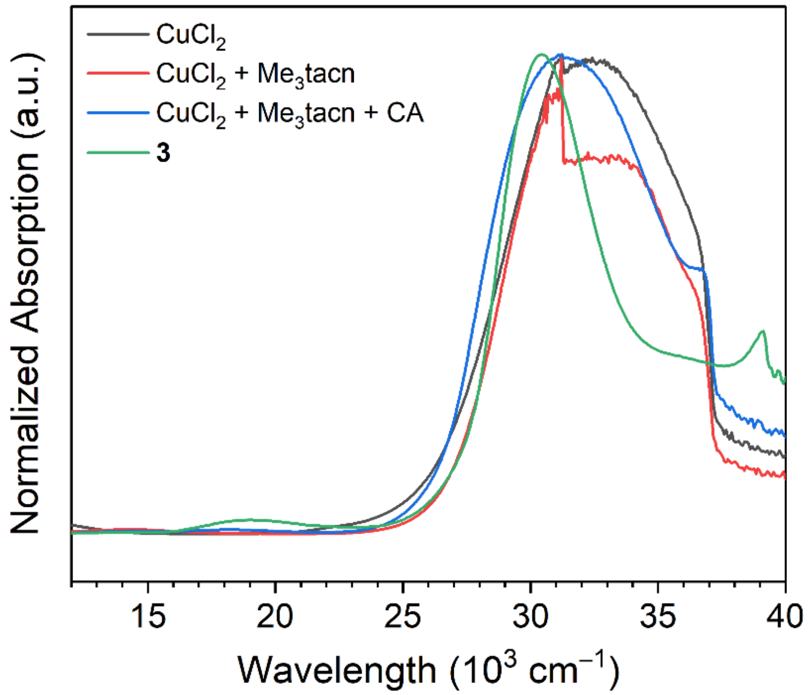

Figure S13. Solvent-dependent UV-Vis spectra for **3**.

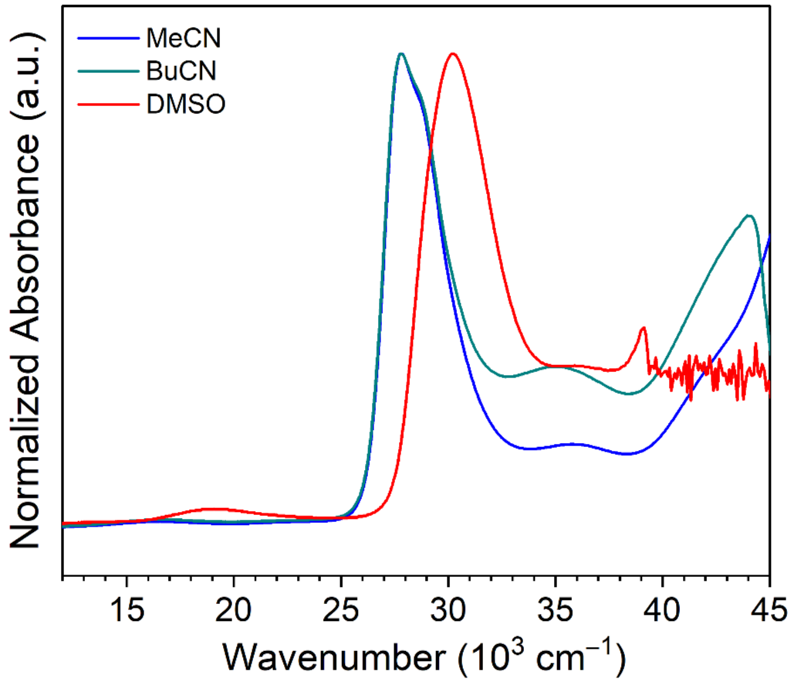

Figure S14. Solvent-dependent UV-Vis spectra for **4**.

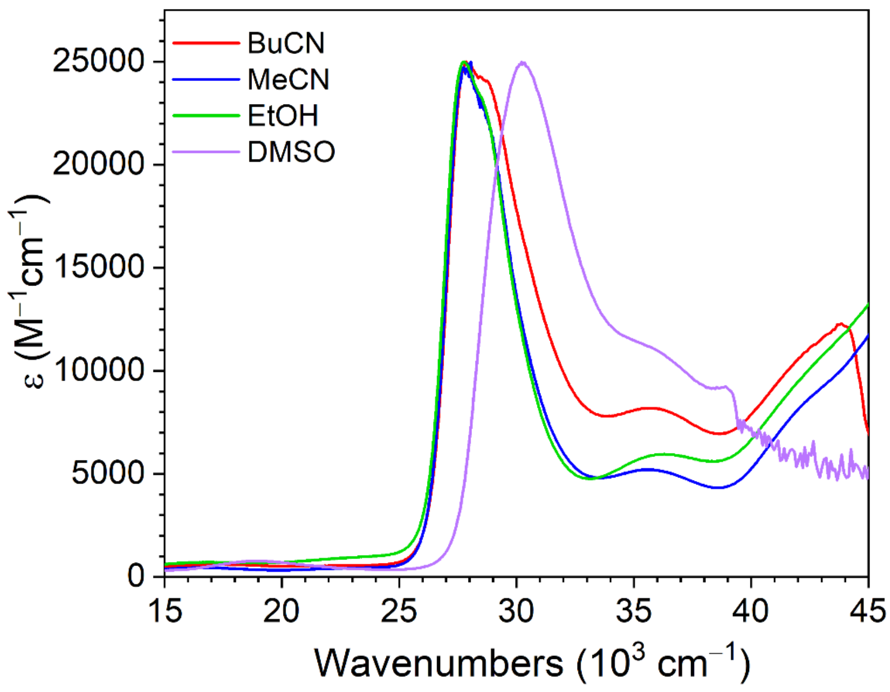

Figure S15. Solvent-dependent UV-Vis spectra for **5**.

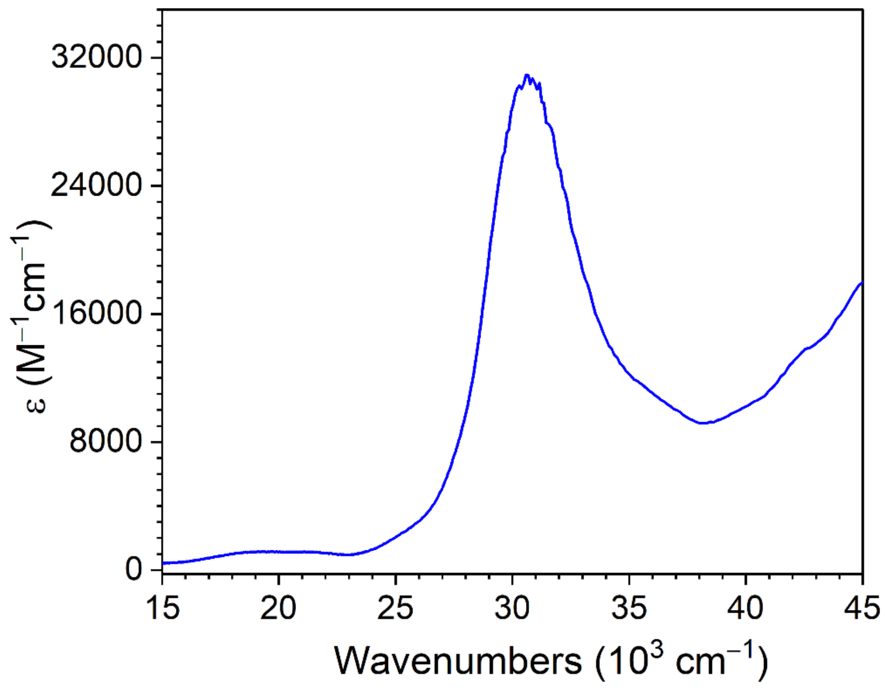

Figure S16. UV-Vis spectrum of 0.01 mM solution of **6** in MeCN.

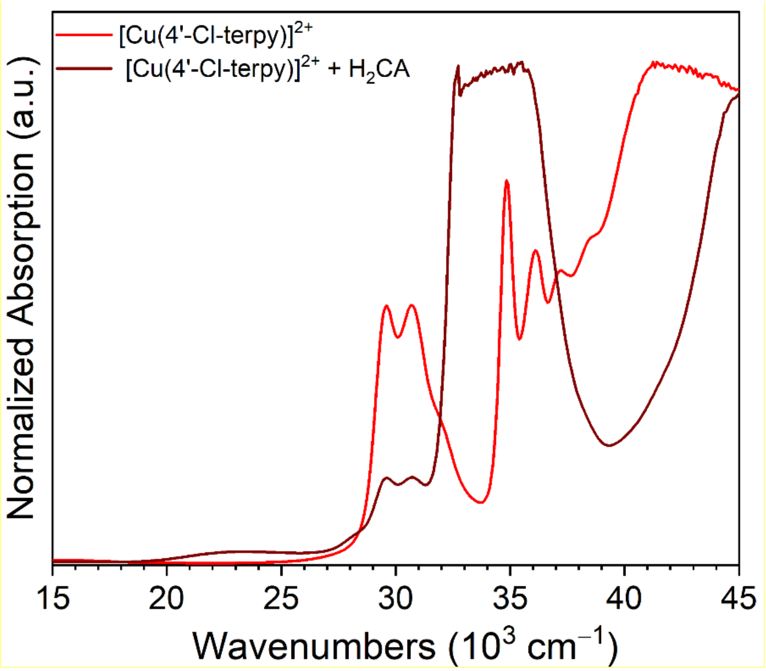

Figure S17. UV-Vis spectra of **7** in MeCN.

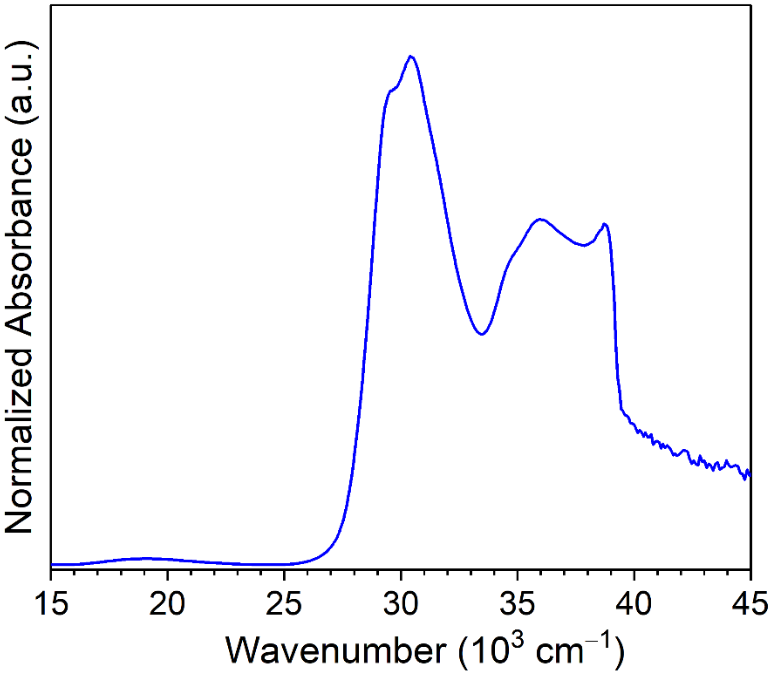

Figure S18. UV-Vis spectrum of **8** in DMSO.

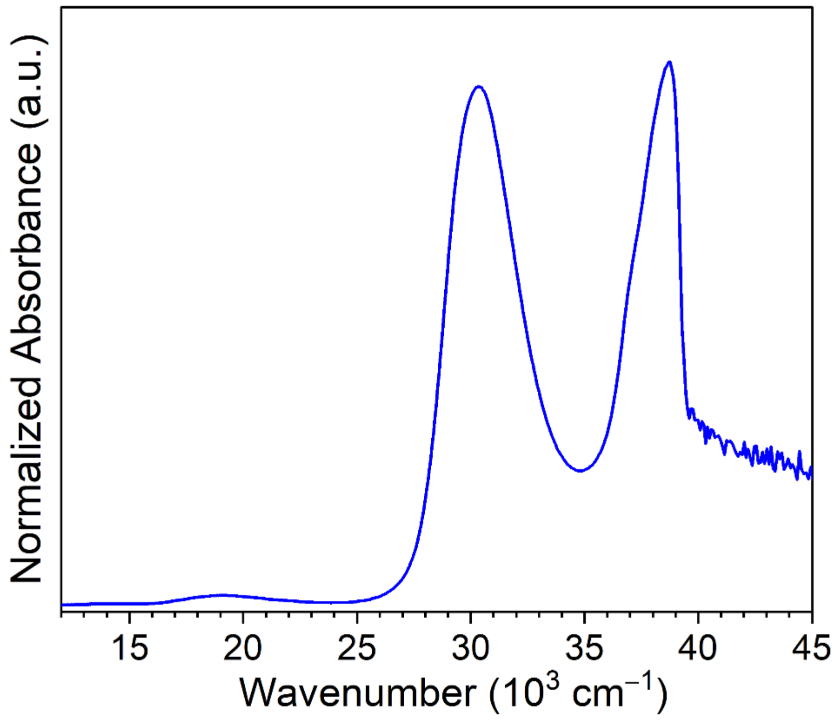

Figure S19. UV-Vis spectrum of **9** in DMSO.

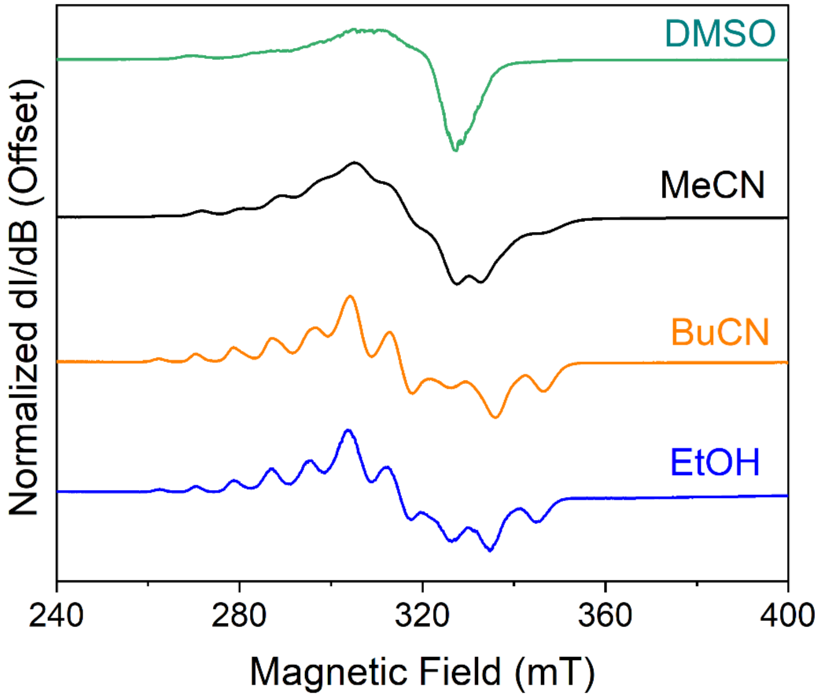

Figure S20. cw EPR spectra of 1 mM solutions of **1** measured in various solvents at 5 K.

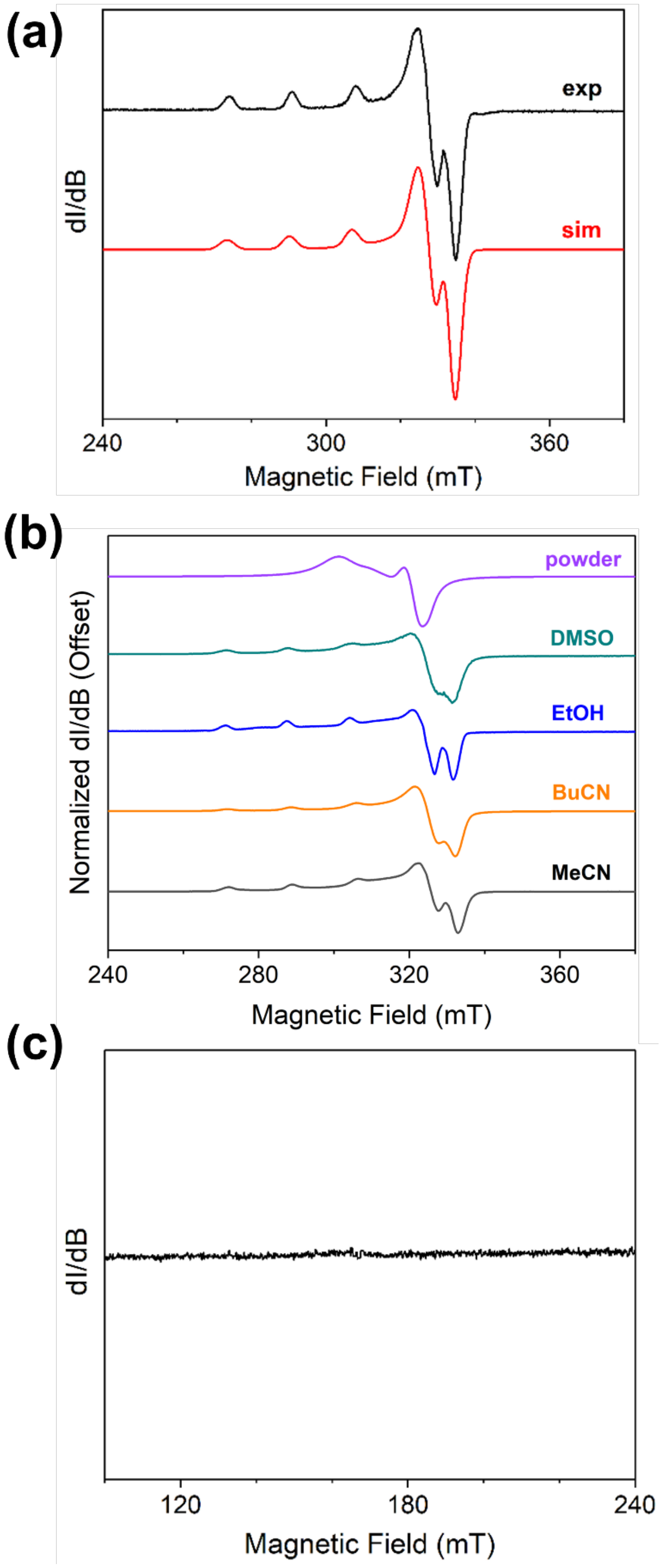

Figure S21. (a) Experimental and simulated X-band cw EPR spectra of **1a** in BuCN at 5 K. **(b)** cw EPR spectra of 1 mM solutions of **1a** in various solvents and as a powder at 5 K. (c) cw EPR spectra of 1 mM solution of **1a** in BuCN measured in parallel mode at 5 K.

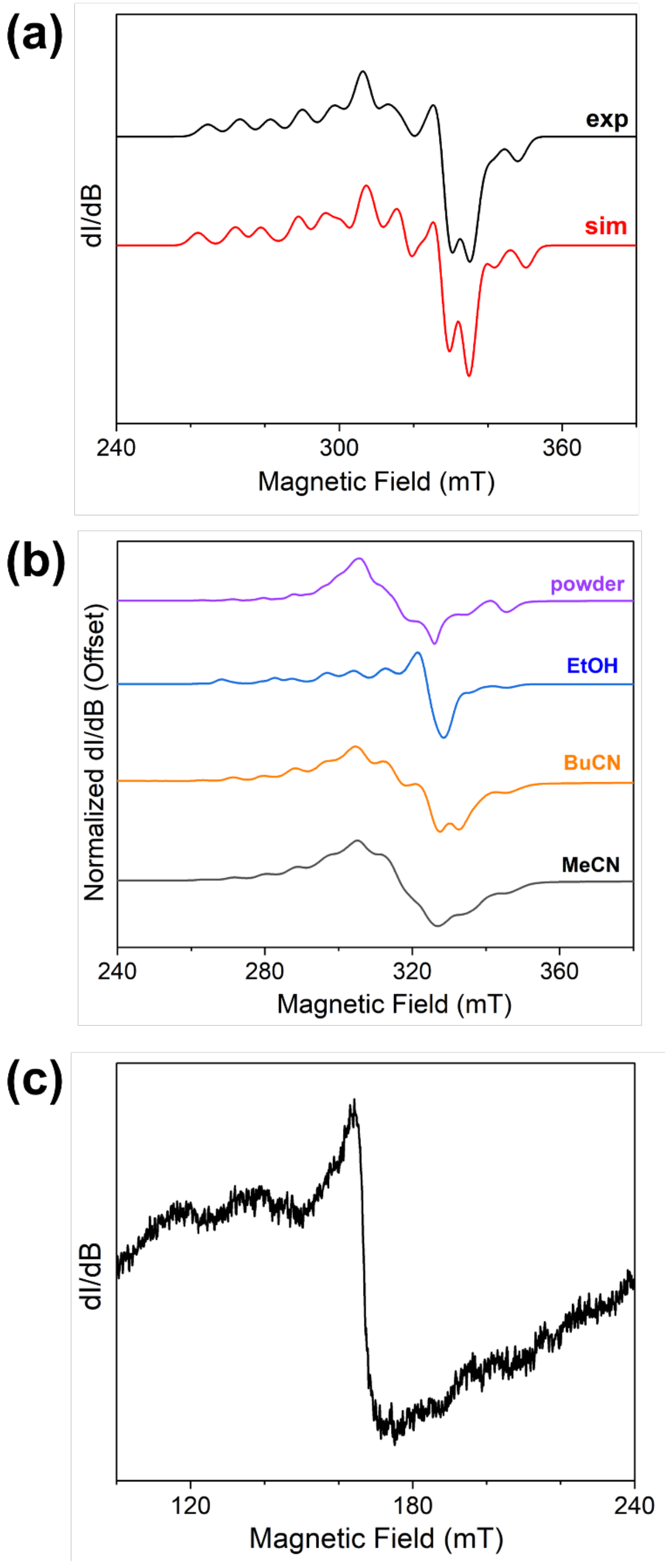

Figure S22. (a) Experimental and simulated X-band cw EPR spectra of **2** in BuCN at 5 K. **(b)** cw EPR spectra of 1 mM solutions of **2** in various solvents and as a powder at 5 K. (c) cw EPR spectra of 1 mM solution of **2** in BuCN measured in parallel mode at 5 K.

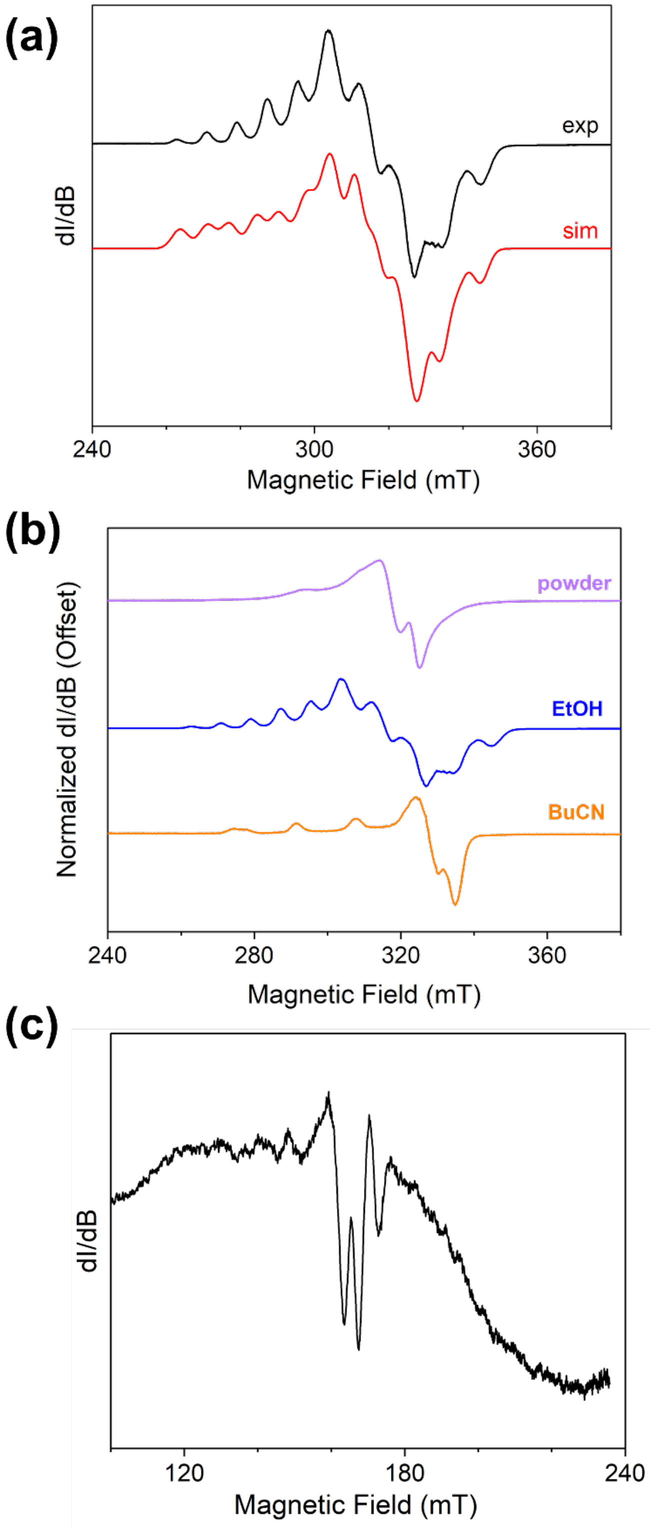

Figure S23. (a) Experimental and simulated X-band cw EPR spectra of **3** in EtOH at 8 K. **(b)** cw EPR spectra of 1 mM solutions of **3** in various solvents and as a powder at 8 K. (c) cw EPR spectra of 1 mM solution of **3** in EtOH measured in parallel mode at 8 K.

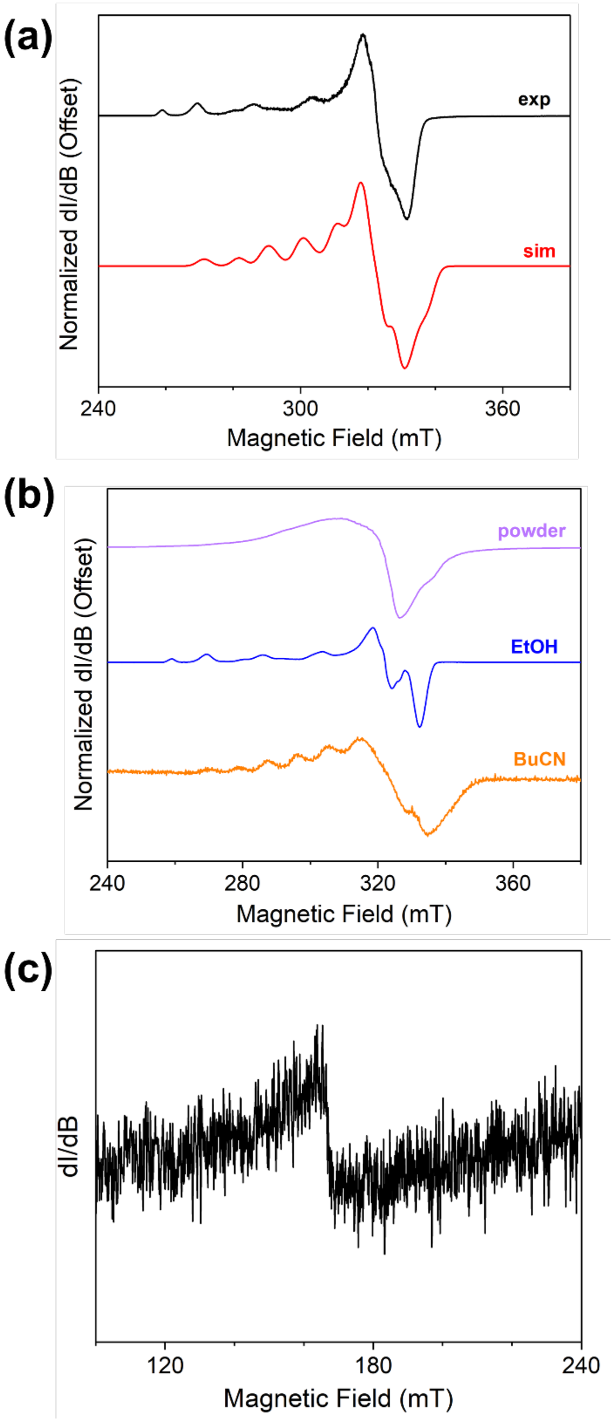

Figure S24. (a) Experimental and simulated X-band cw EPR spectra of **4** in BuCN at 8 K. **(b)** cw EPR spectra of 1 mM solutions of **4** in various solvents and as a powder at 8 K. (c) cw EPR spectra of 1 mM solution of **4** in BuCN measured in parallel mode at 8 K.

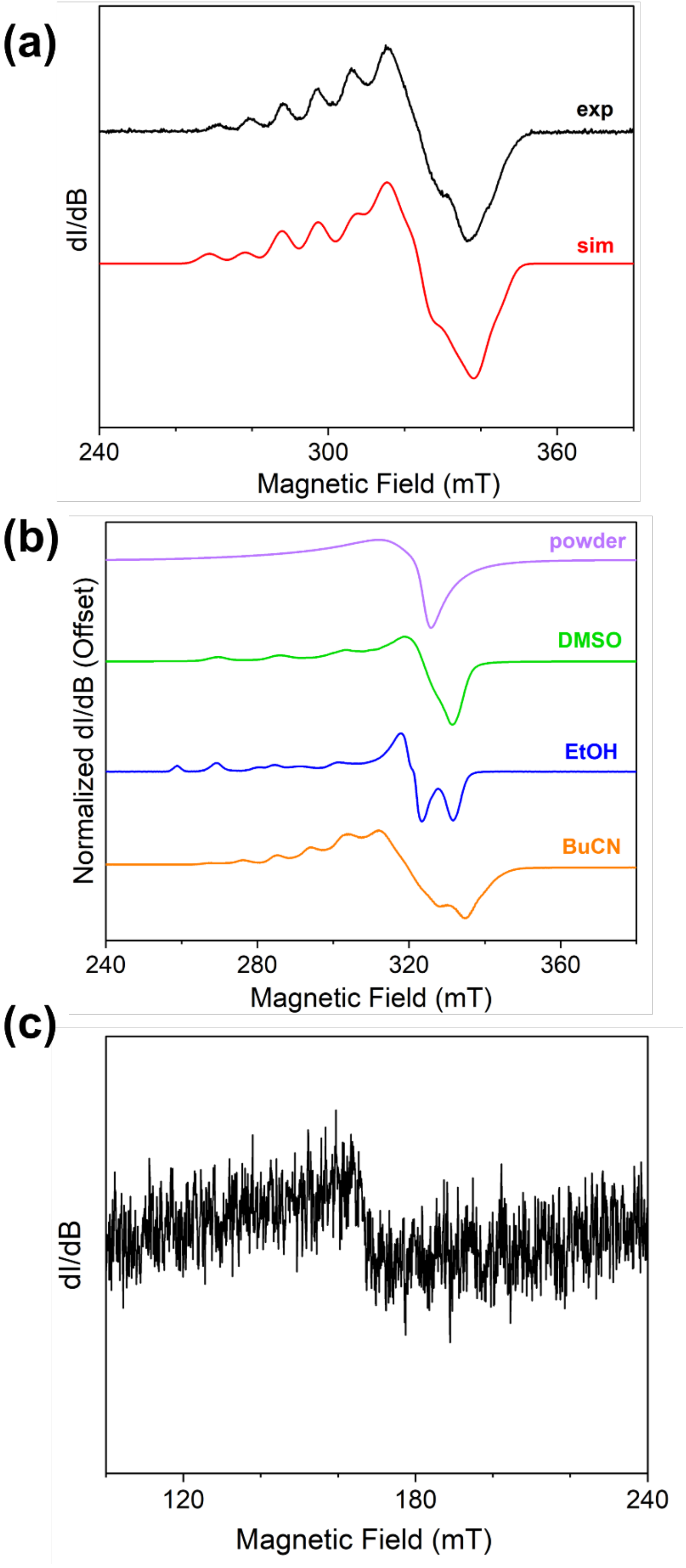

Figure S25. (a) Experimental and simulated X-band cw EPR spectra of **5** in BuCN at 8 K. **(b)** cw EPR spectra of 1 mM solutions of **5** in various solvents and as a powder at 8 K. (c) cw EPR spectra of 1 mM solution of **5** in BuCN measured in parallel mode at 8 K.

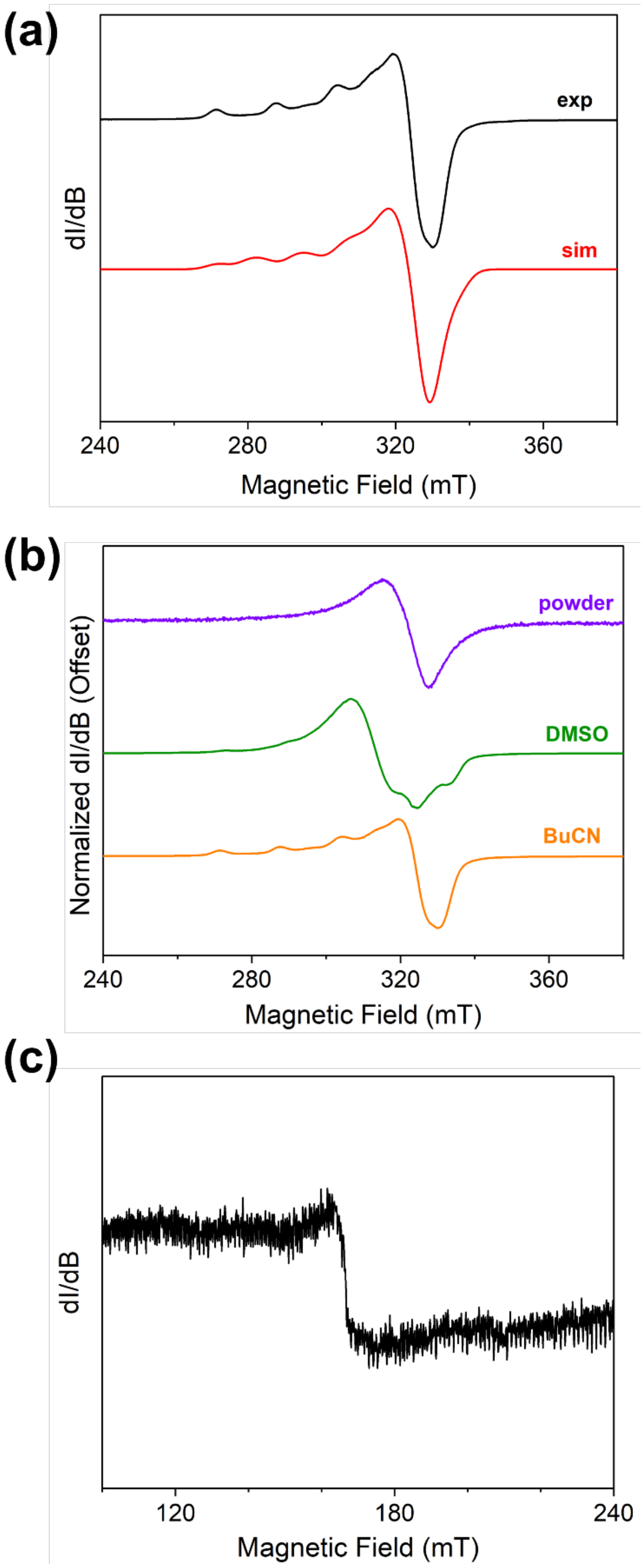

Figure S26. **(a)** Experimental and simulated X-band cw EPR spectra of **6** in BuCN at 8 K. **(b)** cw EPR spectra of 1 mM solutions of **6** in BuCN and DMSO, and as a powder at 8 K. (c) cw EPR spectra of 1 mM solution of **6** in BuCN measured in parallel mode at 8 K.

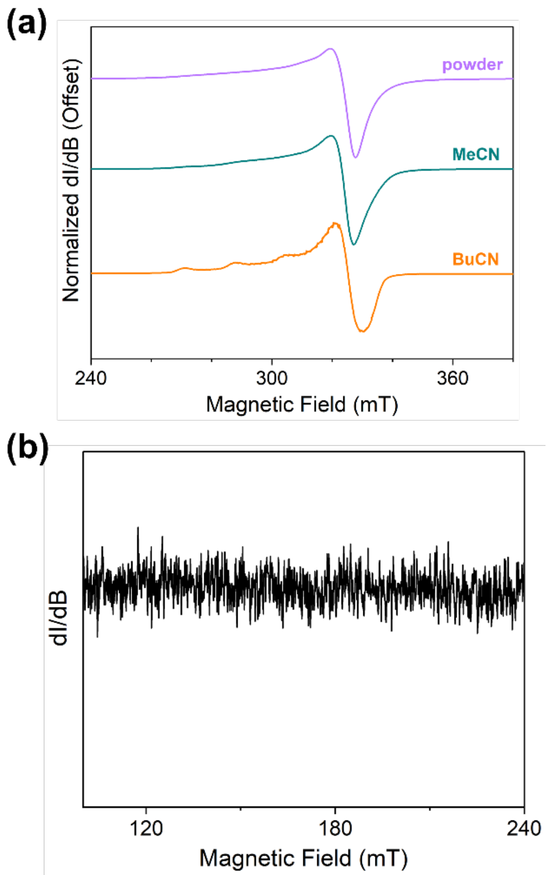

Figure S27. (a) cw EPR spectra of approximately 1 mM solutions of **7** in BuCN and MeCN, and as a powder at 8 K. **(b)** cw EPR spectra of approximately 1 mM solution of **7** in BuCN measured in parallel mode at 8 K.

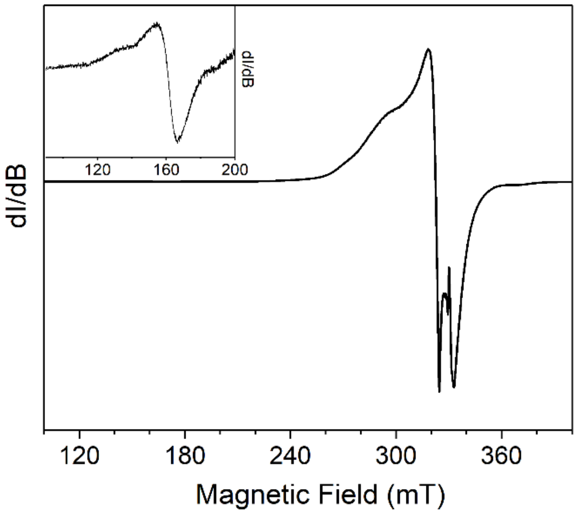

Figure S28. cw EPR spectra of powder sample of **8** measured at 8 K.

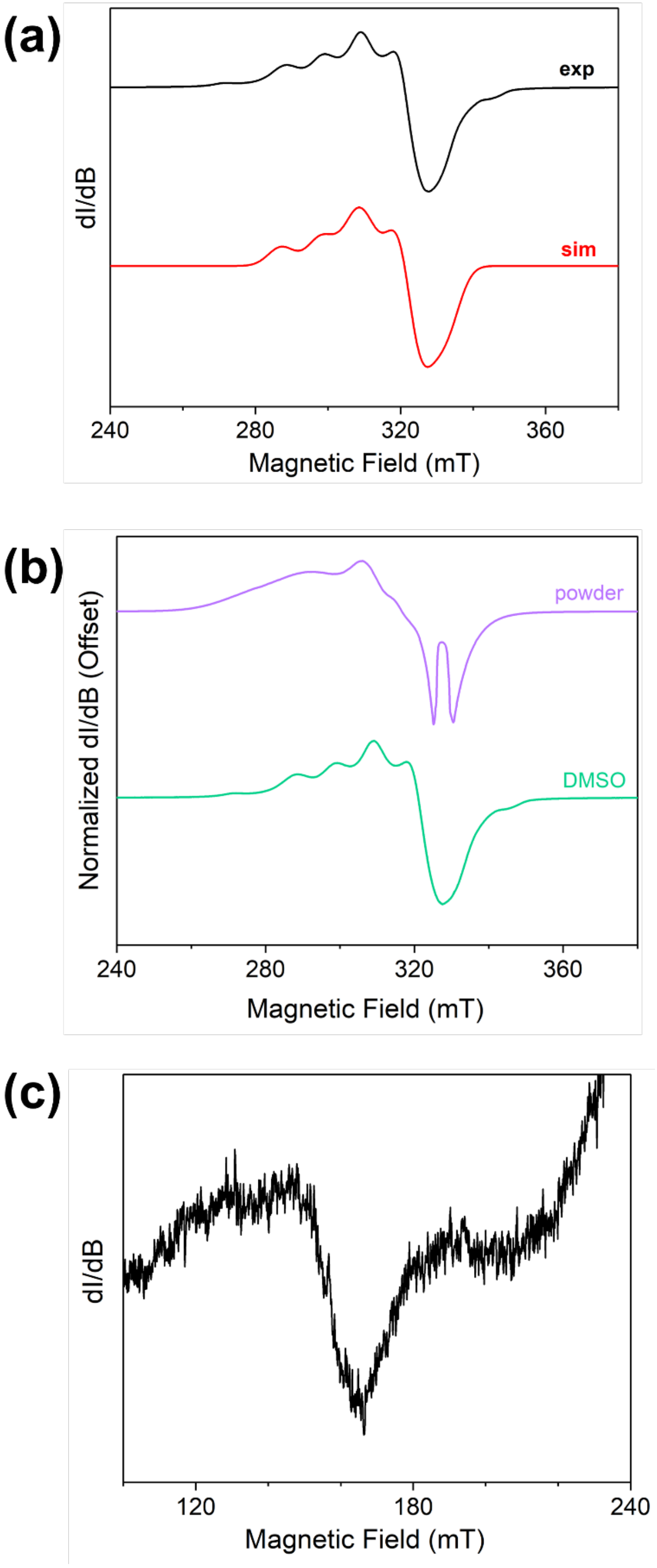

Figure S29. (a) Experimental and simulated X-band cw EPR spectra of **9** in DMSO at 8 K. **(b)** cw EPR spectra of 1 mM solution of **9** in DMSO and as a powder at 8 K. (c) cw EPR spectra of 1 mM solution of **9** in DMSO measured in parallel mode at 8 K.

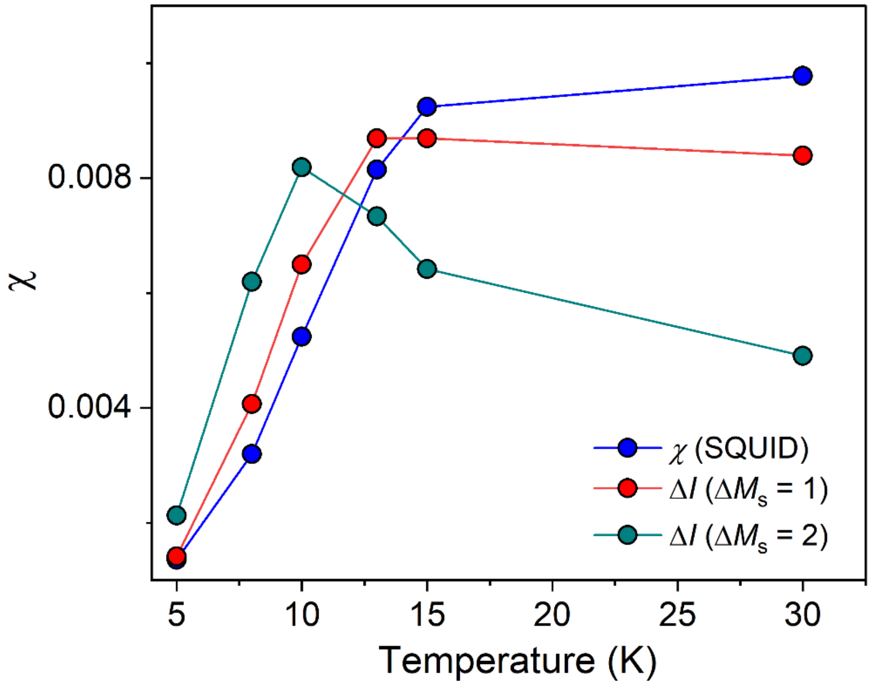

Figure S30. Temperature dependence of magnetic susceptibility (χ, blue; SQUID) and EPR signal intensities for the $\Delta M_s = \pm 1$ (red) and $\Delta M_s = \pm 2$ (green) transitions for compound **1.**

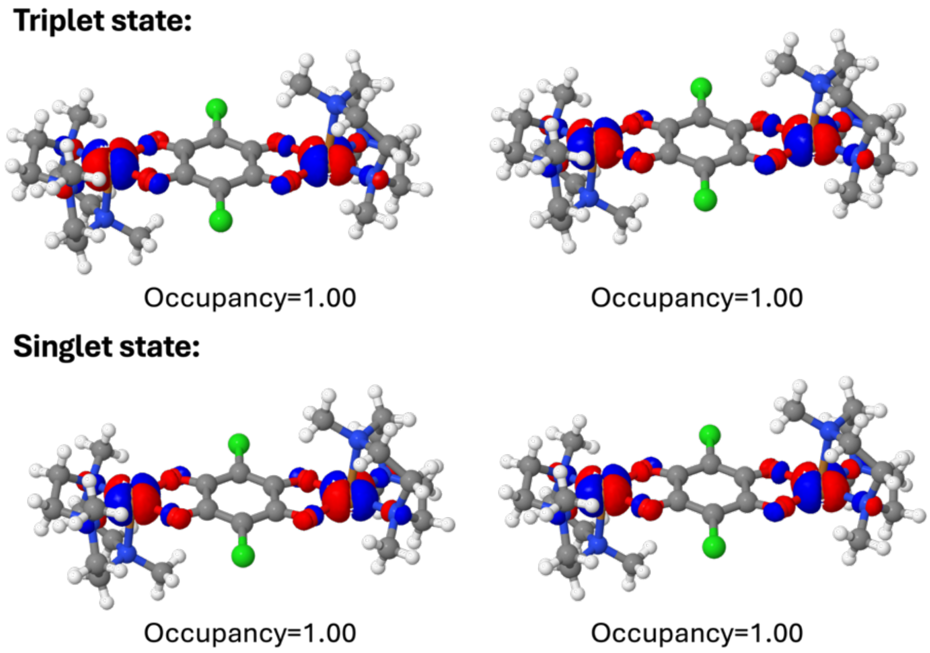

Figure S31. Natural frontier orbitals of complex **1*** obtained from state-specific (18e,10o) CASSCF calculations based on the X-ray structure.

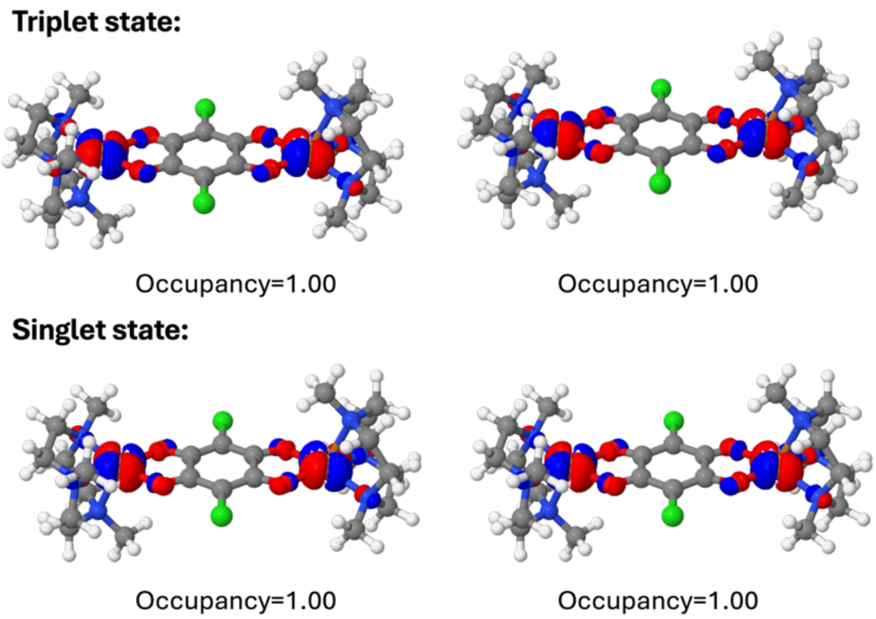

Figure S32. Natural frontier orbitals of complex **1*** obtained from state-specific (10e,10o) CASSCF calculations based on the optimized structure.

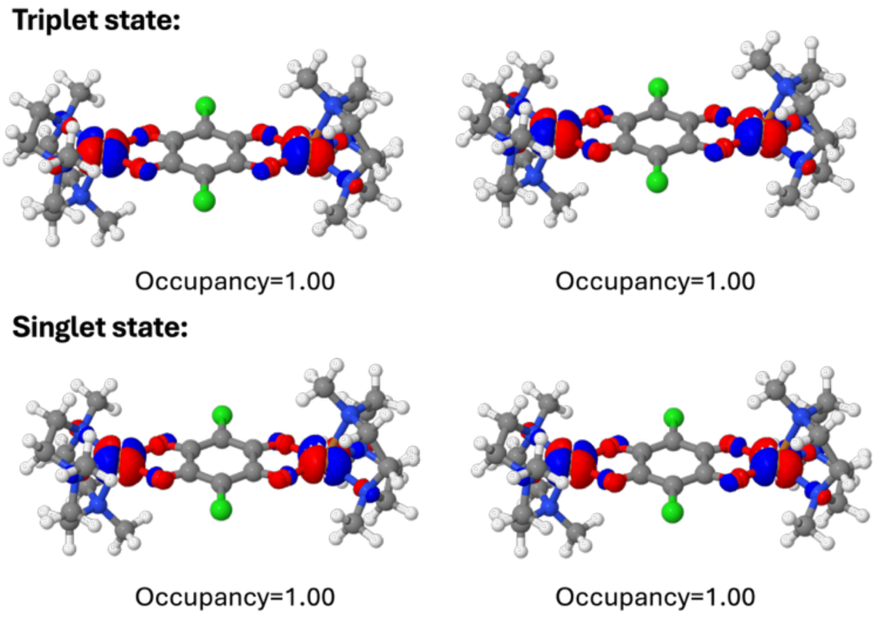

Figure S33. Natural frontier orbitals of complex **1*** obtained from state-specific (18e,10o) CASSCF calculations based on the optimized structure.

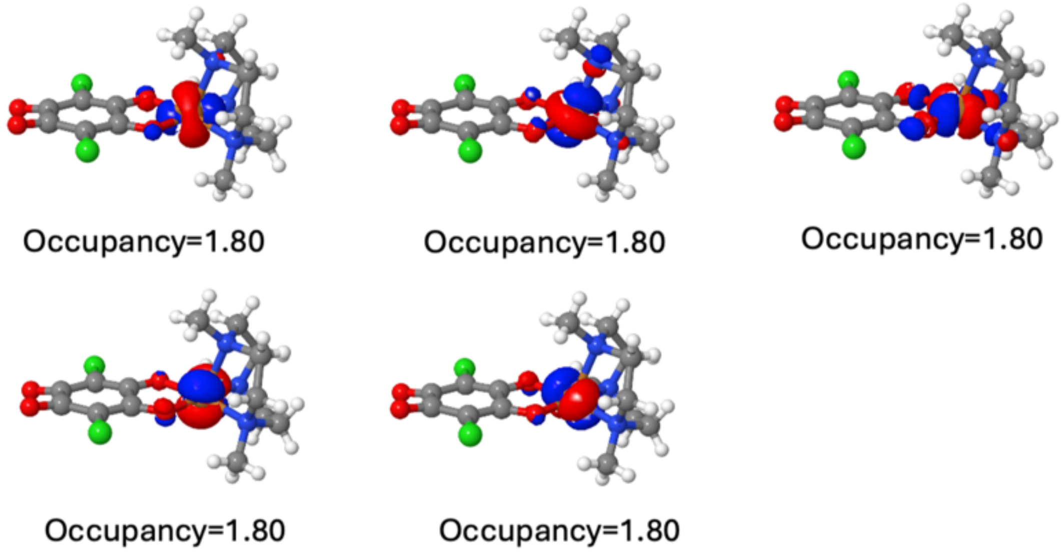

Figure S34. (9e,5o) SA-CASSCF active orbitals of X-ray structure **1a.**

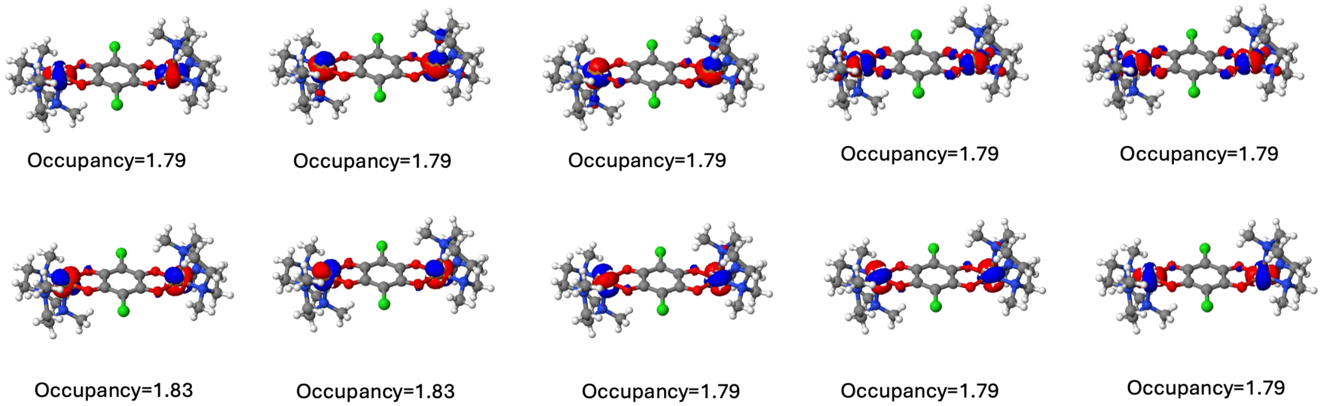

Figure S35. (18e,10o) SA-CASSCF active orbitals of X-ray structure **1***.

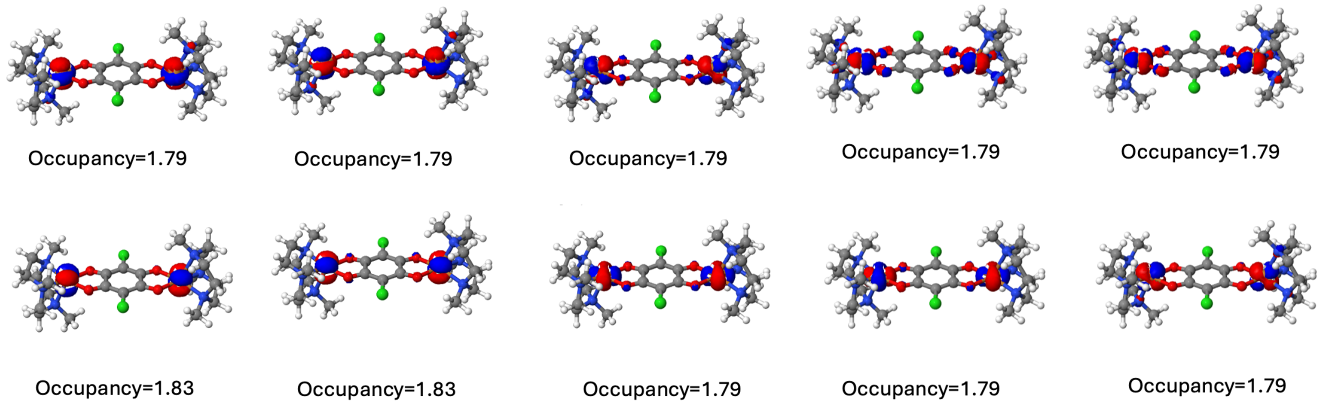

Figure S36. (18e,10o) SA-CASSCF active orbitals of optimized structure **1*.**

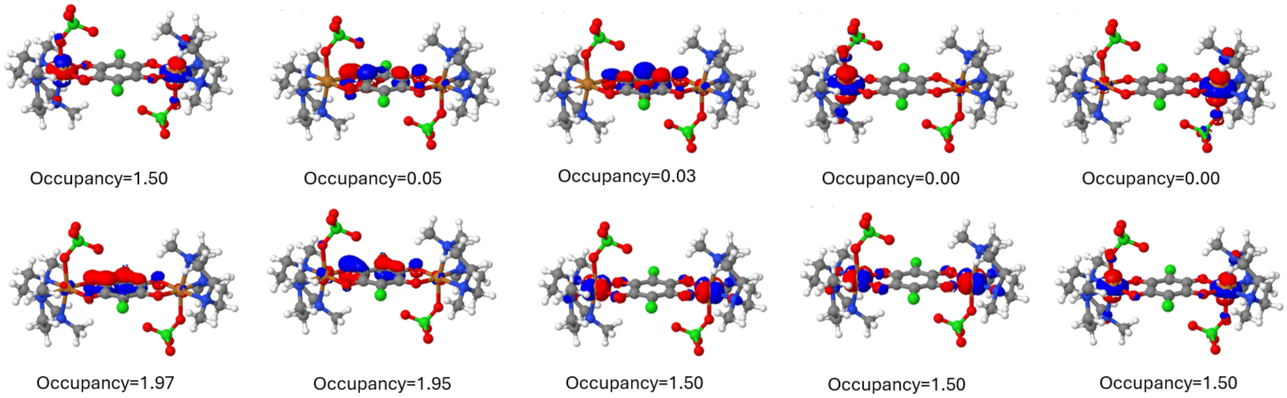

Figure S37. (10e,10o) SA-CASSCF active orbitals of optimized structure **1**.

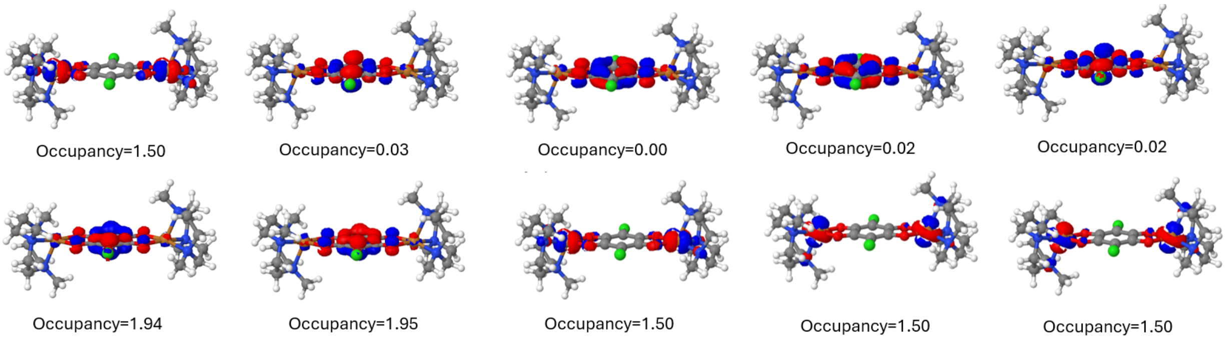

Figure S38. (10e,10o) SA-CASSCF active orbitals of optimized structure **1***.

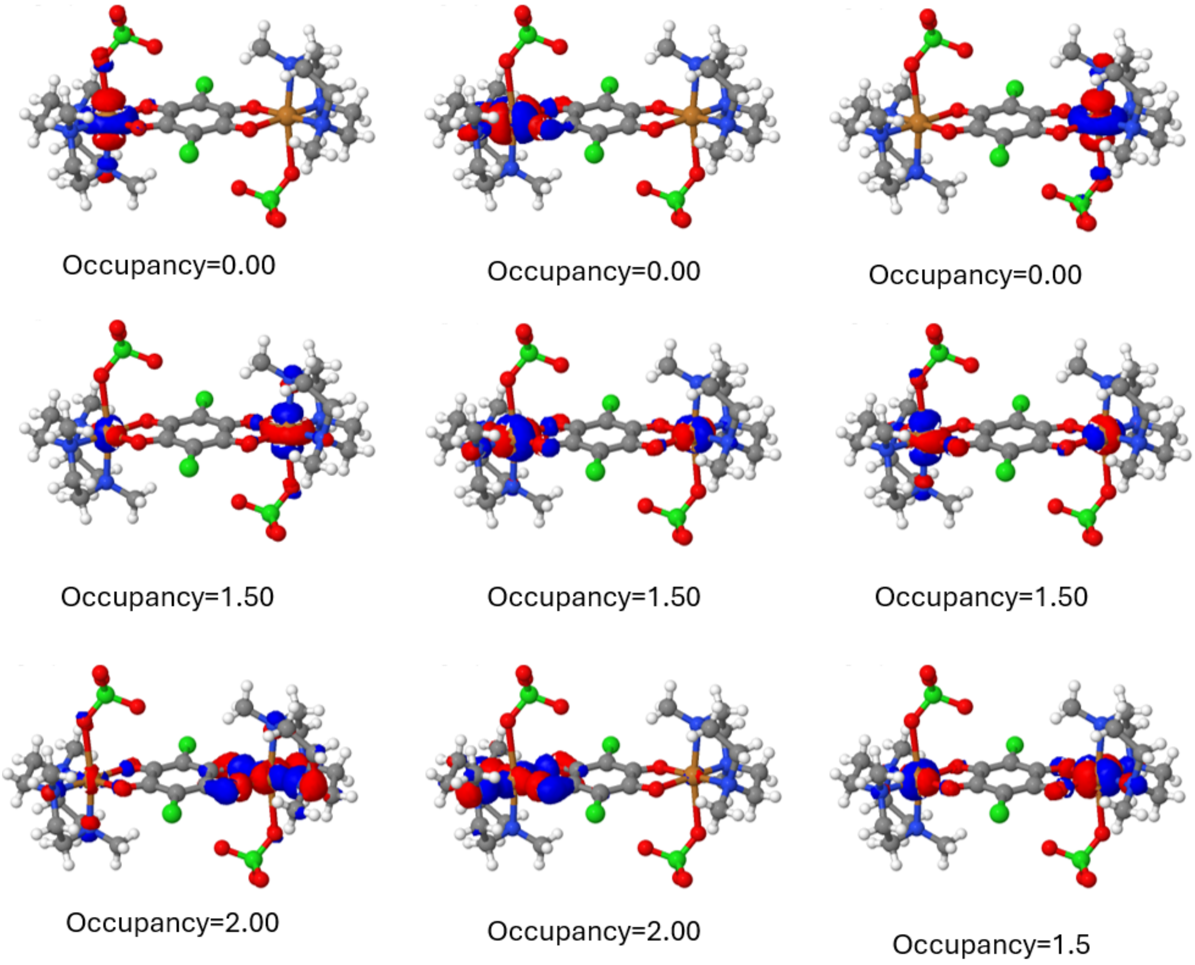

Figure S39. (10e,9o) SA-CASSCF active orbitals of X-ray structure **1**.

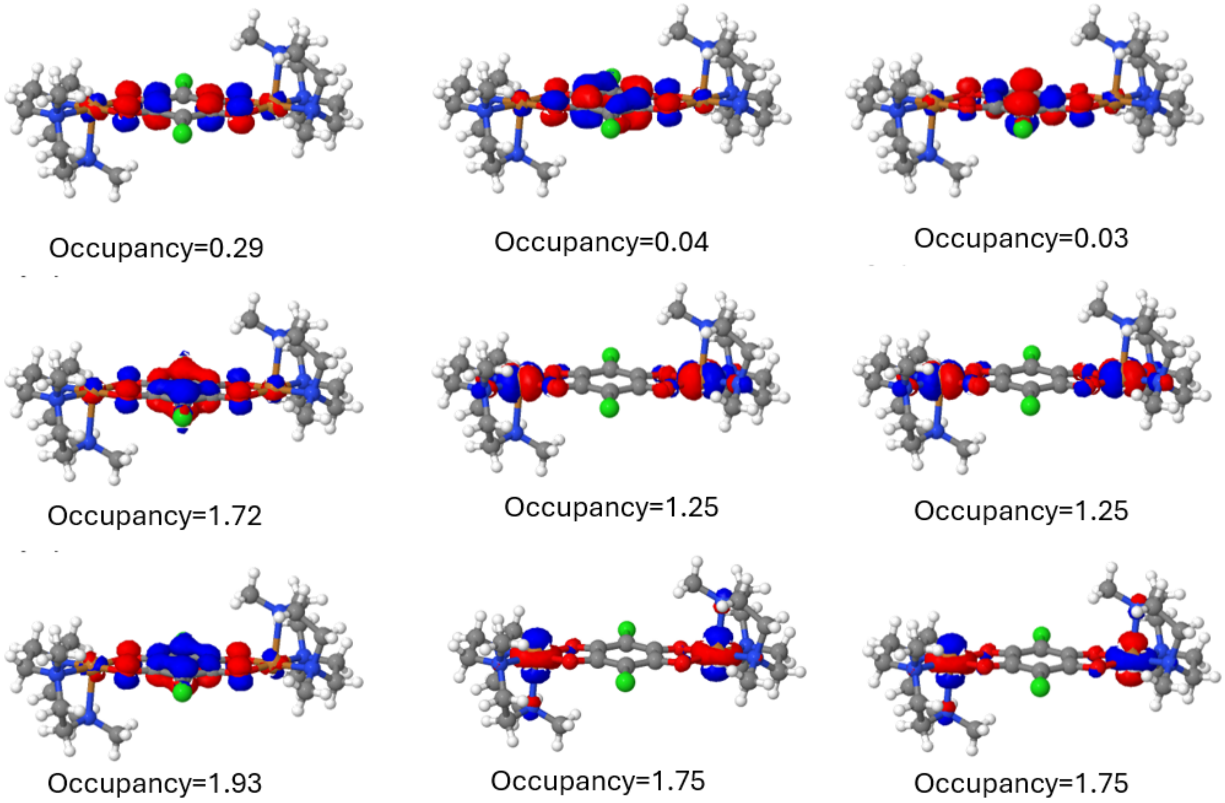

Figure S40. (9o,10e) SA-CASSCF active orbitals of X-ray structure **1***.

Additional Vibrational Analysis. Infrared spectroscopy was employed to assess ligand coordination, confirm chloroanilate (CA) binding modes, and identify uncoordinated counterions across complexes **1-6**. A summary of diagnostic vibrational bands is discussed below:

Across complexes **1-3**, the IR data consistently support Me_3tacn coordination, CA binding, and the presence of distinct counterions. Me_3tacn gives the expected C–N stretches in the 1100-1300 cm^{-1} region and methyl C–H modes near 2800-2950 cm^{-1}, with additional C-H bending around 1380 cm^{-1}. CA coordination is evident from the characteristic, red-shifted C=O and C=C envelope spanning ~1450-1600 cm^{-1} (e.g., 1508-1530 cm^{-1} across the series), accompanied by C–Cl stretches below 900 cm^{-1}.[1-3] Metal-ligand vibrations consistently appear between 400-600 cm^{-1}, with occasional lower-frequency Cu–Cl features (300-400 cm^{-1}) when present. The counterions remain spectroscopically distinct: complex **1** exhibits strong ClO_4^- absorptions at ~620 and ~1095 cm^{-1}, whereas complexes **2** and **3** show the characteristic $CF_3SO_3^-$ S=O and C–F stretching bands at 1030–1240 and 740–790 cm^{-1}, respectively. A broad O–H band near 3375 cm^{-1} in complex **3** further indicates the presence of coordinated water.[4]

Within the tmchd series (**4-6**), the IR spectra show the expected ligand signatures and differentiate the counterions. All three complexes display the characteristic tmchd methyl and cyclohexane C-H stretches at 2825-2976 cm^{-1} and N–CH_3 bending modes between 1400-1500 cm^{-1}[5], while CA^{2-} coordination is evidenced by the 1450-1600 cm^{-1} C=O and C=C envelope. Complex **4** exhibits Cu–O stretches at 418, 514, and 571 cm^{-1} together with strong ClO_4^- absorptions at ~620 and 1061 cm^{-1}, consistent with an uncoordinated or weakly interacting perchlorate. Complex **5** displays the diagnostic $CF_3SO_3^-$ features, S=O stretches at 1250-1300 cm^{-1}, the SO_3 band at 1030 cm^{-1}, and C–F modes at 750-800 cm^{-1} accompanied by Cu–N vibrations below 600 cm^{-1} that confirm coordination. In **6**, the presence of low-frequency Cu–Cl features (300-400 cm^{-1}) support the assignment of chloride as the counterion.

Across the full series, the IR data provide a consistent picture of CA-bridged Cu(II) coordination and well-defined ligand and counterion environments. CA binding is uniformly marked by the red-shifted C=O/C=C envelope in the 1450-1600 cm^{-1} region, together with the characteristic C–Cl stretches below 900 cm^{-1}. Metal-ligand coordination is further supported by Cu–N and Cu–O vibrations appearing reliably between 400-600 cm^{-1}, with additional low-frequency features (300-400 cm^{-1}) assigned to Cu-Cl when present. Counterions remain spectroscopically distinct and unperturbed across the series: ClO_4^- exhibits its strong absorptions at ~620 and ~1060-1100 cm^{-1}, whereas $CF_3SO_3^-$ is identified by the intense S=O and C–F modes in the 1030-1300 and 740-790 cm^{-1} windows. The auxiliary ligands Me_3tacn and tmchd contribute predictable C–H and N–CH_3 features without obscuring the diagnostic CA^{2-} signatures. Taken together, these vibrational trends validate the proposed coordination environment around each Cu(II) center, reinforce the bridging role of the CA^{2-} ligand, and confirm the persistence of distinct, noncoordinating and coordinating counterions throughout the series.

References

(1) Alves, W. A.; Santos, R. H. d. A.; Paduan-Filho, A.; Becerra, C. C.; Borin, A. C.; Ferreira, A. M. D. C. Molecular Structure and Intra- and Intermolecular Magnetic Interactions in Chloro-Bridged Copper(II) Dimers. *Inorg. Chim. Acta.* **2004**, *357* (8), 2269-2278. DOI: 10.1016/j.ica.2004.01.004.
(2) Folgado, J. V.; Ibanez, R.; Coronado, E.; Beltran, D.; Savariault, J. M.; Galy, J. Extremely Weak Magnetic Exchange Interactions in Terpy-Containing Copper(II) Dimer. Crystal and Molecular Structure of Cu(terpy)(CA)·H_2O and $Cu_2(terpy)_2(CA)_2$ Complexes (terpy = 2,2′:6′,2″-Terpyridine, CA = Dianion of Chloranilic Acid). *Inorg. Chem.* **2002**, *27* (1), 19-26. DOI: 10.1021/ic00274a007.
(3) Gallert, S.; Weyhermüller, T.; Wieghardt, K.; Chaudhuri, P. Exchange Coupling Across Multiple-Atom Bridges: Crystal Structure and Magnetic Properties of a Chloranilate Bridged Dicopper(II) Complex. *Inorg. Chim. Acta.* **1998**, *274* (1), 111-114. DOI: 10.1016/s0020-1693(97)05959-8.
(4) Cozar, O.; David, L.; Chiş, V.; Damian, G.; Todică, M.; Agut, C. IR and ESR Studies On Some Dimeric Copper(II) Complexes. *J. Mol. Struct.* **2001**, *563-564*, 371-375. DOI: 10.1016/s0022-2860(01)00496-3.
(5) Cole, A. P.; Mahadevan, V.; Mirica, L. M.; Ottenwaelder, X.; Stack, T. D. Bis(μ-oxo)dicopper(III) Complexes of a Homologous Series of Simple Peralkylated 1,2-Diamines: Steric Modulation of Structure, Stability, and Reactivity. *Inorg. Chem.* **2005**, *44* (21), 7345-7364. DOI: 10.1021/ic050331i.
